# Comparative accuracy of ChatGPT-4, Microsoft Copilot and Google Gemini in the Italian entrance test for healthcare sciences degrees: a cross-sectional study

**DOI:** 10.1186/s12909-024-05630-9

**Published:** 2024-06-26

**Authors:** Giacomo Rossettini, Lia Rodeghiero, Federica Corradi, Chad Cook, Paolo Pillastrini, Andrea Turolla, Greta Castellini, Stefania Chiappinotto, Silvia Gianola, Alvisa Palese

**Affiliations:** 1 School of Physiotherapy, University of Verona, Verona, Italy; 2 Department of Physiotherapy, Faculty of Sport Sciences, Universidad Europea de Madrid, Villaviciosa de Odón, 28670 Spain; 3 Department of Rehabilitation, Hospital of Merano (SABES-ASDAA), Teaching Hospital of Paracelsus Medical University (PMU), Merano-Meran, Italy; 4 School of Speech Therapy, University of Verona, Verona, Italy; 5 Department of Orthopaedics, Duke University, Durham, NC USA; 6 Duke Clinical Research Institute, Duke University, Durham, NC USA; 7 Department of Population Health Sciences, Duke University, Durham, NC USA; 8 Department of Biomedical and Neuromotor Sciences (DIBINEM), Alma Mater University of Bologna, Bologna, Italy; 9 Unit of Occupational Medicine, IRCCS Azienda Ospedaliero-Universitaria Di Bologna, Bologna, Italy; 10 Unit of Clinical Epidemiology, IRCCS Istituto Ortopedico Galeazzi, Milan, Italy; 11 Department of Medical Sciences, University of Udine, Udine, Italy

**Keywords:** Artificial intelligence, Students, Health occupations, Learning, Education, Nursing, Education, Medical, Nursing, Physical therapy modalities, Speech therapy, Midwifery

## Abstract

**Background:**

Artificial intelligence (AI) chatbots are emerging educational tools for students in healthcare science. However, assessing their accuracy is essential prior to adoption in educational settings. This study aimed to assess the accuracy of predicting the correct answers from three AI chatbots (ChatGPT-4, Microsoft Copilot and Google Gemini) in the Italian entrance standardized examination test of healthcare science degrees (CINECA test). Secondarily, we assessed the narrative coherence of the AI chatbots’ responses (i.e., text output) based on three qualitative metrics: the logical rationale behind the chosen answer, the presence of information internal to the question, and presence of information external to the question.

**Methods:**

An observational cross-sectional design was performed in September of 2023. Accuracy of the three chatbots was evaluated for the CINECA test, where questions were formatted using a multiple-choice structure with a single best answer. The outcome is binary (correct or incorrect). Chi-squared test and a post hoc analysis with Bonferroni correction assessed differences among chatbots performance in accuracy. A *p*-value of < 0.05 was considered statistically significant. A sensitivity analysis was performed, excluding answers that were not applicable (e.g., images). Narrative coherence was analyzed by absolute and relative frequencies of correct answers and errors.

**Results:**

Overall, of the 820 CINECA multiple-choice questions inputted into all chatbots, 20 questions were not imported in ChatGPT-4 (*n* = 808) and Google Gemini (*n* = 808) due to technical limitations. We found statistically significant differences in the ChatGPT-4 vs Google Gemini and Microsoft Copilot vs Google Gemini comparisons (*p*-value < 0.001). The narrative coherence of AI chatbots revealed “Logical reasoning” as the prevalent correct answer (*n* = 622, 81.5%) and “Logical error” as the prevalent incorrect answer (*n* = 40, 88.9%).

**Conclusions:**

Our main findings reveal that: (A) AI chatbots performed well; (B) ChatGPT-4 and Microsoft Copilot performed better than Google Gemini; and (C) their narrative coherence is primarily logical. Although AI chatbots showed promising accuracy in predicting the correct answer in the Italian entrance university standardized examination test, we encourage candidates to cautiously incorporate this new technology to supplement their learning rather than a primary resource.

**Trial registration:**

Not required.

**Supplementary Information:**

The online version contains supplementary material available at 10.1186/s12909-024-05630-9.

## Background

Being enrolled in a healthcare science degree in Italy requires a university examination, which is a highly competitive and selective process that demands intensive preparation worldwide [1]. Conventional preparation methods involve attending classes, studying textbooks, and completing practical exercises [2]. However, with the emergence of artificial intelligence (AI), digital tools like AI chatbots to assist in exam preparation are becoming more prevalent, presenting novel opportunities for candidates [2].

AI chatbots such as ChatGPT, Microsoft Bing, and Google Bard are advanced language models that can produce responses similar to humans through a user-friendly interface [3]. These chatbots are trained using vast amounts of data and deep learning algorithms, which enable them to generate coherent responses and predict text by identifying the relationships between words [3]. Since their introduction, AI chatbots have gained considerable attention and sparked discussions in medical and health science education and clinical practice [4–7]. AI chatbots can provide simulations with digital patients, personalized feedback, and help eliminate language barriers; they also present biases, ethical and legal concerns, and content quality issues [8, 9]. As such, the scientific community recommends evaluating the AI chatbot’s accuracy of predicting the correct answer (e.g., passing examination tests) to inform students and academics of their value [10, 11].

Several studies have assessed the accuracy of AI chatbots to pass medical education tests and exams. A recent meta-analysis found that ChatGPT-3.5 correctly answered most multiple-choice questions across various medical educational fields [12]. Further research has shown that newer versions of AI chatbots, such as ChatGPT-4, have surpassed their predecessors in passing Specialty Certificate Examinations in dermatology [13, 14], neurology [15], ophthalmology [16], rheumatology [17], general medicine [18–21], and nursing [22]. Others have reported mixed results when comparing the accuracy of multiple AI chatbots (e.g., ChatGPT-4 vs Microsoft Bing, ChatGPT-4 vs Google Bard) in several medical examinations tests [23–29]. Recently, two studies observed the superiority of ChatGPT-3.5 over Microsoft Copilot and Google Bard in hematology [30] and physiology [31] case solving. Recent work has also observed that ChatGPT-4 outperformed other AI Chatbots in clinical dentistry-related questions [32], whereas another revealed that ChatGPT-4 and Microsoft Bing outperformed Google Bard and Claude in the Peruvian National Medical Licensing Examination [33].

These findings suggest a potential hierarchy in accuracy of AI chatbots, although continued study in medical education is certainly warranted [3]. Further, current studies are limited by predominantly investigating: (A) a single AI chatbot rather than multiple ones; (B) examination tests for students and professionals already in training rather than newcomers to the university; and (C) examination tests for medical specialities rather than for healthcare science (e.g., rehabilitation and nursing). Only two studies [34, 35] have attempted to address these limitations, identifying ChatGPT-3.5 as a promising, supplementary tool to pass several standardised admission tests in universities in the UK [34] and in France [35]. To our knowledge, no study has been performed on admission tests for admissions to a healthcare science degree program. Healthcare Science is a profession that includes over 40 areas of applied science that support the diagnosis, rehabilitation and treatment of several clinical conditions [36]. Moreover, the only studies conducted in Italy concerned ChatGPT's accuracy in passing the Italian Residency Admission National Exam for medical graduates [37, 38] offering opportunities for further research setting.

Accordingly, to overcome existing knowledge gaps, this study aimed to assess the comparative accuracy of predicting the correct answer of three updated AI chatbots (ChatGPT-4, Microsoft Copilot and Google Gemini) in the Italian entrance university standardized examination test of healthcare science. The secondary aim was to assess the narrative coherence of the text responses offered by the AI chatbots. Narrative coherence was defined as the internally consistency and sensibility of the internal or external explanation provided by the chatbot.

## Methods

### Study design and ethics

We conducted an observational cross-sectional study following the Strengthening of Reporting of Observational Studies in Epidemiology (STROBE) high-quality reporting standards [39]. Because no human subjects were included, ethical approval was not required [40].

### Setting

This study was developed by an Italian multidisciplinary group of healthcare science educators. The group included professors, lecturers, and educators actively involved in university education in different healthcare disciplines (e.g., rehabilitation, physiotherapy, speech therapy, nursing).

### Sample

In Italy, the university’s process of accessing the healthcare professions is regulated by the laws according to short- and long-term workforce needs [41]. Consequently, the placements available for each degree are established in advance; to be enrolled in an academic year, candidates should take a standardized examination test occurring on the same day for all universities. This process, in most Italian universities, is annually managed by the CINECA (Consorzio Interuniversitario per il Calcolo Automatico dell'Italia Nord Orientale), a governmental organization composed of 70 Italian universities, 45 national public research centers, the Italian Ministry of University and Research, and the Italian Ministry of Education [42]. CINECA prepares the standardized test common to all healthcare disciplines (e.g., nursing and midwifery, rehabilitation, diagnostics and technical, and prevention) for entrance to University [43]. The test assesses basic knowledge useful as a prerequisite for their future education [44], in line with the expected knowledge possessed by candidates that encompass students at the end of secondary school, including those from high schools, technical, and professional institutes [45].

For this study, we adopted the official CINECA Tests from the past 13 years (2011–2023) obtained from freely available public repositories [46, 47]. The CINECA Test provided 60–80 range of independent questions per year for a total of 820 multiple-choice questions considered for the analysis. Every question presents five multiple-choice options, with only one being the correct answer and the remaining four being incorrect [44]. According to the law, over the years, the CINECA test consisted of multiple-choice questions covering four areas: (1) logical reasoning and general culture, (2) biology, (3) chemistry, and (4) physics and mathematics. The accuracy of each AI chatbot was evaluated as the sum of the proportion of correct answers provided among all possible responses for each area and for the total test. In Additional file 1, we reported all the standardized examination tests used in the Italian language and an example of the question stem that was exactly replicated.

### Variable and measurements

We assessed the accuracy of three AI chatbots in providing accurate responses for the Italian entrance university standardized examination test for healthcare disciplines. We utilized the latest versions of ChatGPT-4 (OpenAI Incorporated, Mission District, San Francisco, United States) [48], Microsoft Copilot (Microsoft Corporation, WA, US) [49] and Google Gemini (Alphabet Inc., CA, US) [50] that were updated in September 2023. We considered the following variables: (A) the accuracy of predicting the correct answer of the three AI chatbots in the CINECA Test and (B) the narrative coherence and errors of the three AI chatbots responses.

The accuracy of three AI chatbots was assessed by comparing their responses to the correct answers from the CINECA Test. AI Chatbots’ answers were entered into an Excel sheet and categorized as correct or incorrect. Ambiguous or multiple responses were marked as incorrect [51]. Since none of the three chatbots has integrated multimodal input at this point, questions containing imaging data were evaluated based solely on the text portion of the question stem. However, technical limitations can be present, and a sensitivity analysis was performed, excluding answers that were not applicable (e.g., images).

The narrative coherence and errors [52] of AI chatbot answers for each question were assessed using a standardized system for categorization [53]. Correct answers were classified as [53]: (A) “Logical reasoning”, if they clearly demonstrated the logic presented in the response; (B) “Internal information”, if they included information from the question itself; and (C) “External information”, if they referenced information external to the question.

On the other side, incorrect answers were categorized as [53]: (A) “Logical error”, when they correctly identify the relevant information but fail to convert it into an appropriate answer; (B) “Information error”, if AI chatbots fail to recognize a key piece of information, whether present in the question stem or through external information; and (C) “Statistical error”, for arithmetic mistakes. An example of categorisation is displayed in Additional file 2. Two authors (L.R., F.C.) independently analyzed the narrative coherence, with a third (G.R.) resolving uncertainties. Inter-rater agreement was measured using Cohen’s Kappa, according to the scale offered by Landis and Koch: < 0.00 “poor”, 0–0.20 “slight”; 0.21–0.40 “fair”, 0.41–0.60 “moderate”, 0.61–0.80 “substantial”, 0.81–1.00 “almost perfect” [54].

### Procedure

We used each multiple-choice question of the CINECA Test, formatted for proper structure and readability. Because prompt engineering significantly affects generative output, we standardized the input formats of the questions following the Prompt-Engineering-Guide [55, 56]. First, we manually entered each question in a Word file, left one line of space and then inserted the five answer options one below the other on different lines. If the questions presented text-based answers, they were directly inputted into the 3 AI chatbots. If the questions were presented as images containing tables or mathematical formulae, they were faithfully rewritten for AI chatbot processing [57]. If the answers had images with graphs or drawings, they were imported only into Microsoft Copilot because ChatGPT-4 and Google Gemini only accept textual input in their current form and could not process and interpret the meaning of complex images, as present in the CINECA Test, at the time of our study [58].

On 26th of September 2023, the research group copied and pasted each question onto each of the 3 AI chatbots in the same order in which it was presented in the CINECA Test [59] and without translating it from the original Italian language to English because the AIs are language-enabled [60]. To avoid learning bias and that the AI chatbots could learn or be influenced by conversations that existed before the start of the study, we: (A) created and used a new account [2, 51], (B) always asked each question only once [61, 62], (C) did not provide positive or negative feedback on the answer given [60], and (D) deleted conversations with the AI chatbots before entering each new question into a new chat (with no previous conversations). We presented an example of a question and answer in Additional file 3.

### Statistical analyses

Categorical variables are presented as the absolute frequency with percent and continuous variables as mean with confidence interval (CI, 95%) or median with interquartile range (IQR). The answers were collected as binomial outcomes for each AI chatbot respect to the reference (CINECA Tests). A chi-square test was used to ascertain whether the CINECA Test percentage of correct answers differed among the three AI chatbots according to different taxonomic subcategories (logical reasoning and general culture, biology, chemistry, and physics and mathematics). A sensitivity analysis was performed, excluding answers that were not applicable (e.g., if the answers had images with graphs or drawings). A *p*-value of < 0.05 was considered significant. Since we are comparing three groups/chatbots, Bonferroni adjustment, Familywise adjustment for multiple measures, for multiple comparisons was applied. Regarding narrative coherence and errors, we calculated the overall correct answers as the relative proportion of correct answers provided among the overall test answers of each AI chatbot accuracy. A descriptive analysis of reasons for logical argumentation of correct answers and categorization of type error was reported by percentage in tables. Statistical analyses were performed with STATA/MP 16.1 software.

## Results

### AI chatbots’ multiple-choice questions

**Fig. 1:**
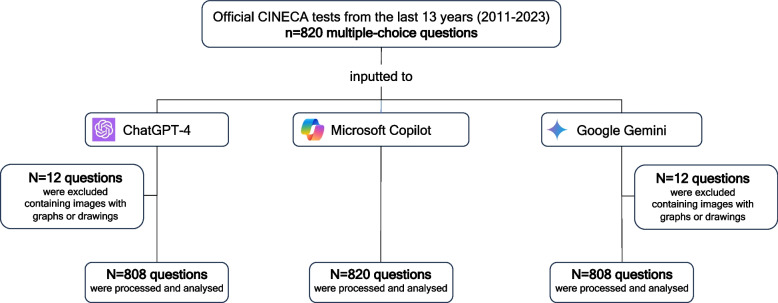
The study flow chart

From our original sample, we inputted all the multiple-choice questions in Microsoft Copilot (*n* = 820). Twelve multiple-choice questions were not imported in ChatGPT-4 (*n* = 808) and Google Gemini (*n* = 808) since they were images with graphs or drawings. The flowchart of the study is shown in Fig. 1.

### AI chatbots’ accuracy

Overall, we found a statistically significant difference in accuracy between the answers of the three chatbots (*p* < 0.001). The results of the Bonferroni adjustment, as a Familywise adjustment for multiple measures and tests between couples, are presented in Table 1. We found a statistically significant difference in the ChatGPT-4 vs Google Gemini (*p* < 0.001) and Microsoft Copilot vs Google Gemini (*p* < 0.001) comparisons, which indicate a better ChatGPT-4 and Microsoft Copilot accuracy than Google Gemini (Table 1). A sensitivity analysis excluding answers that were not applicable (e.g., if the answers had images with graphs or drawings) showed similar results reported in Additional file 4.

**Table 1:** AI chatbots’ accuracy

	**ChatGPT-4**		**Microsoft Copilot**		**Google Gemini**		**ChatGPT-4 vs Google Gemini**		**ChatGPT-4 vs Microsoft Copilot**		**Microsoft Bing vs Google Gemini**		**Overall among AI chatbots**	
**Failure**	Absolute frequ.	%	Absolute frequ.	%	Absolute frequ.	%	Chi2	*p*-value	Chi2	*p*-value	Chi2	*p*-value	Chi2	*p*-value
	57	6.96	83	10.13	246	30.04	-0.23	0.00*	-0.031	0.199	-0.198	0.00*	312.76	0.000*
***Logical reasoning and general culture***	39	68.42	51	61.45	126	51.22	-0.28	0.00*	-0.038	0.70	-0.242	0.00*	52	0.000*
***Biology***	6	10.53	8	9.64	31	12.60	-0.1	0.00*	-0.008	1.00	-0.09	0.00*	166.01	0.000*
***Chemistry***	7	12.28	11	13.25	32	13.01	-0.16	0.00*	-0.025	1.00	-0.13	0.00*	73.03	0.000*
***Physics and mathematics***	5	8.77	13	15.66	57	23.17	-0.43	0.00*	-0.066	0.46	-0.366	0.00*	94.16	0.000*

^*^ statistically significant findings

### AI chatbots’ narrative coherence: correct answers and errors

The Inter-rater agreement regarding AI chatbots’ narrative coherence was “almost perfect” ranging from 0.84–0.88 kappa for internal and logical answers (Additional file 5). The narrative coherence of AI chatbots is reported in Tables 2 and 3. We excluded from these analyses all not applicable answers (ChatGPT-4: *n* = 12, Microsoft Copilot: *n* = 0, Google Gemini: *n* = 12).

**Table 2:** Classification of AI chatbots’ correct answers

	**N not applicable**	**Logical reasoning** **N(%)**		**Internal information** **N(%)**		**External information** **N(%)**		**N tot valid answers**
***ChatGPT-4***	12	622	81.52	141	18.47	0	0	763
***Microsoft Copilot***	0	405	54.95	137	18.58	195	26.45	737
***Google Gemini***	12	567	98.78	7	1.21	0	0	574

*N* number, *% *percentage

**Table 3:** Classification of AI chatbots’ errors

	**N not applicable**	**Logical error** **N(%)**		**Information error** **N(%)**		**Statistical error** **N(%)**		**N total errors**
***ChatGPT-4***	12	40	88.98	4	4,50	1	22.22	88.88
***Microsoft Copilot***	0	66	79.01	9	11.39	8	70.23	79.01
***Google Gemini***	12	233	99.57	1	1.00	0	0	99.57

*N* number, *%* percentage

About the category of correct answer (Table 2), in ChatGPT-4 (tot = 763), the most frequent feature was “Logical reasoning” (*n* = 622, 81.5%) followed by “Internal information” (*n* = 141, 18.5%). In Microsoft Copilot (tot = 737), the main frequent feature was “Logical reasoning” (*n* = 405, 55%), followed by “External information” (*n* = 195, 26.4%) and “Internal information” (*n* = 137, 18.6%). In Google Gemini (tot = 574), the most frequent feature was “Logical reasoning” (*n* = 567, 98.8%), followed by a few cases of “Internal information” (*n* = 7, 1.2%).

With respect to category of errors (Table 3), in ChatGPT-4 (tot = 45), the main frequent reason was “Logical error” (*n* = 40, 88.9%), followed by a few cases of “Information error” (*n* = 4, 8.9%) and statistic (*n* = 1, 2.2%) errors. In Microsoft Copilot (tot = 83), the main frequent reason was “Logical error” (*n* = 66, 79.1%), followed by a few cases of “Information error” (*n* = 9, 11.1%) and “Statistical error” (*n* = 8, 9.8%) errors. In Google Gemini (tot = 234), the main frequent reason was “Logical error” (*n* = 233, 99.6%), followed by a few cases of “Information error” (*n* = 1, 0.4%).

## Discussion

### Main findings

The main findings reveal that: (A) AI chatbots reported an overall high accuracy in predicting the correct answer; (B) ChatGPT-4 and Microsoft Copilot performed better than Google Gemini; and (C) considering the narrative coherence of AI chatbots, the most prevalent modality to present correct and incorrect answers were “Logical” (“Logical reasoning” and “Logical error”, respectively).

Comparing our study with existing literature poses a challenge due to the limited number of research that have examined the accuracy of multiple AI chatbots [30–33]. Our research shows that AI chatbots can accurately answer questions from the CINECA Test, regardless of the topics (logical reasoning and general culture, biology, chemistry, physics and mathematics). This differs from the fluctuating accuracy found in other studies [34, 35]. Our findings support Torres-Zegarra et al.'s observations that the previous version of ChatGPT-4 and Microsoft Bing were superior to Google Bard [33], while other research groups did not confirm it [30–32]. This discrepancy may be due to differences in the tests used (e.g., medical specialties vs university entrance), the types of questions targeted at different stakeholders (e.g. professionals vs students), and the version of AI chatbots used (e.g., ChatGPT-3.5 vs 4).

The accuracy ranking of AI chatbots in our study might be due to differences in their neural network architecture. ChatGPT-4 and Microsoft Copilot AI use the GPT (Generative Pre-trained Transformer) architecture, while Google Gemini adopts LaMDA (Language Model for Dialogue Application) and later PaLM 2 (Pathways Language Model) in combination with web search [32]. The differences in the quality, variety, and quantity of data used for training, the optimization strategies adopted (e.g., fine-tuning), and the techniques applied to create the model could also account for the accuracy differences between AI chatbots [63]. Therefore, the variations mentioned above could lead to different responses to the same questions, affecting their overall accuracy.

In our study, the narrative coherence shows that AI chatbots mainly offer a broader perspective on the discussed topic using logical processes rather than just providing a simple answer [53]. This can be explained by the computational abilities of AI chatbots and their capacity to understand and analyze text by recognizing word connections and predicting future words in a sentence [63]. However, it is important to note that our findings are preliminary, and more research is needed to investigate how narrative coherence changes with advancements in AI chatbot technology and updates.

### Implications and future perspective

Our study identifies two contrasting implications of using AI chatbots in education. The positive implication regards AI chatbots as a valuable resource, while the negative implication perceives them as a potential threat. First, our study sheds light on the potential role of AI chatbots as supportive tools to assist candidates in preparation for the Italian entrance university standardized examination test of healthcare science. They can complement the traditional learning methods such as textbooks or in-person courses [10]. AI chatbots can facilitate self-directed learning, provide explanations and insights on the topics studied, select and filter materials and can be personalized to meet the needs of individual students [10]. In addition to the knowledge components, these instruments contribute to developing competencies, as defined by the World Health Organization [64]. Virtual simulation scenarios could facilitate the development of targeted skills and attitudes where students have a virtual interlocutor with a dynamic and human-like approach driven by AI. However, we should highlight that they cannot replace the value of reflection and discussion with peers and teachers, which are crucial for developing meta-competencies of today's students and tomorrow's healthcare professionals [10]. Conversely, candidates must be protected from simply attempting to use these tools to answer questions while administering exams. Encouraging honesty by avoiding placing and using devices (e.g., mobile phones, tablets) in classrooms is important. Candidates must be encouraged to respond with their preparation and knowledge, given that they are mostly applying for professions where honesty and ethical principles are imperative.

### Strengths and limitations

As a strength, we evaluated the comparative accuracy of three AI chatbots in the Italian health sciences university admissions test over the past 13 years on a large sample of questions, considering the narrative consistency of their responses. This enriches the international debate on this topic and provides valuable insights into the strengths and limitations of AI chatbots in the context of university education [2, 3, 8, 9, 11].

However, limitations exist and offer opportunities for future study. Firstly, we only used the CINECA Test, while other universities in Italy adopted different tests (e.g., CASPUR and SELECTA). Secondly, we studied three AI Chatbots without considering others presented in the market (e.g., Cloude, Perplexity) [31]. Thirdly, we adopted both paid (ChatGPT-4) and free (Microsoft Copilot and Google Gemini) versions of AI Chatbots. Although this choice may be a limitation, we aimed to use the most up-to-date and recent versions of the AI Chatbots available when the study was performed. Fourthly, although we inputted all queries into AI chatbots, we processed only some of them as only Microsoft Copilot was able to analyse complex images, as reported in the CINECA Tests, at the time of our study [65–67]. Fifthly, we inputted the test questions only once to simulate the test execution conditions in real educational contexts [32], although previous studies have prompted the test questions multiple times in AI chatbots to obtain better results [68]. However, an AI language model operates differently from regular, deterministic software. These models are probabilistic in nature, forming responses by estimating the probability of the next word according to statistical patterns in their training data [69]. Consequently, posing the same question twice may not always yield identical answers. Sixthly, we did not calculate the response time of the AI chatbots since this variable is affected by the speed of the internet connection and data traffic [51]. Seventhly, we assessed the accuracy of AI chatbots in a single country by prompting questions in Italian, which may limit the generalizability of our findings to other contexts and languages [70, 71]. Finally, we did not compare the responses of AI chatbots with those of human students since there is no national ranking for admission in Italy, and each university draws up its ranking on its own.

## Conclusion

AI chatbots have shown promising accuracy in quickly predicting correct answers, producing writing that is grammatically correct and coherent in a conversation for the Italian entrance university standardized examination test of healthcare science degrees. However, the study provides data regarding the overall performances of different AI Chatbots with regard to the standardized examinations provided in the last 13 years to all candidates willing to enter a healthcare science degree in Italy. Therefore, findings should be placed in the context of a research exercise and may support the current debate regarding the use of AI chatbots in the academic context. Further research is needed to explore the potential of AI chatbots in other educational contexts and to address their limitations as an innovative tool for education and test preparation.

### Supplementary Information

Supplementary Material 1.

Supplementary Material 2.

Supplementary Material 3.

Supplementary Material 4.

Supplementary Material 5.

## Data Availability

The datasets generated and/or analysed during the current study are available in the Open Science Framework (OSF) repository, https://osf.io/ue5wf/.

## Abbreviations

AI: Artificial intelligence
CI: Confidence interval
CINECA: Consorzio Interuniversitario per il Calcolo Automatico dell'Italia Nord Orientale
GPT: Generative pre-trained transformer
IQR: Interquartile range
LaMDA: Language model for dialogue application
PaLM 2: Pathways language model
STROBE: Strengthening of Reporting of Observational Studies in Epidemiology
