# Supplementary material for: Comparative accuracy of ChatGPT-4, Microsoft Copilot and Google Gemini in the Italian entrance test for healthcare sciences degrees: a cross-sectional study
Source: BMC Med Educ. 2024 Jun 26;24:694. doi: 10.1186/s12909-024-05630-9 (PMC11210096; doi:10.1186/s12909-024-05630-9)
Supplement: Supplementary file 1 — Supplementary Material 1. [file 12909_2024_5630_MOESM1_ESM.pdf]

PROVA DI AMMISSIONE AI CORSI DI LAUREA DELLE PROFESSIONI SANITARIE

Anno Accademico 2023/2024

Test di Competenze di lettura e conoscenze acquisite negli studi

1. ***"Dall'ambiente in cui è vissuto e vive l'allievo porta nella scuola un nucleo di parole che già possiede: ne intende in buona parte i sensi e spesso ne fa un uso appropriato. Come ogni insegnante sa, è un nucleo che può presentare qualche stranezza di conformazione: risultano note parole anche assai rare, rese per qualche tempo popolari da un gioco, una trasmissione tv o qualche altro evento; e, magari, parole basilari della nostra lingua risultano invece non bencomprese o non possedute nel loro uso, nella loro pronuncia, grammatica e sintassi. Il nostro Dizionario italiano di base vuole offrirsi come strumento utile a un apprendimento consolidato, progressivo e dinamico di nuclei sempre più estesi del vocabolario della nostra lingua.***
All'allievo che lo consulta sono offerte, nel consueto ordine alfabetico, anzitutto le parole fondamentali dell'italiano, contrassegnate da un circoletto. Sono le parole "grammaticali", come articoli, preposizioni, avverbi di quantità, tempo e luogo e parole come abbandonare, conto, fotografia, gridare, respingere, scopo… Tornando e ritornando su queste anzitutto, circa duemila, l'insegnante dovrà far sì che siano ben comprese nelle loro sfumature di senso e ben utilizzate nell'uso parlato e scritto. A nostro avviso, non c'è livello di scuola in cui questo lavorio può essere definitivamente messo da parte. Con i loro estesi e articolati significati queste parole sono il cuore del cuore della nostra lingua. Sono quelle in cui, alla fin fine, si rannodano e spiegano i significati di tutte le altre innumerevoli parole della nostra lingua: per più del novantacinque per cento i discorsi che pronunziamo, scriviamo, leggiamo sono tessuti con queste parole. Garantircene il pieno possesso significa avvicinarci sensibilmente a un controllo, elementare ma sicuro, dell'essenziale."
(Tullio De Mauro, Prefazione al Dizionario italiano di base, Torino, Paravia, 1998, p. I, con adattamenti)
Secondo l'autore del brano, quale tra questi deve essere un obiettivo di base dell'insegnamento scolastico?
   A) Assicurare la capacità di comprendere e usare appieno il vocabolario fondamentale
   B) Presentare tutte le parole del vocabolario fondamentale, dando per scontato che gli allievi non le conoscano
   C) Rendere note tutte le parole di uso raro
   D) Presentare solo i significati essenziali delle singole parole
   E) Dare per scontata la comprensione del vocabolario di base e concentrarsi sulle parole più difficili

2. ***"(…) una volta quando i nostri figli erano piccoli, io e Lily li portammo a Disneyland, dove vollero andare su una complicata giostra che simulava un volo nello spazio, e io mi accorsi troppo tardi che ci saremmo trovati sigillati in una capsula che veniva fatta ruotare in una centrifuga per creare l'effetto dell'assenza di gravità. Quando finalmente la corsa finì e saltammo fuori disorientati dalla minuscola cabina, fra il terrore di esserestato lì dentro e lo sforzo di aver dovuto fingere di divertirmi, scoppiai a piangere"***.
Da *Un'Odissea* di D. Mendelsohn, G. Einaudi editore
Di quale paura, scegliendone una tra le seguenti, sembra soffrire il protagonista?
   A) Claustrofobia
   B) Agorafobia
   C) Aracnofobia
   D) Rupofobia
   E) Amatofobia

3. ***"Se comprendere è impossibile, conoscere è necessario, perché ciò che è accaduto può ritornare, le coscienze possono nuovamente essere sedotte ed oscurate: anche le nostre. Per questo, meditare su quanto è avvenuto è un dovere di tutti"***

P. Levi, *Se questo è un uomo.*

A quale evento tragico della storia fa riferimento P. Levi?

A) Shoah
B) Progrom
C) Foibe
D) Gulag
E) Internamento

4. ***«Poteva essere una Caporetto per Allegri la seconda giornata di campionato contro il Genoa a Marassi: bianconeri sotto di due reti già al 7'»*.**

(Genoa-Juve, il film della partita: 2-4, La Stampa, 26 agosto 2017)

Il riferimento alla battaglia di Caporetto come esempio paradigmatico di sconfitta corrisponde a quale figura retorica?

A) Antonomasia
B) Metafora
C) Allegoria
D) Eufemismo
E) Perifrasi

Test di Ragionamento logico e problemi

5. **La frase "se faccio, capisco" è equivalente alla frase:**

A) è impossibile che io faccia e non capisca
B) se non faccio, non capisco
C) se capisco, significa che ho fatto
D) posso capire se e solo se faccio
E) nessuna delle altre risposte è corretta

6. **"In ogni corso di laurea c'è almeno uno studente che termina gli esami in anticipo sui tempi previsti". Se questa affermazione è falsa, allora è vero che:**

A) c'è almeno un corso di laurea in cui nessun studente termina gli esami in anticipo
B) in tutti i corsi di laurea nessun studente termina gli esami in anticipo
C) in ogni corso di laurea c'è almeno uno studente che non termina gli esami in anticipo
D) c'è almeno un corso di laurea in cui tutti gli studenti terminano gli esami in anticipo
E) in tutti i corsi di laurea qualche studente termina gli esami in anticipo

7. **Laura è sincera. Molti medici sono sinceri. Tutte le persone sincere sono oneste. Se le precedenti osservazioni sono vere, quale delle seguenti è necessariamente vera?**

A) Laura è onesta
B) Laura è un medico
C) Tutti i medici sono onesti
D) Una persona onesta è certamente sincera
E) Laura è un medico sincero

8. **Il treno A impiega 120 minuti a percorrere 119 Km, il treno B 172 minuti a percorrere 171 Km, il treno C 90 minuti a percorrere 89 Km, il treno D 100 minuti a percorrere 99 Km e il treno E 180 minuti a percorrere 179 Km. Quale treno è il più veloce?**

A) E
B) A
C) C
D) D
E) B

9. In un albergo, per ottimizzare il servizio di accesso ai piani tramite l'utilizzo di un ascensore, viene suggerito di prenotare le corse nella sola direzione desiderata, in ascesa o in discesa. Attualmente l'ascensore è in attesa al terzo piano, il signor X si trova al secondo piano e desidera scendere al primo piano, il signor Y si trova al piano terra e desidera salire al terzo piano. Ricevute le prenotazioni di X e Y, quale tra i seguenti tragitti dell'ascensore garantisce a X e Y l'esclusività del servizio?

A) L'ascensore scenderà al secondo piano e preleverà X, scenderà al primo piano e lascerà X, scenderà al piano terra e preleverà Y, salirà al terzo piano e lascerà Y
B) L'ascensore scenderà al piano terra e preleverà Y, salirà al secondo piano e preleverà X, scenderà al primo piano e lascerà X, salirà al terzo piano e lascerà Y
C) L'ascensore scenderà al piano terra e preleverà Y, salirà al secondo piano e preleverà X, salirà al terzo piano e lascerà Y, scenderà al primo piano e lascerà X
D) L'ascensore scenderà al secondo piano e preleverà X, scenderà al piano terra e preleverà Y, salirà al terzo piano e lascerà Y, scenderà al primo e lascerà X
E) L'ascensore scenderà al secondo piano e preleverà X, scenderà al piano terra e preleverà Y, salirà al primo piano e lascerà X, salirà al terzo piano e lascerà Y

Test di Biologia

10. Il glucosio viene spesso immagazzinato nelle piante sotto forma di:

A) amido
B) glicogeno
C) clorofilla
D) proteine
E) fruttosio

11. L'emofilia è un carattere recessivo legato al cromosoma X. Scegliere quale affermazione è vera.

A) Una femmina potrà essere malata se figlia di una madre portatrice e di un padre malato
B) Una femmina sarà sempre malata se figlia di una madre portatrice e di un padre portatore
C) Una femmina potrà essere malata solo se figlia di una madre sana e di un padre malato
D) Un maschio non sarà mai malato
E) Una femmina non potrà mai essere malata

12. Quale dei seguenti composti NON è un acido grasso?

A) Acido glutammico
B) Acido palmitico
C) Acido stearico
D) Acido oleico
E) Acido butirrico

13. Qual è la causa dell'anemia falciforme?

A) Una mutazione nella catena β dell'emoglobina
B) La carenza di ferro nell'alimentazione
C) Una drastica diminuzione dei globuli rossi
D) La diminuzione del numero e del volume medio delle piastrine
E) Una dieta vegetariana o vegana

14. **Quale delle seguenti affermazioni NON è una prova a sostegno dell'origine endosimbiontica dei mitocondri?**
    A) I mitocondri vengono duplicati dalla cellula subito prima della mitosi
    B) I mitocondri contengono molecole circolari di DNA
    C) I mitocondri contengono ribosomi simili a quelli dei batteri
    D) La membrana interna dei mitocondri ha una composizione diversa da quella che delimita gli organuli cellulari
    E) I mitocondri possiedono un genoma proprio

15. **In che modo gli inibitori di tipo competitivo riducono l'attività di un enzima?**
    A) Legandosi al sito attivo dell'enzima
    B) Modificando il pH
    C) Modificando la struttura primaria dell'enzima
    D) Inattivando il substrato
    E) Innalzando l'energia di attivazione

16. **Quale delle seguenti patologie è causata da una tossina batterica?**
    A) Botulismo
    B) Mononucleosi
    C) Malaria
    D) Rabbia
    E) Epatite B

17. **Da che cosa è costituito il materiale genetico dei retrovirus come l'HIV?**
    A) RNA a singolo filamento che viene usato come stampo per la sintesi di DNA
    B) RNA a doppio filamento che viene usato come stampo per la sintesi di mRNA
    C) RNA a singolo filamento che viene usato come mRNA
    D) DNA a doppio filamento che viene usato come stampo per la sintesi di mRNA
    E) DNA a singolo filamento che viene usato come stampo per la sintesi di DNA

18. **Qual è la conseguenza di un *crossing over* bilanciato?**
    A) I cromosomi contengono materiale genetico sia materno che paterno
    B) I cromosomi omologhi non contengono più gli stessi geni
    C) I cromatidi fratelli non sono più uniti dal centromero
    D) I cromatidi fratelli contengono sequenze nucleotidiche identiche
    E) I geni presenti sui cromosomi omologhi occupano loci differenti

19. **Osservando al microscopio un preparato istologico s'identificano alcune cellule contenenti 21 cromosomi, ciascuno formato da due cromatidi. Si può affermare che si stanno osservando:**
    A) cellule in meiosi
    B) cellule appartenenti a una specie vegetale
    C) artefatti dovuti alla preparazione del vetrino
    D) cellule di un individuo affetto dalla sindrome di Down
    E) cellule somatiche di topo

20. **Quale delle seguenti affermazioni riferite alla respirazione cellulare e alla fotosintesi che avvengono nelle cellule eucariotiche NON è corretta?**
    A) Entrambe utilizzano NADH come trasportatore di elettroni e protoni
    B) Entrambe richiedono la presenza di specifici organuli cellulari
    C) La respirazione cellulare avviene in tutti gli organismi, mentre la fotosintesi avviene solo negli organismi autotrofi che sfruttano la luce solare come fonte di energia
    D) Entrambe prevedono reazioni cicliche
    E) La fotosintesi è un processo anabolico, la respirazione cellulare è un processo catabolico

21. Durante un ciclo di Krebs:
- A) vengono liberate due molecole di CO2
- B) una molecola di piruvato e una di ossalacetato si legano per formare una molecola di citrato
- C) si produce una molecola di ATP che viene subito convertita in GTP
- D) tre molecole di NADH vengono ridotte a NAD^+
- E) una molecola di FAD viene ossidata a $FADH_2$

22. La traslocazione reciproca è una mutazione cromosomica che:
- A) causa uno scambio di frammenti tra cromosomi non omologhi
- B) causa uno scambio di frammenti tra il cromosoma di origine materna e quello di origine paterna di una stessa coppia di cromosomi
- C) provoca una variazione nell'orientamento di due frammenti all'interno dello stesso cromosoma
- D) è sempre responsabile di patologie gravi
- E) è sempre responsabile di patologie gravi nella progenie

23. Quale delle seguenti affermazioni è corretta?
- A) Nell'eredità poligenica due o più geni influenzano un singolo carattere fenotipico
- B) Nella trasmissione di caratteri codominanti non viene rispettata la legge dell'assortimento indipendente
- C) I caratteri codominanti esistono solo nel caso di poliallelia
- D) Nel caso di poliallelia un individuo possiede più di due alleli per lo stesso gene
- E) Nel caso di un gene a dominanza incompleta gli ibridi manifestano sempre il carattere recessivo

24. Che cos'è la citosina?
- A) Una base azotata che nella molecola del DNA si appaia con la guanina
- B) Una base azotata che contiene due anelli eterociclici
- C) Un amminoacido contenente zolfo
- D) Una proteina del citoscheletro
- E) Una base azotata assente nella molecola dell'RNA

25. La duplicazione del DNA è definita semiconservativa in quanto:
- A) ogni filamento della doppia elica funge da stampo per la sintesi di un nuovo filamento complementare
- B) ogni filamento della doppia elica funge da stampo per la sintesi di un nuovo filamento uguale per il 50% a quello originario
- C) solo il 50% di ciascuna molecola di DNA neosintetizzata è identico al filamento stampo
- D) delle due doppie eliche ottenute dalla duplicazione di una molecola di DNA una contiene i filamenti originari e l'altra i filamenti neo sintetizzati
- E) durante la duplicazione del DNA possono comparire mutazioni che modificano la sequenza nucleotidica originaria

26. Quale delle seguenti molecole può andare incontro al processo di splicing?
- A) Il trascritto primario di un gene eucariotico
- B) Una molecola di mRNA eucariotico maturo
- C) Un gene eucariotico
- D) Il DNA dei procarioti
- E) Una proteina

27. Che cos'è la filogenesi?

A) La storia evolutiva di una specie
B) La formazione del fuso mitotico
C) Una fase dello sviluppo embrionale
D) Il processo di duplicazione del DNA batterico
E) La produzione di miofibrille nelle fibre muscolari

28. Quale delle seguenti ossa craniche è un osso pari?

A) Parietale
B) Frontale
C) Occipitale
D) Sfenoide
E) Etmoide

29. Che cos'è il mesoderma?

A) Uno dei foglietti embrionali
B) Lo strato cellulare compreso tra epidermide e derma
C) Il tessuto fogliare specializzato nella fotosintesi
D) Una delle tre meningi
E) Lo strato interno dei vasi sanguigni

30. Quale delle seguenti affermazioni sull'ormone ossitocina NON è corretta?

A) È prodotta solo dagli individui di sesso femminile
B) È prodotta dall'ipotalamo
C) È rilasciata dalla neuroipofisi
D) Induce le contrazioni della muscolatura uterina durante il parto
E) Stimola l'eiezione del latte dalle ghiandole mammarie durante l'allattamento

31. Durante il processo di ricombinazione genica quale enzima unisce i frammenti di DNA?

A) Ligasi
B) Polimerasi
C) Primasi
D) Elicasi
E) Nucleasi

32. Quale tecnica della biologia molecolare richiede l'uso del termociclatore?

A) PCR
B) Elettroforesi del DNA
C) Elettroforesi delle proteine
D) Southern blotting
E) Denaturazione delle proteine

Test di Chimica

33. Qual è l'unità di misura utilizzata nel sistema internazionale (SI) per esprimere la quantità di sostanza?

A) La mole
B) Il chilogrammo
C) Il grammo
D) Il metro cubo
E) Il grammo al centimetro cubo

34. Quale dei seguenti composti NON contiene azoto?
- A) Glicerolo
- B) Metilammina
- C) Cianuro di potassio
- D) Guanina
- E) Metionina

35. Nelle stesse condizioni di pressione e temperatura:
- A) 1g di O2 occupa un volume minore di 1g di H2
- B) 1 mole di N_2 occupa un volume doppio di 1 mole di He
- C) 0,5 moli di H_2 pesano come 1 mole di He
- D) 1g di O_2 contiene lo stesso numero di molecole di 1g di N_2
- E) 1 mole di O_2 contiene lo stesso numero di atomi di 1 mole di CO_2

36. Quale delle seguenti affermazioni riferite ai gas NON è corretta?
- A) Tutti i gas sono incolori
- B) Tutti i gas hanno una bassa densità
- C) Esistono gas con molecole triatomiche
- D) Le particelle di un gas occupano in modo omogeneo il recipiente che le contiene
- E) I gas non possono liquefare per semplice compressione

37. Un catione trivalente appartiene all'elemento che ha Z=45 e A=103. Quanti sono gli elettroni presenti nello ione?
- A) 42
- B) 100
- C) 55
- D) 97
- E) 45

38. Che cosa determina l'appartenenza di un elemento ad un dato gruppo della tavola periodica?
- A) La configurazione elettronica esterna
- B) Il numero atomico
- C) Il numero quantico principale
- D) La massa atomica
- E) Le proprietà chimico-fisiche

39. Quale dei seguenti elementi chimici è un metallo alcalino-terroso?
- A) Calcio
- B) Cromo
- C) Stagno
- D) Piombo
- E) Alluminio

40. Quale delle seguenti affermazioni riferite ai legami chimici è corretta?
- A) Il legame tra due atomi con differenza di elettronegatività pari a 1,5 è di tipo covalente polare
- B) Il legame tra atomi dello stesso elemento è sempre di tipo covalente omopolare
- C) I legami tra gli atomi che formano una molecola sono detti intermolecolari
- D) Una molecola può contenere al massimo due legami di tipo covalente dativo
- E) Nel legame ionico l'elemento più elettronegativo cede elettroni all'elemento meno elettronegativo

41. Qual è la formula chimica del composto il cui nome tradizionale è anidride fosforosa?

A) P2O3
B) P_2O_5
C) F_2O
D) F_2O_3
E) PO_5

42. Quante moli d'idrossido di sodio sono necessarie per produrre 2 moli di fosfato di sodio secondo la reazione chimica da bilanciare:
aNaOH + $b$$H_3PO_4$ → $c$$Na_3PO_4$ +$d$$H_2O$?

A) 6
B) 5
C) 2
D) 1
E) 4

43. Qual è la concentrazione di una soluzione ottenuta mescolando 20mL di una soluzione 0,8M di HCl con 40mL di una soluzione 0,05M dello stesso acido?

A) 0,30M
B) 0,27M
C) 0,03M
D) 0,42M
E) 0,60M

44. Qual è il pH di una soluzione ottenuta aggiungendo 90 mL di acqua a 10 mL di una soluzione a pH 10?

A) 9
B) 9,9
C) 8,5
D) 8
E) 10

45. In una reazione di ossidoriduzione l'elemento riducente:

A) cede elettroni
B) ha il numero di ossidazione più alto
C) ha numero di ossidazione negativo
D) si trova in un solo composto reagente e in un solo prodotto
E) nei prodotti ha numero di ossidazione più basso che nei reagenti

46. Nella molecola di un alcol secondario:

A) l'atomo di carbonio che porta il gruppo alcolico lega un solo atomo d'idrogeno
B) l'atomo di carbonio che porta il gruppo alcolico lega due atomi d'idrogeno
C) sono presenti due gruppi alcolici
D) l'atomo di ossigeno del gruppo alcolico lega due atomi di carbonio
E) il gruppo alcolico è legato al carbonio con un doppio legame

47. Quanti atomi d'idrogeno sono presenti in una molecola di metilcicloesano?

A) 14
B) 12
C) 16
D) 15
E) 13

Test di Fisica e Matematica

48. Il fulcro di una leva (di estremi A e B) si trova in un punto distante i 2/5 della sua lunghezza dall'estremo A. Su A agisce perpendicolarmente alla leva una forza di modulo F_A (diretta verso il basso), su B una forza di modulo F_B (diretta verso il basso) anch'essa perpendicolare alla leva. Quando la leva è in equilibrio, quanto vale il rapporto F_A/F_B?

A) 3/2
B) 2/3
C) 5/2
D) 2
E) 2/5

49. Durante l'evaporazione a pressione costante di un liquido, la sua temperatura:

A) rimane costante
B) aumenta linearmente con il tempo
C) diminuisce linearmente con il tempo
D) aumenta linearmente con la massa evaporata
E) diminuisce linearmente con la massa evaporata

50. Un gas perfetto occupa un volume *V* a una pressione *P* ed è mantenuto a una temperatura costante. Se si varia la pressione raddoppiandola, cosa succede al volume?

A) Dimezza
B) Raddoppia
C) Resta inalterato
D) Quadruplica
E) Dipende dal valore della temperatura

51. Un'auto di massa 1,5 t e velocità di 100 km/h in modulo urta un furgone di massa 4 t e velocità di 80 km/h in modulo, procedendo entrambi nella stessa direzione e verso. Se subito dopo l'urto i due mezzi procedono insieme, come un tutt'uno, determinare ilmodulo della velocità con cui il sistema si muove:

A) circa 85 km/h
B) circa 70 km/h
C) circa 42 km/h
D) circa 31 km/h
E) circa 10 km/h

52. Si definisce portata di un fluido:

A) il volume di fluido che passa attraverso una data superficie in un secondo
B) la massa di fluido che attraversa una superficie unitaria
C) il prodotto della pressione per la velocità del fluido
D) il prodotto della pressione per il volume di fluido che passa attraverso una data superficie in un secondo
E) il prodotto della densità di fluido per la velocità di esso in un dato istante

53. Il primo principio della termodinamica esprime:

A) la conservazione dell'energia in presenza di fenomeni di variazione della temperatura
B) la conservazione dell'energia meccanica anche in presenza di forze non conservative
C) la definizione di equilibrio termodinamico
D) la trasformazione completa di quantità di calore in lavoro
E) la conservazione dell'entropia

54. Le soluzioni della disequazione $(x^2 + 4x)(x - 1) > 0$ sono:

A) $-4 < x < 0$ o $x > 1$
B) $0 < x < 1$ o $x > 4$
C) $x < -4$ o $0 < x < 1$
D) $x < 0$ o $1 < x < 4$
E) nessuna delle altre risposte è corretta

55. Qual è l'equazione della retta tangente alla parabola $y = 3x^2 - 4x + 1$ tracciata dal suo punto P di ascissa nulla?

A) $y = 1 - 4x$
B) $y = 3x - 4$
C) $y = 4x - 1$
D) $y = 3x + 1$
E) non esiste la retta tangente alla parabola tracciata dal punto P

56. Quale fra le seguenti è una circonferenza reale di centro (1, -1)?

A) 2x2 + 2y2 − 4x + 4y − 3 = 0
B) $x^2 + y^2 + 2x - 2y = 0$
C) $x^2 + y^2 - x + y - 2 = 0$
D) $x^2 + y^2 - 2x + 2y + 4 = 0$
E) $2x^2 + 2y^2 + 4x - 4y + 1 = 0$

57. Il polinomio $p(x) = x^4 - x^3 - 7x^2 + x + 6$ non è divisibile per:

A) $x - 6$
B) $x - 1$
C) $x + 2$
D) $x + 1$
E) $x - 3$

58. Il coefficiente angolare della retta $y = -2x + 3$ è

A) -2
B) 3
C) 2
D) 1
E) -3

59. La retta di equazione $y = x - 2$ e la circonferenza di equazione $x^2 + y^2 = 1$ sono:

A) esterne
B) secanti
C) tangenti in (1, 0)
D) tangenti in (2, 0)
E) tangenti in (0, 1)

60. La funzione $f(x) = |\ln x|$

A) è decrescente per $0 < x < 1$
B) è definita per $x < 0$
C) è decrescente per $x > 1$
D) è limitata
E) è pari

********** FINE DELLE DOMANDE **********

In tutti i quesiti proposti la soluzione è la risposta alla lettera A)

PROVA DI AMMISSIONE AI CORSI DI LAUREA DELLE PROFESSIONI SANITARIE

Anno Accademico 2022/2023

Test di Competenze di lettura e conoscenze acquisite negli studi

Leggere il brano e rispondere a ogni quesito solo in base alle informazioni contenute (esplicitamente o implicitamente) nel brano e non in base a quanto il candidato eventualmente conosca sull'argomento.

"Se non si fosse prodotto un tale fatto, noi non saremmo qui a parlarne." Nella descrizione dell'universo a varie riprese ci si imbatte in eventi, in apparenza fortuiti, che sembrano indispensabili alla comparsa dell'essere umano. Una concordanza del tutto straordinaria fra vari parametri nucleari ha reso possibile la nascita del carbonio nelle giganti rosse. Le popolazioni relative dei fotoni e dei nucleoni danno all'universo la longevità che si richiede per la comparsa della vita e al tempo stesso consentono la formazione delle stelle e delle galassie ecc. È molto lungo l'elenco di queste "coincidenze miracolose".
Il principio antropico è stato inventato, se non per spiegare, almeno per illuminare un po' questa situazione sorprendente. Esso può essere formulato press'a poco nel modo seguente: "dato che esiste un osservatore, l'universo ha le proprietà che si richiedono per generarlo". La cosmologia deve tener conto dell'esistenza del cosmologo. Queste domande non si sarebbero poste in un universo che non avesse avuto queste proprietà… Si obietterà a ragione che la materia possiede capacità di adattamento straordinarie. Uno sguardo ai pianeti vicini ci dimostra nondimeno che quest'adattabilità ha dei limiti. L'aridità della Luna e di Mercurio, le alte temperature di Venere hanno avuto ragione della frenesia vitale. Persino Marte, le cui condizioni non sono così dure, sembra sterile.

(Hubert Reeves, L'evoluzione cosmica, traduzione di Libero Sosio di Patience dans l'azur, Milano, Rizzoli, 1995, pp. 185-186)

1. In base alle informazioni fornite nel Brano 1, come può essere sintetizzato il principio antropico?

A) La comparsa prima della vita e poi degli esseri umani all'interno dell'universo è un processo inevitabile
B) Perché sia possibile a un essere vivente porsi domande sull'universo, l'universo deve essere fatto in modo da ospitare esseri viventi
C) Alcuni parametri nucleari hanno reso impossibile la comparsa della vita nell'universo
D) Ogni possibile universo deve avere caratteristiche fisiche tali da rendere possibile la comparsa della vita al suo interno
E) Se l'universo avesse caratteristiche tali da rendere impossibile la vita, al suo interno sarebbe comunque possibile a un osservatore interrogarsi sulla cosmologia

2. Nel contesto del Brano 1, per quale motivo l'autore presenta l'esempio di Marte?

A) Per far vedere in quali condizioni la vita potrebbe nascere facilmente, grazie alla sua adattabilità
B) Per chiarire le conseguenze della formazione delle stelle e delle galassie
C) Per mostrare come le proprietà dell'universo producano in altri contesti conseguenze diverse da quelle che hanno prodotto sulla Terra
D) Per mostrare che, per quanto la vita sia adattabile, bastano condizioni leggermente diverse da quelle della Terra per renderla impossibile
E) Per sintetizzare le condizioni dei pianeti più vicini alla Terra

3. Cosa regola il titolo II della prima parte della Costituzione Italiana?

A) I principi fondamentali
B) I rapporti economici
C) I rapporti politici
D) Il presidente della repubblica
E) I rapporti etico-sociali

4. Cosa si intende con l'aggettivo "divisivo"?

A) Che crea uno spettro distorto dell'orizzonte visibile ad occhio nudo
B) Che crea divisioni o contrapposizioni, impedendo di preservare o di raggiungere un'unità di punti di vista e di intenti
C) Che permette la conservazione del patrimonio ereditario famigliare
D) Che consente di appartare i convenuti secondo la loro volontà di separarsi e convergere verso un'unità di punti di vista
E) Che serve a distogliere dall'occupazione o preoccupazione dominante o a sviare l'argomento d'una conversazione

Test di Ragionamento logico e Problemi

5. Ieri il prezzo di un paio di scarpe era il 25% in più di oggi. Quindi oggi?
- A) è il 25% in meno di ieri
- B) è il 20% in più di ieri
- C) è il 25% in più di ieri
- D) è il 20% in meno di ieri
- E) è lo stesso di ieri

6. Ad un torneo di tennis singolo a eliminazione diretta sono iscritte 64 persone. Quante partite dovranno essere disputate per eleggere il campione?
- A) 64
- B) 128
- C) 63
- D) 127
- E) 32

7. In un gruppo di 42 persone ci sono 18 medici e 24 infermieri. Si vogliono scegliere 14 persone in modo da rispettare la proporzione tra medici e infermieri.
Quanti medici si devono scegliere?
- A) 8
- B) 7
- C) 5
- D) 6
- E) 9

8. Enea si prepara per la gara di triathlon. Enea si allena nel nuoto ogni tre giorni (intervallando ogni allenamento con due giorni di riposo), nella corsa a piedi ogni quattro giorni e nella corsa in bicicletta ogni cinque giorni.
Se Enea si allena anche di sabato e domenica e oggi si è allenato nel nuoto e nella corsa in bicicletta mentre ieri si è allenato nella corsa a piedi, fra quanti giorni (a partire da oggi) Enea si allenerà in tutte e tre le discipline?
- A) il 12° giorno
- B) il 15° giorno
- C) il 16° giorno
- D) il 18° giorno
- E) il 14° giorno

9. Le tavole di verità sono tabelle usate nella logica per determinare se, attribuiti i valori di verità alle proposizioni che la compongono, una determinata proposizione è vera o falsa.
Le tabelle di verità della congiunzione (∧), della implicazione (⇔) e della negazione non (¬), sono rispettivamente:

A	B	A ∧ B
V	V	V
V	F	F
F	V	F
F	F	F

A	B	A ⇔ B
V	V	V
V	F	F
F	V	F
F	F	V

A	¬A
V	F
F	V

Qual è la tabella di verità della proposizione: ¬ (A ⇔ B) ∧ (¬ A ⇔ (¬ B))?

A)

A	B	P
V	V	F
V	F	F
F	V	F
F	F	F

B)

A	B	P
V	V	F
V	F	V
F	V	F
F	F	V

C)

A	B	P
V	V	V
V	F	F
F	V	V
F	F	F

D)

A	B	P
V	V	F
V	F	F
F	V	V
F	F	V

E)

A	B	P
V	V	V
V	F	V
F	V	F
F	F	F

[illegible]

Test di Biologia

10. Gli organismi eterotrofi:
A) sono in grado di sintetizzare le proprie sostanze nutritive
B) acquisiscono energia partendo da sostanze organiche prodotte dagli autotrofi
C) si trovano solo tra i procarioti
D) sono tutti eucarioti
E) utilizzano l'energia solare per sintetizzare glucosio a partire da sostanze inorganiche

11. Gli esseri viventi:
A) sono tutti visibili ad occhio nudo
B) sono o vegetali o animali
C) sono tutti pluricellulari
D) si dividono sempre per mitosi
E) sono tutti composti da cellule

12. Le coppie di basi complementari che si ritrovano nella struttura a doppia elica del DNA sono:
A) sempre coppie di una purina con un'altra purina
B) sempre coppie di una pirimidina con un'altra pirimidina
C) timina-adenina, citosina-guanina
D) dimeri di pirimidine
E) desossiribonucleotidi-trifosfati

13. La membrana plasmatica si trova:
A) solo nelle cellule procariotiche
B) solo nelle cellule eucariotiche
C) solo nelle cellule animali
D) in tutte le cellule
E) in tutte le cellule meno quelle vegetali

14. Una anomalia in cui è presente un cromosoma in più o uno in meno rispetto al normale è detta:
A) trisomia
B) aneuploidia
C) monosomia
D) cariotipo
E) traslocazione

15. Nelle cellule che svolgono attività secretoria è particolarmente sviluppato:
A) il nucleo
B) l'apparato del Golgi
C) il rivestimento delle cellule
D) il numero dei lisosomi
E) il numero dei mitocondri

16. La comparsa di proprietà differenziate in una cellula eucariote è possibile grazie a:
- A) espressione selettiva di geni specifici
- B) ridondanza genica
- C) espressione sequenziale di tutti i geni
- D) delezione selettiva di geni specifici
- E) inibizione simultanea di tutti i geni

17. Le membrane biologiche sono semipermeabili. Ciò significa che il passaggio di sostanze attraverso di esse:
- A) avviene sempre con consumo d'energia
- B) è sempre mediato da canali
- C) è completamente libero dall'esterno verso l'interno
- D) è completamente libero dall'interno verso l'esterno
- E) non è completamente libero

18. Quale delle seguenti affermazioni non è corretta riguardo alla replicazione del DNA?
- A) La DNA elicasi separa i due filamenti del DNA
- B) Le proteine leganti il singolo filamento tengono separati i filamenti
- C) La DNA polimerasi richiede un primer e può sintetizzare un nuovo filamento di DNA soltanto in direzione 5’- 3’
- D) La replicazione comporta la formazione di frammenti di Okazaki sia sul filamento guida che su quello in ritardo
- E) La DNA topoisomerasi riduce l’avvolgimento del DNA davanti alla forca di replicazione

19. Le proteine che formano complessi regolativi con le chinasi che governano i processi che avvengono durante le fasi G1, S, G2, ed M del ciclo cellulare sono chiamate:
- A) Dineine
- B) Connessine
- C) Cicline
- D) Integrine
- E) Clatrine

20. La teoria endosimbiotica è proposta per spiegare:
- A) l'origine degli organelli membranosi intracellulari
- B) l'esistenza di un unico specifico precursore degli eucarioti
- C) che le cellule eucariotiche sono il prodotto dell'evoluzione dei procarioti
- D) l'origine dei mitocondri e dei cloroplasti
- E) che le cellule eucariotiche possono dare origine ad organismi multicellulari

21. La struttura primaria di una proteina:
- A) è la struttura tridimensionale della proteina
- B) è data dalla sequenza degli amminoacidi che la compongono
- C) è la struttura di alcuni segmenti della proteina
- D) non esiste
- E) è la struttura dei domini primari che la compongono

22. L'energia rilasciata durante il passaggio di elettroni nella catena di trasporto degli elettroni viene utilizzata per creare, ai due lati della membrana interna dei mitocondri, un gradiente di:
A) ADP
B) ATP
C) Ossigeno
D) Protoni
E) Elettroni

23. Quali tra i seguenti ioni o molecole passano per diffusione semplice attraverso il doppio strato lipidico della membrana plasmatica?
A) Ioni Sodio
B) Glucosio
C) Peptidi
D) Ioni Cloruro
E) Ossigeno

24. Gli anticorpi sono:
A) proteine di origine virale
B) proteine in grado di combinarsi con uno specifico antigene
C) cellule del sistema immunitario
D) proteine di origine batterica
E) geni in grado di combinarsi con uno specifico antigene

25. Un allele è:
A) la forma alternativa di una proteina presente nello stesso locus di cromatidi fratelli
B) la forma alternativa di un gene presente nello stesso locus di cromosomi omologhi
C) la duplicazione di un gene all'interno di un cromosoma
D) la forma alternativa di un gene presente su loci diversi all'interno dello stesso cromosoma
E) un'isoforma alternativa di un RNA messaggero

26. Se una tossina batterica distrugge i ribosomi, quale attività cellulare sarà influenzata per prima?
A) Sintesi proteica
B) Sintesi del DNA
C) Movimento
D) Stoccaggio di energia
E) Trasporto attivo

27. Gli enzimi sono catalizzatori biologici di natura:
A) monosaccaridica
B) polisaccaridica
C) steroidea
D) proteica
E) lipidica

28. Quale fra le seguenti non è una funzione del sistema linfatico?
A) Ritorno al sangue dei fluidi tissutali in eccesso
B) Trasporto dei grassi assorbiti dal sistema digerente
C) Difesa del corpo contro le malattie
D) Trasporto di proteine
E) Produzione dei globuli bianchi

29. Il sistema endocrino è in grado di:
- A) contribuire al mantenimento dell'equilibrio dei processi fisiologici
- B) modificare il metabolismo delle cellule
- C) inviare messaggi agli organi bersaglio
- D) tutte le altre risposte sono corrette
- E) utilizzare segnali chimici come sistema di comunicazione

30. Qual è il nome del processo mediante il quale le molecole fluiscono da un'area di maggiore concentrazione a una di minore concentrazione?
- A) Trasfusione
- B) Respirazione
- C) Diffusione
- D) Dialisi
- E) Trasporto attivo primario

31. Il capside di un virus consiste di:
- A) acidi nucleici
- B) subunità proteiche
- C) RNA e lipidi
- D) polisaccaridi
- E) lipidi elicoidali

32. Quali gruppi funzionali sono presenti in una molecola di glucosio nella sua forma lineare?
- A) 1 gruppo chetonico e 5 gruppi ossidrilici
- B) 1 gruppo chetonico e 4 gruppi ossidrilici
- C) 1 gruppo aldeidico e 5 gruppi ossidrilici
- D) 1 gruppo aldeidico, un gruppo chetonico e 4 gruppi ossidrilici
- E) 1 gruppo aldeidico e 6 gruppi ossidrilici

Test di Chimica

33. Quale è la natura del legame O-H nella molecola di acqua?
- A) Covalente puro
- B) Ad idrogeno
- C) Ionico
- D) Covalente polare
- E) Metallico

34. Un ossidante forte ha:
- A) una spiccata tendenza a donare elettroni
- B) una spiccata tendenza a ricevere protoni
- C) una spiccata tendenza a donare protoni
- D) nessuna delle altre risposte è corretta
- E) una spiccata tendenza a ricevere elettroni

35. Dove si trovano i neutroni?
- A) In orbite attorno al nucleo atomico
- B) Nei legami tra atomi
- C) Nel nucleo dell'atomo
- D) Negli atomi, come doppietti non condivisi
- E) Negli atomi paramagnetici come particelle spaiate

36. Nei cosiddetti "saponi" quali molecole organiche ci sono?
- A) Alcani
- B) Sali di acidi grassi a catena lunga
- C) Alcheni
- D) Alcoli
- E) Esteri

37. Negli ossiacidi a quale elemento è legato l'idrogeno acido?
- A) Al carbonio
- B) Al non-metallo
- C) Ad un altro atomo di idrogeno
- D) All'ossigeno
- E) All'azoto

**38. Si prelevi 1mL di una soluzione 1M di un qualsivoglia soluto.
Quanto vale la concentrazione di quel volume di soluzione prelevato?**
- A) 1 mM
- B) 1000 M
- C) 0.001 M
- D) 1 M
- E) Non è determinabile

39. La costante Kc di una reazione all'equilibrio:
A) varia solo modificando il volume del sistema
B) varia solo modificando la temperatura del sistema
C) in quanto costante non varia mai
D) varia solo modificando il numero di moli
E) varia solo modificando la pressione totale del sistema

40. La Kw dell'acqua in una soluzione 10^{-2} M di KCl a 25°C è:
A) 10^{-14}
B) 10^{-7}
C) 10^{-2}
D) 0
E) 10^{14}

41. Quanti elettroni sono contenuti nell'atomo di un elemento con configurazione elettronica [Ne] $3s^1$?
A) 11
B) 9
C) 4
D) 14
E) 8

42. Confrontando le soluzioni 0,1 M di fruttosio ($C_6H_{12}O_6$), 0,1 M di cloruro di potassio (KCl) e 0,1 M di cloruro di calcio ($CaCl_2$) a 25° C, si può affermare che:
A) le soluzioni hanno un pH differente
B) le soluzioni hanno la stessa tensione di vapore
C) la soluzione contenente cloruro di calcio è quella che bolle a temperatura inferiore
D) la soluzione contenente fruttosio è quella con la minore temperatura di congelamento
E) la soluzione contenente fruttosio è quella con la minore pressione osmotica

43. Alla fine di una reazione equimolare completa di neutralizzazione tra un acido forte monoprotico e una base forte monoprotica in soluzione avremo:
A) sale che da idrolisi acida
B) sale che da idrolisi neutra
C) sale che da idrolisi basica
D) sale che non da idrolisi
E) una soluzione tampone

44. La formula della legge di Henry per il calcolo della concentrazione di un gas in un liquido è:
A) PV = nRT
B) P = kT
C) C = kP
D) P = cRT
E) C = nkP

45. Se l'aggiunta di un sale provoca un innalzamento del pH, questo significa che:
A) il sale reagendo con l'acqua è andato incontro ad una idrolisi acida
B) il sale reagendo con l'acqua ha provocato un innalzamento della Kw
C) è avvenuta una reazione redox e dalla soluzione si sono sviluppati idrogeno e ossigeno gassosi
D) il sale reagendo con l'acqua è andato incontro ad una idrolisi basica
E) il sale era il prodotto di una reazione di neutralizzazione tra un acido forte ed una base forte

46. Quale è la formula generale per indicare i termini della serie degli alcani?

A) C_{2n} H_{2n+2}
B) C_n H_{2n-2}
C) C_n H_{2n}
D) C_{2n} H_n
E) C_n H_{2n+2}

47. Il composto che ha formula CH_3-CH_2-CHOH-CH_2-CH_3:

A) è un alcol terziario
B) è un estere
C) è un chetone
D) è un alcol secondario
E) è una aldeide

Test di Fisica e Matematica

48. In un sistema di riferimento inerziale, un punto materiale che si muove di moto circolare uniforme:
- A) è soggetto a una forza centrifuga
- B) è soggetto a una forza centripeta
- C) non è soggetto a forze
- D) è soggetto a forze con risultante nulla
- E) è soggetto a una forza di Coriolis

49. Sia F la forza di attrazione gravitazionale tra le masse m1 e m2 poste a distanza r. Quanto vale la forza di attrazione se si raddoppia ciascuna massa e si quadruplica la loro distanza?
- A) 4F
- B) F
- C) F/2
- D) F/4
- E) F/8

50. Un pendolo semplice di massa M = 2 Kg e lunghezza L = 9,8 m ha un periodo di oscillazione di:
- A) 1 s
- B) 4 s
- C) 4π s
- D) 2p s
- E) π s

51. Il momento di una forza F applicata nel punto A rispetto ad un punto O:
- A) è un vettore appartenente al piano generato da F e dal vettore che collega O ad A
- B) è un vettore il cui modulo dipende solo dal modulo della forza F
- C) è un vettore il cui modulo dipende esclusivamente dal modulo della forza F e dalla lunghezza del vettore OA
- D) non può mai essere nullo
- E) è un vettore perpendicolare al piano generato da F e dal vettore che collega O ad A

52. Quanto vale $201^2 – 199^2$?
- A) 4
- B) 400
- C) 2
- D) 200
- E) 800

53. Il prodotto $\sqrt{2} \cdot \sqrt[3]{4} \cdot \sqrt[4]{8}$ è uguale a:

A) $4\sqrt[12]{2^{11}}$

B) $2\sqrt[12]{2^{11}}$

C) 4

D) $2\sqrt[6]{2^5}$

E) $4\sqrt[6]{2^5}$

54. Il logaritmo in base 5 di 250 è uguale a:

A) 3
B) $3 + \log_5 2$
C) $3 + \log_2 5$
D) 5
E) $\log_{250} 5$

55. Il polinomio $27x^3 + 8$ si fattorizza come:

A) $(2x + 3)(4x^2 - 6x + 9)$
B) $(3x - 2)(9x^2 + 6x + 4)$
C) $(3x + 2)(9x^2 - 12x + 4)$
D) $(3x + 2)(9x^2 - 6x + 4)$
E) $(3x - 2)(9x^2 + 12x + 4)$

56. Le soluzioni della disequazione $x^2 - x - 6 < 0$ sono:

A) $x < -2$ o $x > 3$
B) $x < -3$ o $x > 2$
C) $-3 < x < 2$
D) nessuna delle altre risposte è corretta
E) $-2 < x < 3$

57. Quale di queste uguaglianze è corretta?

A) $\frac{\frac{1}{2}+\frac{1}{3}}{\frac{1}{5}} = \frac{1}{6}$

B) $\frac{\frac{1}{2}}{\frac{1}{5}} = \frac{1}{10}$

C) $\frac{1}{\frac{1}{2}+\frac{1}{3}} = \frac{6}{5}$

D) $\frac{\frac{1}{3}}{\frac{1}{5}} = \frac{3}{5}$

E) $\frac{3}{\frac{1}{2}+\frac{1}{3}} = \frac{5}{2}$

58. Quale delle seguenti rette è perpendicolare alla retta di equazione y = 2x + 3?

A) $y = \frac{1}{2}x + 3$
B) $y = -\frac{1}{2}x - 4$
C) $y = -2x + \frac{1}{3}$
D) $y = 2x + \frac{1}{3}$
E) Nessuna delle altre risposte è corretta

59. Il prodotto vettoriale di due vettori perpendicolari è:

A) il vettore nullo
B) un vettore con direzione e verso che segue la regola del parallelogramma
C) un vettore di modulo pari al prodotto dei moduli, con direzione perpendicolare ai due vettori e verso in accordo alla regola della mano destra
D) un vettore polare
E) uno scalare

60. Determinare da che altezza deve essere lasciato cadere un grave per raggiungere il suolo in 2s (si trascuri la resistenza dell'aria):

A) circa 19,6 m
B) circa 9,8 m
C) circa 40,2 m
D) circa 98,1 m
E) circa 125,1 m

PROVA DI AMMISSIONE AI CORSI DI LAUREA DELLE PROFESSIONI SANITARIE

Anno Accademico 2021/2022

Test di Ragionamento Logico e Cultura Generale

1. **Tommaso, Giorgio, Michele, Nicolò praticano ognuno una differente disciplina di snowboard e si allenano in quattro giorni differenti sulla stessa pista invernale. La pista è disponibile per gli allenamenti nei pomeriggi dei primi 4 giorni della settimana feriale. Ogni giorno della settimana è dedicato all'allenamento a turno di una delle discipline: half pipe, slope style, big air e bordercross. Sapendo che:**
   - **Giorgio si allena il lunedì o il martedì,**
   - **Nicolò che pratica big air, si allena il giovedì,**
   - **chi pratica il bordercross si allena il giorno dopo l'allenamento dello slope style e il giorno prima dell'allenamento di half pipe,**
   - **Michele, che si allena il giorno dopo Tommaso, pratica half pipe o slope style.**

   In base alle informazioni sopra riportate quale dei seguenti abbinamenti è corretto?
   A) Michele – half pipe – lunedì
   B) Giorgio – slope style – martedì
   C) Tommaso – bordercross – martedì
   D) Tommaso – half pipe – mercoledì
   E) Giorgio – bordercross – lunedì

2. **Quale tra i seguenti velivoli condivide una proprietà aggiuntiva al volo con tutte le parole 1, 2 e 3?**
   1. **mongolfiera**
   2. **aliante**
   3. **parapendio**

   A) Dirigibile
   B) Idrovolante
   C) Elicottero
   D) Jet
   E) Deltaplano

3. **Quale delle seguenti parole ha un nesso semantico sia con malato che con calmo?**
   A) Paziente
   B) Mansueto
   C) Sofferente
   D) Degente
   E) Indulgente

4. Siano p, q e r tre proposizioni, quale/i fra le seguenti proprietà è/sono vera/e?

P1 $(p \wedge q) \wedge r = p \wedge (q \wedge r)$

P2 $p \vee (q \wedge r) = (p \vee q) \wedge (p \vee r)$

P3 $q \wedge (q \vee r) = q$

A) Tutte
B) solo P2
C) solo P3
D) solo P1
E) Nessuna

5. Qual è la coppia anomala rispetto alle altre?

A) lavoro – opera
B) successo – vittoria
C) fallimento – trionfo
D) pizzo – merletto
E) gigante – ciclope

6. Qual è la proposizione contronominale della affermazione: "se il prossimo settembre il numero di contagi di corona virus aumenterà le scuole effettueranno lezioni a distanza e non in presenza"?

A) se il prossimo settembre il numero di contagi di corona virus non aumenterà le scuole effettueranno lezioni in presenza e non a distanza
B) se il prossimo settembre le scuole effettueranno lezioni in presenza e non a distanza allora il numero di contagi di corona virus non aumenterà
C) se il prossimo settembre le scuole effettueranno lezioni a distanza e non in presenza il numero di contagi di corona virus aumenterà
D) se il prossimo settembre il numero di contagi di corona virus non aumenterà le scuole effettueranno lezioni a distanza e non in presenza
E) se il prossimo settembre il numero di contagi di corona virus aumenterà le scuole effettueranno lezioni in presenza e non a distanza

7. Considerata la premessa: se Emma si allenerà duramente nelle prossime settimane, Alice correrà con Emma la staffetta dell'altipiano del Renon alla fine del mese di agosto. Consideriamo le quattro opzioni:

A – Alice non ha corso con Emma la staffetta dell'altipiano del Renon quindi Emma non si è allenata duramente nelle scorse settimane
B – Alice ha corso con Emma la staffetta dell'altipiano del Renon quindi Emma si è allenata duramente nelle scorse settimane
C – Emma non si allenata duramente nelle scorse settimane quindi Alice non ha corso con Emma la staffetta dell'altipiano del Renon
D – Emma si allenata duramente nelle scorse settimane quindi Alice ha corso con Emma la staffetta dell'altipiano del Renon.

Quale/i di queste, per la regola del modus ponens o del modus tollens, è/sono logicamentecorrette?

A) solo D
B) A e D
C) A, B e D
D) solo A
E) B e C

8. Enea, Michele, Nicolò e Tommaso hanno in tutto 53 fumetti. Se tutti possiedono almeno un fumetto e il numero dei fumetti di Enea è il triplo di quelli di Michele e il numero dei fumetti di Nicolò è il doppio di quelli di Tommaso aumentato di uno, qual è il massimo numero di fumetti che può possedere Nicolò?

A) 16
B) I dati assegnati non permettono di rispondere al quesito
C) 30
D) 33
E) 10

9. La profumeria di Enea propone per alcuni rossetti Lèvres in vendita al 50 % un ulteriore sconto del 25%. Emma, cliente della profumeria ha una tessera fedeltà che le garantisce uno sconto del 20 % su ogni acquisto presso la profumeria. Qual è lo sconto che ha ottiene Emma per l'acquisto di un rossetto Lèvres?

A) 95 %
B) 80 %
C) 72,5 %
D) 97,5 %
E) 70 %

10. Quale fra le seguenti affermazioni è la negazione della frase "tutti gli atleti che attualmente giocano negli All Blacks (nazionale di rugby neozelandese) sono nati in Nuova Zelanda"?

A) Tutti gli attuali All Blacks sono nati in Nuova Zelanda
B) Solo i nati in Nuova Zelanda giocano negli attuali All Blacks
C) Almeno un attuale All Blacks non è nato in nuova Zelanda
D) Nessun attuale All Blacks è nato in nuova Zelanda
E) Nessun nato in Nuova Zelanda gioca negli attuali All Blacks

11. Quale fra i seguenti è l'esatto ordine cronologico dei tre eventi elencati?

E1 – Giorgio De Chirico dipinge Le muse inquietanti
E2 – Roy Lichtenstein produce Crying Girl
E3 – Van Gogh dipinge Notte stellata

A) E2 – E1 – E3
B) E1 – E2 – E3
C) E3 – E2 – E1
D) E3 – E1 – E2
E) E2 – E3 – E1

12. Quali fra i seguenti è l'autore della novella *Il treno ha fischiato*?

A) G. Verga
B) G. D'Annunzio
C) G. Deledda
D) E. De Amicis
E) L. Pirandello

13. Quale termine può essere associato ad entrambe le seguenti definizioni?
"modo di vivere in relazione alle condizioni e possibilità economiche"
"la più acuta delle voci maschili"

A) Baritono
B) Livello
C) Tasso
D) Tenore
E) Basso

14. Con quale nome viene comunemente indicata la tecnica di conversione di dati da formato leggibile a un formato codificato che può essere letto o elaborato solo dopo che è stato decifrato?

A) Dattilografia
B) Aplografia
C) Crittografia
D) Stenografia
E) Calligrafia

15. In quale edificio romano si trovano le spoglie dei Re d'Italia Umberto I e Vittorio Emanuele II?

A) Altare della Patria
B) Ara Pacis
C) Basilica di Santa Maria in Aracoeli
D) Pantheon
E) Basilica di San Giovanni in Laterano

16. Come viene comunemente chiamata l'affezione psicosomatica che provoca tachicardia, capogiri, vertigini, confusione e allucinazioni in soggetti messi al cospetto di opere d'arte di straordinaria bellezza?

A) Sindrome di Stendhal
B) Sindrome di Stoccolma
C) Sindrome di Arts
D) Sindrome di Down
E) Sindrome di West

17. Quanti sono gli articoli della Costituzione Italiana riguardanti i principi fondamentali?

A) 12
B) 6
C) 10
D) 8
E) 18

18. Con quale simbolo viene indicato il coefficiente di resistenza aerodinamica usato per misurare la resistenza aerodinamica di un corpo in moto in un fluido?

A) A.D.C.
B) R_A
C) C.R.A.
D) A_{RC}
E) C_x

19. Quale fra i seguenti arcipelaghi si trova nell'oceano Atlantico?

A) Azzorre
B) Baleari
C) Cicladi
D) Egadi
E) Sporadi

20. Quale fra i seguenti è l'esatto ordine cronologico dei tre eventi storici elencati?

E1 – inizio rivoluzione di ottobre
E2 – fondazione partito fascista
E3 – fondazione partito nazionalsocialista tedesco

A) E3 – E2 – E1
B) E1 – E3 – E2
C) E2 – E1 – E3
D) E1 – E2 – E3
E) E3 – E1 – E2

21. Da quale città ebbe inizio il primo viaggio dell'Orient Express il famoso treno passeggeri che collegava XXX a Costantinopoli?

A) XXX = Londra
B) XXX = Parigi
C) XXX = Ginevra
D) XXX = Venezia
E) XXX = Vienna

22. Quale nome viene utilizzato per indicare i gruppi di processi ai nazisti coinvolti nella Seconda guerra mondiale e nella Shoah?

A) Processo di Norimberga
B) Processo di Berlino
C) Processo di Monaco
D) Processo di Mosca
E) Processo di Tokyo

Test di Biologia

23. Quale tra le seguenti NON è una caratteristica degli epiteli di rivestimento?
- A) Le cellule sono a stretto contatto tra loro
- B) Le lamine epiteliali hanno una superficie apicale libera
- C) La faccia inferiore poggia su una membrana basale
- D) Hanno una vascolarizzazione propria
- E) Possono essere costituiti da un solo strato di cellule

24. Quale tra le seguenti ossa del cranio è un osso pari?
- A) Frontale
- B) Occipitale
- C) Sfenoide
- D) Etmoide
- E) Parietale

25. Quale parte dell'encefalo coordina l'attività muscolare e l'equilibrio del corpo?
- A) Cervelletto
- B) Corpo calloso
- C) Diencefalo
- D) Midollo allungato
- E) Mesencefalo

26. L'ileo è:
- A) un osso formato dalla fusione delle vertebre sacrali
- B) la zona del rene attraversata da pelvi renale e vasi sanguigni
- C) la porzione interna degli alveoli polmonari
- D) l'ultimo segmento dell'intestino tenue
- E) il vaso linfatico all'interno dei villi intestinali

27. In condizioni fisiologiche, in un mm^3 di sangue, quali tra i seguenti elementi corpuscolati sono presenti in minore quantità?
- A) Granulociti basofili
- B) Granulociti neutrofili
- C) Monociti
- D) Piastrine
- E) Linfociti

28. Quale tra i seguenti composti NON è un amminoacido?
- A) Arginina
- B) Asparagina
- C) Istamina
- D) Serina
- E) Leucina

29. Quale tra le seguenti affermazioni relative a una patologia monogenica, autosomica recessiva è corretta?
A) È sempre trasmessa ai figli
B) Si manifesta solo allo stato di eterozigosi
C) Può essere trasmessa sia ai figli maschi che alle figlie femmine
D) Può essere trasmessa dalla madre solo ai figli maschi
E) La probabilità che due genitori portatori sani abbiano un figlio malato è del 50%

30. Il meccanismo con cui una cellula assume dall'ambiente sostanze liquide, inglobate in piccole vescicole, è chiamato:
A) endocitosi
B) fagocitosi
C) diffusione facilitata
D) pinocitosi
E) osmosi

31. La trasduzione è:
A) un'anomalia cromosomica
B) una fase della sintesi proteica
C) il meccanismo che permette di produrre proteine diverse partendo dallo stesso gene
D) lo spostamento di un trasposone all'interno del genoma
E) il trasferimento di geni da una cellula batterica ad un'altra tramite un fago

32. Il daltonismo è un carattere recessivo legato al cromosoma X. Qual è la probabilità che il figlio maschio di un uomo daltonico e di una donna con visione normale dei colori sia daltonico, sapendo che il nonno materno era daltonico?
A) 50%
B) 100%
C) 75%
D) 25%
E) 0%

33. La resistenza agli antibiotici che si sviluppa in alcuni ceppi batterici è comunemente determinata da:
A) proteine presenti nei plasmidi metabolici
B) operoni presenti nel cromosoma batterico, che vengono repressi in presenza dell'antibiotico
C) un capside che riveste la cellula batterica
D) vescicole citoplasmatiche contenenti enzimi idrolitici
E) geni presenti nei plasmidi R

34. La tecnica della PCR permette di:
A) isolare frammenti di DNA
B) amplificare frammenti di DNA
C) confrontare sequenze di DNA
D) inserire frammenti di DNA esogeno in una cellula
E) tradurre sequenze nucleotidiche in polipeptidi

35. Quale tra le seguenti affermazioni sugli anticorpi NON è corretta?
- A) Sono molecole proteiche
- B) Sono costituiti da quattro catene polipeptiche
- C) Sono secreti dai linfociti T
- D) Possono essere monomeri, dimeri, trimeri o pentameri
- E) Presentano regioni costanti e regioni variabili

36. Quale di questi processi consuma ATP?
- A) Ciclo di Krebs
- B) Fosforilazione ossidativa
- C) Ciclo di Calvin
- D) Trasformazione del piruvato in acido lattico
- E) Fase luminosa della fotosintesi

37. Il gene C presenta quattro varianti alleliche: tre sono codominanti (C^a, C^b, C^c) e una è recessiva (*c*). Quanti fenotipi differenti si possono ottenere dalla combinazione di questi alleli?
- A) 8
- B) 6
- C) 7
- D) 5
- E) 4

38. I lieviti, come il Saccharomices cerevisiae, sono:
- A) procarioti
- B) micoplasmi
- C) funghi
- D) vegetali
- E) protozoi

39. Quale delle seguenti proteine ha funzione enzimatica?
- A) Caseina
- B) Glucagone
- C) Emoglobina
- D) Pepsina
- E) Collagene

40. Quale tra i seguenti agenti, che causano le patologie specificate, è un virus?
- A) Variola major - Vaiolo
- B) Clostridium tetani - Tetano
- C) Yersinia pestis - Peste
- D) Vibrio colera - Colera
- E) Streptococcus pneumoniae – Polmonite

Test di Chimica

41. Gli elementi chimici conosciuti sono:
A) circa 80
B) circa 160
C) circa 120
D) circa 200
E) circa 240

42. La distillazione è un metodo che consente di separare due o più sostanze allo stato liquido con differente:
A) densità
B) peso specifico
C) solubilità
D) punto di ebollizione
E) massa molare

43. Quale dei seguenti elementi, dei quali è indicato il numero atomico Z, è un elemento di transizione?
A) Ca (Z=20)
B) Cl (Z=17)
C) Cs (Z=55)
D) C (Z=6)
E) Cu (Z=29)

44. Quali legami covalenti sono presenti tra l'atomo di cloro e gli atomi di ossigeno nella molecola $HClO_4$?
A) 2 semplici e 2 dativi
B) 3 semplici e 1 doppio
C) 2 semplici e 2 doppi
D) 1 semplice e 3 dativi
E) 1 semplice e 3 doppi

45. La variazione di quale dei seguenti fattori modifica il valore della costante di equilibrio K_c di una reazione?
A) Concentrazione dei reagenti
B) Presenza di catalizzatori
C) Temperatura
D) Stato di suddivisione dei reagenti
E) Velocità della reazione diretta

46. La relazione p/T = *k* rappresenta:
A) la legge isobara
B) la legge isoterma
C) la legge di Charles
D) la legge isocora
E) la legge di Boyle

47. Dalla disidratazione di un alcool si ottiene:

A) un alcano
B) un'aldeide
C) un chetone
D) un alchene
E) un etere

48. Le sostanze che disciolte in acqua liberano ioni sono dette:

A) ionizzanti
B) solubili
C) elettrofile
D) elettroforetiche
E) elettrolitiche

49. Quale dei seguenti è l'acido più debole?

A) H_2SO_4
B) H_2CO_3
C) H_3PO_4
D) HCl
E) HNO_3

50. Nella seguente reazione redox qual è la specie chimica riducente?
$Cr^{3+} + MnO_4^- + H_2O \rightarrow Cr_2O_7^{2-} + Mn^{2+} + H^+$

A) Cr^{3+}
B) MnO_4^-
C) $Cr_2O_7^{2-}$
D) Mn^{2+}
E) H^+

51. Per le infusioni endovenose si utilizza normalmente una soluzione di NaCl 0,9% *m/v*. La soluzione ottenuta:

A) ha un'osmolarità pari a 1,5 volte quella del plasma
B) contiene 9g di cloruro di sodio in 1L di soluzione
C) è ipotonica rispetto al plasma
D) ha un'osmolarità pari a 0,9 volte quella del plasma
E) contiene 0,9 moli di NaCl in 100mL di soluzione

52. "Tutte le soluzioni liquide di soluti non volatili hanno una pressione di vapore più bassa rispetto al solvente puro". Tale affermazione è riferibile alla legge formulata da:

A) W. Henry
B) T. Graham
C) F. M. Raoult
D) W.H. Nernst
E) G.H. Hess

Test di Fisica e Matematica

53. Qual è l'equazione cartesiana del luogo geometrico dei punti del piano assegnato dalle equazioni parametriche
$\begin{cases} x = t^2 \\ y = t^4 \end{cases}$ **x ∈ R?**

A) $y = x^4$, $x > 0$
B) $y = x^4$
C) $y = x^2$, $x \geq 0$
D) $y = 2x$, $x \geq 0$
E) $y = x^2$, $x > 0$

54. Considerati gli eventi A, B, C è noto che:
A, C sono incompatibili e B, C sono indipendenti, P(A) = 0,12, P(B) = 0,60.

Qual è la probabilità dell'evento C se $P(A \cup C) = P(B \cup C)$.
A) 0,8
B) 0,6
C) 0,2
D) 0,4
E) 0,88

55. Sia ABCD un trapezio rettangolo circoscritto ad una semicirconferenza avente per diametro l'altezza AD. Quale delle seguenti relazioni è vera?
A) BC ≅ 2AB
B) BC ≅ AB + AD
C) BC ≅ AB + CD
D) BC ≅ 2CD
E) BC ≅ CD + AD

56. Un astronauta compie una passeggiata spaziale a una distanza dalla superficie terrestre pari al triplo del raggio terrestre. A quale frazione di g corrisponde l'accelerazione subita dall'astronauta?
A) 1/4
B) 1/3
C) 1/16
D) 1/9
E) 1/8

57. Due sferette isolanti, rispettivamente di carica q e 2q e raggi r e 2r, vengono poste a contatto. Qualè la carica sulla sferetta di raggio r dopo il contatto?
A) q
B) 3q
C) 2q
D) 0,5q
E) 1,5 q

58. Due veicoli, inizialmente fermi nello stesso punto partono contemporaneamente, nella stessa direzione, con accelerazioni a e A (a < A), raggiungono in tempi diversi t e T la stessa velocità massima v per poi rallentare con decelerazioni d e D fino a fermarsi nello stesso istante. Indicando con s e S gli spazi percorsi dai due oggetti, quale delle seguenti relazioni è vera?

A) T < t
B) D = d
C) T = t
D) S = s
E) S > s

59. Nel moto di un proiettile lanciato da terra con velocità v_0= 10 m/s formante con un la verticale un angolo α= 30° qual è la velocità del proiettile nel punto di massima altezza della sua traiettoria?

A) Non calcolabile con i dati assegnati
B) 10
C) 8,660 m/s
D) 5 m/s
E) 0

60. L'energia interna di un gas ideale aumenta di 5 J in seguito ad una trasformazione adiabatica. Considerato positivo il lavoro eseguito dal gas, qual è il lavoro eseguito dal gas?

A) Non è possibile rispondere se non si specifica se il gas è monoatomico, biatomico o poliatomico
B) 0 J
C) Non è possibile rispondere se non si specifica il numero di moli del gas
D) 5 J
E) -5 J

PROVA DI AMMISSIONE AI CORSI DI LAUREA DELLE PROFESSIONI SANITARIE

Anno Accademico 2020/2021

Test di Ragionamento Logico e Cultura Generale

1. Qual è il tema affrontato nell'enciclica *Laudato sii* scritta da papa Francesco?

A) La fede
B) La fede e la ragione
C) Il lavoro e l'uomo
D) L'ecologia
E) La questione sociale

2. Quale termine corrisponde a entrambe le seguenti definizioni?
"sistema ottico centrato convergente"
"meta che si propone di raggiungere"

A) Traguardo
B) Microscopio
C) Bersaglio
D) Proiettore
E) Obiettivo

3. In quale giorno e mese da quest'anno viene celebrato il Dantedì?

A) 25 marzo
B) 14 settembre
C) 15 giugno
D) 27 gennaio
E) 15 agosto

4. In quale altro modo viene indicato il Meccanismo europeo di stabilità (MES)?

A) Fondo salva Stati
B) Next Generation
C) Recovery Fund
D) BTP Futura
E) Euro Bond

5. "La bandiera della Repubblica è il tricolore italiano: verde, bianco e rosso, a tre bande verticali di eguali dimensioni". La precedente affermazione dove viene sancita?

A) Da una legge del Regno d'Italia del 1861
B) Da una legge del Regno d'Italia del 1925
C) Da una legge della Repubblica Italiana del 1948
D) Da un articolo della Costituzione Italiana
E) Da una legge del Regno d'Italia del 1871

6. Questo anno causa Covid 19 si è avuta un'impennata di vendite dello strumento che misura l'ossigenazione del sangue. Con quale nome è noto tale strumento?
A) Viscosimetro
B) Glucometro
C) Saturimetro
D) Sfigmomanometro
E) Spettrometro

7. A quale secolo risale la riforma protestante attuata dal tedesco M. Lutero?
A) XV
B) XIV
C) XVII
D) XVI
E) XIII

8. Zeno Cosini è il nome del protagonista del romanzo La coscienza di Zeno. Chi è l'autore del romanzo?
A) Luigi Pirandello
B) Gabriele D'Annunzio
C) Italo Svevo
D) Giuseppe Ungaretti
E) Umberto Saba

9. Quale architetto ha firmato il progetto della ricostruzione del viadotto del Polcevera a Genova?
A) Stefano Boeri
B) Massimiliano Fuksas
C) Norman Foster
D) Mario Botta
E) Renzo Piano

10. Quale fra le seguenti parlamentari è stata la prima donna eletta Presidente del Senato della Repubblica Italiana?
A) Nilde Iotti
B) Emma Bonino
C) Maria Elisabetta Casellati
D) Laura Boldrini
E) Irene Pivetti

11. Chi realizzò il primo generatore statico di energia elettrica?
A) Luigi Galvani
B) Michael Faraday
C) Alessandro Volta
D) André Marie Ampere
E) Charles Augustin de Coulomb

12. Chi fra i seguenti è stato per 42 anni la massima autorità della Libia?
A) Mu'ammar Gheddafi
B) Ruhollah Khomeyni
C) Osama bin Laden
D) Saddam Houssein
E) Abu Bakr al-Baghdadi

13. Le tavole di verità sono tabelle usate nella logica per determinare se, attribuiti i valori di verità alle proposizioni che la compongono, una determinata proposizione è vera o falsa. Le tabelle di verità della disgiunzione (∨) della doppia implicazione (⇔), e della negazione non (¬) sono rispettivamente:

A	B	A ∨ B
V	V	V
V	F	V
F	V	V
F	F	F

A	B	A ⇔ B
V	V	V
V	F	F
F	V	F
F	F	V

A	¬A
V	F
F	V

Qual è la tabella di verità della proposizione ((←B ∨ A) ⇔ B) ∨ (←A)?

A	B	P
V	V	F
V	F	F
F	V	F
F	F	F

A	B	P
V	V	V
V	F	F
F	V	V
F	F	V

A	B	P
V	V	F
V	F	V
F	V	V
F	F	F

A	B	P
V	V	V
V	F	V
F	V	V
F	F	V

A	B	P
V	V	F
V	F	V
F	V	F
F	F	F

14. Alice ha avuto tre figli due dei quali sono gemelli. Se la somma delle età dei tre figli è di 45 anni e la differenza fra l'età del figlio maggiore e quella di uno dei figli minori (i gemelli) è 6, qual è l'età dei gemelli?

A) 16
B) 13
C) 19
D) 7
E) 11

15. L'azienda di Nicolò produce lavatrici. Il costo della produzione giornaliero di x lavatrici è, in euro, $C(x) = x^2 - 30x$, il prezzo di vendita è legato al numero di lavatrici vendute dalla funzione $R(x) = 800 + 40x$. Qual è il massimo numero di lavatrici che l'azienda può produrre giornalmente per non essere in perdita?

A) 35
B) 10
C) 140
D) 70
E) 80

16. Considerata la premessa: se Enea mangerà le carote a pranzo, Alice non potrà preparare la torta di carote come dessert per la cena. Consideriamo le quattro opzioni:

A) Enea non ha mangiato le carote a pranzo quindi Alice ha preparato la torta per la cena
B) Alice ha preparato la torta di carote per la cena per cui Enea non ha mangiato le carote a pranzo
C) Enea ha mangiato le carote a pranzo per cui alice non ha preparato la torta di carote per la cena
D) Alice non ha preparato la torta di carote per la cena quindi Enea ha mangiato le carote a pranzo

Quale/i di queste, per la regola del modus ponens o del modus tollens, è/sono logicamente corrette?

A) B e D
B) A, C e D
C) A e D
D) A, B e C
E) B e C

17. Giorgio acquista per il regalo di compleanno per Alice degli smalti per unghie di una famosa marca francese. Fortunatamente per Giorgio Alice compie gli anni nel mese di agosto e il grande magazzino in cui Giorgio deve effettuare l'acquisto vende gli smalti con uno sconto del 30%. Inoltre ad ogni cliente in possesso della tessera fedeltà del magazzino viene applicato un ulteriore sconto del 20% sul prezzo scontato degli articoli in vendita. Se Giorgio che possiede la tessera fedeltà, spende per l'acquisto del regalo per Alice 33,60 € qual è il prezzo di vendita, in euro, non scontato dello smalto?

A) 42
B) 67,2
C) 60
D) 76,4
E) 85

18. All'ultimo concerto dei BTS in uno stadio di Seul che ha una capienza di 66700 posti a sedere i posti sugli spalti rimasti vuoti erano i 2/5 dei posti totali. Michele e Tommaso che erano presenti all'evento assicurano che nel prato quelli che hanno assistito al concerto erano il 75% dei presenti sugli spalti. Quante persone erano presenti al concerto dei BTS?

A) 70035
B) 46690
C) 50025
D) 33350
E) 66700

19. Quale dei seguenti casi non è possibile se qualche X è Y?

A) ogni Y sia X
B) ogni X sia Y
C) qualche Y sia X
D) nessun Y sia X
E) ogni X sia Y e ogni Y sia X

20. Quale tra le parole A, B, C, D, E condivide una proprietà con tutte le parole 1, 2 e 3?

1) Araba
2) Berbere
3) Colono

A) Tettoia
B) Dipinti
C) Truffa
D) Addio
E) Oncologia

21. 90 studenti della scuola frequentata da Emma partecipano alla corsa campestre, di questi, fra cui Emma, il 40% è stato selezionato per la fase provinciale. Sapendo che i 4/9 sono ragazze, quante sono le ragazze selezionate per la fase provinciale oltre a Emma?

A) 20
B) 15
C) 19
D) 16
E) 18

22. Tommaso ha 3.90 € per poter acquistare della frutta. Le mele costano € 0,60 ciascuna, le banane € 0,80 le arance € 0,50 e le pere 0,75 €. Qual è il massimo numeri di frutti che Tommaso può avere acquistato?

A) 7
B) 6
C) 5
D) 9
E) 4

Test di Biologia

23. Quale tra le seguenti affermazioni relative agli α-amminoacidi NON è corretta?

A) Il gruppo laterale può essere aromatico
B) Il carbonio α lega un gruppo amminico e uno carbossilico
C) Il gruppo laterale può essere polare
D) Il gruppo laterale può essere elettricamente carico
E) Il carbonio α è sempre stereogenico

24. È corretto affermare che:

A) in un nucleotide lo zucchero e la base azotata sono uniti tra loro da un gruppo fosfato
B) le basi azotate presenti nella molecola di RNA sono adenina, guanina, timina e uracile
C) nella molecola di DNA i nucleotidi delle due semieliche si appaiano in modo complementare e sono uniti tra loro con 2 o 3 legami ionici
D) dalla traduzione di un filamento di DNA si ottiene una molecola di mRNA
E) si può ottenere una molecola di DNA partendo da uno stampo di RNA

25. Nelle cellule eucariotiche:

A) i mitocondri sono presenti solo nelle cellule animali e i cloroplasti solo in quelle vegetali
B) tutti gli organuli citoplasmatici sono delimitati da membrane tranne i lisosomi
C) la membrana nucleare è in continuità con il reticolo endoplasmatico ruvido
D) i componenti essenziali del citoscheletro sono: microfibre, microtubuli e microfilamenti
E) l'apparato di Golgi è la sede della sintesi dei lipidi

26. Nel metabolismo energetico delle cellule eucariotiche:

A) la fosforilazione ossidativa avviene sulla membrana esterna dei mitocondri
B) nelle prime reazioni della glicolisi (fase d'investimento energetico) si producono due molecole di ATP
C) la fosforilazione ossidativa produce la maggior parte dell'ATP di una cellula
D) la fermentazione lattica produce acido lattico e CO_2
E) in un ciclo di Krebs si producono tre molecole di $FADH_2$ e una di NADH

27. Una patologia congenita è sicuramente:

A) presente alla nascita
B) genetica
C) trasmessa da entrambi i genitori
D) trasmessa da un solo genitore
E) causata da una mutazione genica

28. Le membrane che rivestono la superficie interna di organi o cavità che comunicano con l'esterno sono dette membrane:

A) linfatiche
B) sierose
C) epiteliali
D) mucose
E) entoteliali

29. A differenza delle arterie, solo le vene possiedono:
A) tessuto connettivo
B) endotelio
C) muscolatura liscia
D) valvole
E) elastina

30. Quale delle seguenti vitamine regola l'assorbimento di calcio e fosforo?
A) Vitamina A
B) Vitamina D
C) Vitamina C
D) Vitamina E
E) Vitamina K

31. La capsula di Bowman:
A) riveste le ghiandole surrenali
B) avvolge il glomerulo renale
C) circonda l'oocita maturo
D) avvolge i neuroni sensoriali
E) contiene i canali semicircolari

32. Quale dei seguenti composti NON è un neurotrasmettitore?
A) Acetilcolina
B) Melanina
C) Serotonina
D) Glutammato
E) Dopamina

33. Quale delle seguenti affermazioni è corretta?
A) La clavicola si articola con la scapola
B) L'omero è l'unico osso dell'avambraccio
C) Tutte le coste sono collegate allo sterno
D) Cuboide, astragalo e scafoide sono ossa del carpo
E) Le vertebre libere sono: 5 cervicali, 12 toraciche e 7 lombari

34. Una malattia genetica, autosomica dominante:
A) può essere trasmessa solo ai figli maschi
B) è sempre trasmessa alla progenie
C) può essere trasmessa da un solo genitore
D) è sempre trasmessa dalla madre
E) si manifesta solo in condizioni di omozigosi

35. Quando nella porzione codificante di un gene si verifica una mutazione silente:
A) non si producono cambiamenti nella sequenza nucleotidica del gene
B) non si producono cambiamenti nella sequenza amminoacidica della proteina codificata
C) si modifica la sequenza amminoacidica della proteina codificata, ma senza effetti sulla sua funzionalità
D) il sequenziamento del DNA non è in grado di evidenziarla
E) un codone codificante è stato sostituito con uno di stop

36. La reazione a catena della polimerasi (PCR):
- A) può amplificare solo frammenti di DNA già completamente sequenziati
- B) necessita di nucleotidi modificati con aggiunta di fluorocromi
- C) prevede l'utilizzo di una DNA polimerasi batterica
- D) richiede una temperatura costante di 37° per tutta la durata del processo
- E) prevede l'utilizzo d'inneschi proteici (primer)

37. La pompa sodio-potassio:
- A) non necessita di ATP
- B) è presente solo nei neuroni motori
- C) agisce secondo gradiente di concentrazione
- D) trasporta ioni potassio verso l'esterno della cellula
- E) trasporta ioni sodio verso l'esterno della cellula

38. Il codice genetico è:
- A) degenerato
- B) l'insieme dei geni di un organismo
- C) uguale solo nei gemelli monozigoti
- D) differente nei procarioti e negli eucarioti
- E) ambiguo

39. NON è considerato organismo geneticamente modificato (OGM), in quanto non ottenuto mediante tecniche d'ingegneria genetica:
- A) il golden rice
- B) il mais Bt
- C) la soia resistente al glifosato
- D) il batterio che produce insulina
- E) la fragola ottoploide

40. Sia il D-glucosio che il D-fruttosio:
- A) contengono un gruppo aldeidico
- B) contengono un gruppo chetonico
- C) contengono sei atomi di carbonio
- D) nella forma ciclica hanno anelli a sei atomi
- E) sono dei disaccaridi

Test di Chimica

41. Quale tra le seguenti affermazioni è corretta?
- A) In acqua, il gruppo amminico primario si ionizza comportandosi da acido
- B) Il gruppo carbonilico dei chetoni è legato a un carbonio terminale
- C) Il gruppo carbossilico delle aldeidi è legato a un carbonio terminale
- D) Il gruppo ossidrile forma legami idrogeno con l'acqua
- E) Negli alogenuri alchilici un atomo di alogeno lega due atomi di carbonio

42. Quale tra le seguenti NON è una sostanza pura?
- A) Saccarosio
- B) Acciaio
- C) Cloruro di sodio
- D) Acqua ossigenata
- E) Stagno

43. Un composto con formula empirica C_2H_5O ha massa molare 135 g/mol. Qual è la sua formula molecolare?

A) $C_6H_{15}O_3$
B) CH_3CHOH
C) $C_4H_{10}O_2$
D) $C_8H_7O_2$
E) $C_6H_9 \cdot 3H_2O$

44. Dopo aver bilanciato la seguente reazione
$C_3H_8 + O_2 \rightarrow CO_2 + H_2O$
determinare qual è la resa massima di CO_2 che si può ottenere da 7,5 moli di O_2.

A) 7,5 moli
B) 5 moli
C) 2,5 moli
D) 15 moli
E) 4,5 moli

45. Qual è la formula del bicarbonato di ammonio, usato per la lievitazione dei dolci?

A) NH_4CO_3
B) NH_4HCO_3
C) $(NH_4)_2CO_3$
D) NH_3HCO_3
E) NH_3CO_3

46. Nella molecola dell'etilene C_2H_4 gli atomi di carbonio presentano ibridazione di tipo:

A) sp
B) sp^3
C) s^2p
D) sp^2
E) s^3p

47. Secondo la teoria di Brønsted–Lowry, quale tra le seguenti coppie di sostanze corrisponde a una coppia acido–base coniugata nella reazione $HCOOH + H_2O \rightleftharpoons H_3O^+ + HCOO^-$?

A) H_2O ; H_3O^+
B) $HCOOH$; H_2O
C) H_3O^+ ; $HCOO^-$
D) $HCOOH$; H_3O^+
E) H_2O ; $HCOO^-$

48. È possibile neutralizzare 25 mL di soluzione 0,04 M di KOH utilizzando:

A) 50 mL di soluzione di H_2SO_4 0,04M
B) 50 mL di soluzione di H_2SO_4 0,01M
C) 25 mL di soluzione di H_2SO_4 0,04M
D) 10 mL di soluzione di HCl 0,02M
E) 50 mL di soluzione di HCl 0,01M

49. Una reazione si definisce omogenea quando:

A) il numero delle moli complessive dei reagenti è uguale a quello delle moli complessive dei prodotti
B) il coefficiente stechiometrico di tutti i reagenti e di tutti i prodotti e pari a 1
C) il numero delle specie chimiche reagenti è uguale a quello delle specie chimiche prodotte
D) tutti i reagenti e i prodotti sono nella stessa fase
E) è all'equilibrio

50. Quale tra i seguenti abbinamenti tra il nome di un elemento chimico e il suo simbolo NON è corretto?

A) Bromo – B
B) Cesio – Cs
C) Cromo – Cr
D) Rame – Cu
E) Zolfo – S

51. Quale delle seguenti affermazioni NON è riferibile alle soluzioni colloidali?

A) Presentano l'effetto Tyndall
B) Presentano moto browniano
C) Le particelle disperse hanno dimensioni superiori a 10 µm
D) Le particelle disperse possono essere separate dal solvente mediante dialisi
E) Per aggiunta di elettroliti o per variazione di temperatura possono coagulare

52. Qual è il numero di ossidazione del cromo nello ione $Cr_2O_7^{2-}$?

A) +7
B) +6
C) +3
D) +2
E) −2

Test di Fisica e Matematica

53. Siano m ed n numeri interi relativi tali che m < 0 < n, quale delle seguenti affermazioni è falsa?

A) $m^3 < n^2$
B) $\sqrt[3]{m} < \sqrt[3]{n}$
C) $\frac{1}{m} > \frac{1}{n}$
D) $2^m < 2^n$
E) $-m > -n$

54. Assegnati i punti A (−3 , 2) e B (2 , 7) quali sono le coordinate del punto M che divide il segmento AB nel rapporto AM : MB = 3 : 2?

A) M (-2 , 3)
B) M (1 , 6)
C) M (0 , 5)
D) M (-1 , 4)
E) Non esiste nessun punto che soddisfa le richieste

55. Come è classificato il triangolo ABC se i suoi lati misurano 15 cm, 13 cm e 5 cm?

A) Il triangolo non esiste
B) Scaleno ottusangolo
C) Scaleno rettangolo
D) Scaleno acutangolo
E) Isoscele

56. Quale/i, fra le seguenti coppie di funzioni:

A: $y = 2\ln x$, $y = \ln x^2$; **B:** $y = \ln x$, $y = \ln(x^2 - x) - \ln(x-1)$; **C:** $y = 3\ln x$, $y = \ln^3 x$

è/sono composta/e da funzioni aventi lo stesso grafico?

A) Nessuna
B) Solo A
C) Solo B
D) Solo C
E) Tutte

57. Un'automobile si muove per 30 km in direzione E, successivamente si muove per $10\sqrt{10}$ km in direzione E α° N. Se $sen\alpha = \frac{3}{\sqrt{10}}$ qual è il modulo, in km, dello spostamento dell'automobile?

A) $30 + 10\sqrt{10}$
B) 70
C) $40\sqrt{10}$
D) 50
E) 10

58. Un punto si muove nel piano x y, le sue proiezioni sugli assi cartesiani seguono le leggi:

$$\begin{cases} x = 3t \\ y = 2t^2 \end{cases}$$

Quale delle seguenti affermazioni relative al moto del punto è vera?

A) Il moto è uniformemente accelerato con $v_0 = 3m/s$
B) Il moto è uniformemente accelerato con $a = 2m/s^2$
C) Il moto è uniformemente accelerato con $a = 5m/s^2$
D) Il moto è uniformemente accelerato con $v_0 = 5m/s$
E) Il moto è rettilineo uniformemente accelerato con legge oraria $s = 3t + 2t^2$

59. Un satellite artificiale della Terra ruota su un'orbita circolare di raggio R (misurato dal centro della Terra) con periodo T. Di quale fattore deve essere modificato il raggio dell'orbita affinché il satellite possa ruotare con periodo $\frac{T}{8}$?

A) 2R
B) R/2
C) 4R
D) R/8
E) R/4

60. **Due resistenze, rispettivamente di valore r e 2r, sono connesse in serie; quale valore R devono avere due resistenze uguali che, connesse in parallelo, forniscono una resistenza equivalente a quella prodotta dalle due resistenze in serie?**

A) 6r
B) 3r/3
C) 3r
D) r/6
E) r/3

********** FINE DELLE DOMANDE **********

PROVA DI AMMISSIONE AI CORSI DI LAUREA DELLE PROFESSIONI SANITARIE

Anno Accademico 2019/2020

Test di Ragionamento Logico e Cultura Generale

1. **"*Solamente dopo il lavaggio si può stirare la camicia*". Se la precedente informazione è corretta, quale delle seguenti affermazioni NON rappresenta una conclusione corretta?**
   A) Se la camicia è stata lavata allora può essere stirata
   B) Se la camicia è stata stirata allora è stata lavata
   C) Se la camicia non è stata lavata allora non può essere stirata
   D) Se la camicia non è stata stirata allora non è stata lavata
   E) È necessario lavare la camicia per poterla stirare

2. **"Non è errato non negare la non-esistenza del Destino". Quale tra le seguenti proposizioni ha significato equivalente alla precedente?**
   A) Risulta corretto negare l'esistenza del Destino
   B) Il Destino esiste
   C) Il libero arbitrio sarebbe limitato dall'esistenza del Destino
   D) Risulta errato negare l'esistenza del Destino
   E) Risulta corretto affermare l'esistenza del Destino

3. **Il tempo di reazione di Marco è di 3/4 di secondo. Conducendo una moto alla velocità di 104 km/h, Marco vede un ostacolo: quanti metri percorre prima di iniziare a frenare?**
   A) Circa 22
   B) Circa 78
   C) Circa 42
   D) Circa 18
   E) Non ci sono dati sufficienti per rispondere

4. **Quali tra i termini proposti completano correttamente la seguente proporzione?**
   Cera : X = latte : Y
   A) X = chiesa; Y = latteria
   B) X = ape; Y = lattaio
   C) X = incenso; Y = mucca
   D) X = candela; Y = burro
   E) X = pavimento; Y = torta

5. **11 amici escono a cena per festeggiare il compleanno di uno di loro. Quando il cameriere porta il conto, calcolano che ciascuno dovrebbe pagare 20 euro. Tuttavia decidono di non far pagare il festeggiato: quindi quanto dovrà spendere ciascuno degli altri amici?**
   A) 21 euro
   B) 22 euro
   C) 11 euro
   D) 23 euro
   E) 20 euro

6. **"*Non esiste rosa senza spine*". Se la precedente affermazione è FALSA, quale delle seguenti è necessariamente vera?**
   A) Nessuna delle altre alternative è corretta
   B) Tutte le rose hanno le spine
   C) Esiste almeno una rosa senza spine
   D) Alcune rose hanno le spine
   E) Nessuna rosa ha le spine

7. **"*Tutti i tau sono lambda; tutti gli omega sono tau; tutti i lambda sono delta*". In base alle precedenti affermazioni, quale delle seguenti NON è necessariamente vera?**
   A) I non lambda sono non delta
   B) Tutti gli omega sono lambda
   C) Tutti i tau sono delta
   D) I non delta sono non lambda
   E) I non tau sono non omega

8. **Quali tra i termini proposti completano correttamente la seguente proporzione?**
   Vespa : insetto = X : Y
   A) X = balena; Y = pesce
   B) X = pungiglione; Y = esoscheletro
   C) X = occhio; Y = pupilla
   D) X = mammifero; Y = leone
   E) X = lucertola; Y = rettile

9. **In un museo sono presenti soltanto quadri, sculture e mosaici. Sapendo che il numero di quadri sta a quello delle sculture come 2 sta a 3, che il numero di mosaici sta a quello dei quadri come 5 sta a 2 e che nel museo ci sono 114 sculture, qual è il numero totale di opere d'arte presenti nel museo?**
   A) 475
   B) 455
   C) 570
   D) 323
   E) 380

10. **Tre amici stanno passando le vacanze insieme. La mattina ognuno dei tre scende a un orario diverso per fare colazione. Il primo a fare colazione è sempre Riccardo. Una mattina sul tavolo ci sono cinque frutti, una mela, due albicocche, una banana e una pesca. Alberto e Luca mangiano solo albicocche e pesche. Riccardo invece mangia tutti i frutti. Si può dedurre con certezza che:**
    A) se Riccardo decide di mangiare due frutti presenti sul tavolino, uno degli altri due amici potrà mangiare due frutti rimanenti
    B) se Riccardo decide di mangiare due frutti presenti sul tavolino, entrambi gli altri due amici potranno mangiare uno dei frutti rimanenti a testa
    C) se Riccardo decide di mangiare due frutti presenti sul tavolino, almeno uno degli altri due amici potrà mangiare almeno uno dei frutti rimanenti
    D) se Luca scende per secondo e Riccardo ha mangiato due frutti, non potrà mangiare nessuno dei frutti rimanenti
    E) se Alberto scende per secondo e Riccardo ha mangiato due frutti, non potrà mangiare nessuno dei frutti rimanenti

11. Come si chiama il movimento letterario nato in Francia nella seconda metà dell'Ottocento sotto l'influsso del Positivismo, che prevede l'applicazione del metodo sperimentale al lavoro dello scrittore?

A) Naturalismo
B) Astrattismo
C) Classicismo
D) Esistenzialismo
E) Decadentismo

12. Quale dei seguenti eventi precede la prima guerra mondiale?

A) La conquista italiana della Libia
B) L'indipendenza dell'India dalla Gran Bretagna
C) L'ascesa al potere di Hitler
D) La nascita dello Stato di Israele
E) La nascita della Società delle Nazioni

13. Che cosa si intende per "flat tax"?

A) Un sistema fiscale non progressivo, basato su un'aliquota fissa
B) Una tassa molto bassa destinata ai soli ceti meno abbienti
C) Un sistema fiscale progressivo basato su tre sole aliquote, senza la possibilità di avere deduzioni o detrazioni fiscali
D) Un sistema fiscale basato su una tassa sui redditi "piatta", cioè vicina allo 0%, abbinata all'IVA al 25%
E) Una tassa "piatta" al 15% in sostituzione dell'IVA e dell'IRPEF, unita all'abolizione dell'IRAP

14. In Italia, quanto dura il mandato del Presidente della Repubblica?

A) Quattro anni
B) Cinque anni
C) Sei anni
D) Sette anni
E) Tre anni

15. Secondo la Costituzione italiana, la sovranità appartiene:

A) alla Nazione
B) al popolo
C) allo Stato
D) al Presidente della Repubblica
E) alle Camere

16. In quale periodo del XX secolo si colloca la Guerra fredda?

A) Negli anni Novanta
B) Tra la fine degli anni Quaranta e la fine degli Ottanta
C) Tra la fine degli anni Venti e la metà dei Cinquanta
D) Negli anni Trenta
E) A cavallo della seconda guerra mondiale

17. Quando e perché è scoppiato lo scandalo di Tangentopoli?

A) Negli anni '80, con la nascita dei primi governi di pentapartito, a causa della corruzione del mondo politico
B) Sul finire degli anni '70, con il venir meno della politica del compromesso storico
C) Nel 1992, in seguito alla scoperta di una fitta rete di corruzione tra mondo politico e mondo degli affari
D) Nel 1980, a causa della fitta rete di corruzione tra mondo politico e immobiliarismo dilagante
E) In occasione delle elezioni amministrative provinciali del 1991

18. Quale dei seguenti Stati è una Repubblica federale?

A) Germania
B) Spagna
C) Grecia
D) Egitto
E) Gran Bretagna

19. I primi esperimenti di genetica sui caratteri dominanti e recessivi delle piante furono condotti da:

A) Albert Einstein
B) Carlo Linneo
C) Renato Cartesio
D) Marie Curie
E) Gregor Mendel

20. Chi, tra i seguenti letterati, nacque per primo?

A) Giacomo Leopardi
B) Niccolò Machiavelli
C) Umberto Eco
D) Umberto Saba
E) Francesco Petrarca

21. Considerando l'avvenimento storico costituito dallo sbarco in Normandia. Quale delle seguenti affermazioni è esatta?

A) Avvenne il 6 giugno del 1914
B) Durante tale sbarco le truppe Francesi sbarcano in Normandia per liberare la Francia dall'occupazione tedesca
C) Durante tale sbarco Giulio Cesare di rientro dall'occupazione della Britannia libera la Gallia dalla ribellione delle popolazioni locali guidate da Vercingetorige
D) Avvenne il 6 giugno del 1944
E) Durante tale sbarco le truppe Statunitensi, Inglesi e Italiane sbarcano in Normandia valicando la linea Gotica per liberare la Francia dall'occupazione Tedesca

22. A settembre 2019, quale fra questi Paesi NON fa parte dell'Unione Europea?

A) Svizzera
B) Austria
C) Belgio
D) Danimarca
E) Croazia

Test di Biologia

23. In quale delle seguenti strutture cellulari si svolge la respirazione?
- A) Nel nucleo
- B) Nel reticolo endoplasmatico
- C) Nel mitocondrio
- D) Nella membrana cellulare
- E) Nel lisosoma

24. Le mutazioni sono:
- A) modificazioni casuali di proteine, non trasmissibili
- B) meccanismi con cui le cellule si difendono da eventi potenzialmente letali
- C) meccanismi di morte programmata delle cellule
- D) modificazioni casuali del materiale genetico, ereditabili dalle cellule figlie
- E) modificazioni del materiale genetico, non trasmissibili alle cellule figlie

25. Si immagini di avere una cellula contenente ribosomi, DNA, una membrana plasmatica, una parete cellulare e mitocondri. Che tipo di cellula potrebbe essere?
- A) Una cellula renale
- B) Un batterio
- C) Una cellula vegetale
- D) Una cellula uovo
- E) Un batteriofago

26. Quale delle seguenti affermazioni è vera?
- A) Il codone si trova nel tRNA, l'anticodone nel mRNA
- B) Il codone si trova nel DNA, l'anticodone nel tRNA
- C) Il codone si trova nel mRNA, l'anticodone nel DNA
- D) Il codone si trova nel mRNA, l'anticodone nel tRNA
- E) Il codone si trova nel DNA, l'anticodone nel mRNA

27. La membrana plasmatica:
- A) è formata da un doppio strato lipidico con proteine
- B) è formata da un solo strato lipidico
- C) nessuna delle altre alternative è corretta
- D) non è formata da alcuno strato lipidico
- E) è formata da un triplo strato lipidico

28. Il concetto biologico di specie si basa su:
- A) differenze anatomiche e di sviluppo tra due gruppi di organismi
- B) differenze genotipiche tra due organismi
- C) distribuzione geografica di due gruppi di organismi
- D) differenze nell'adattamento di due gruppi di individui
- E) isolamento riproduttivo di due gruppi di organismi

29. I batteri di forma sferica sono genericamente detti:
- A) spirochete
- B) bacilli
- C) vibrioni
- D) spirilli
- E) cocchi

30. Nel cuore, la circolazione sistemica ha origine:
A) dal ventricolo destro
B) dall'atrio sinistro
C) dal ventricolo sinistro
D) dall'atrio destro
E) dalla vena porta

31. Tra i seguenti organi, quale rappresenta negli esseri umani la prima difesa importante nei confronti dell'invasione da parte di virus e batteri?
A) La cute
B) La cistifellea
C) I reni
D) I muscoli scheletrici
E) Il cuore

32. Come viene denominato il processo biologico per cui gli RNA sono sintetizzati a partire da uno stampo di DNA complementare?
A) Trasduzione
B) Trascrizione
C) Sintesi proteica
D) Traslitterazione
E) Traduzione

33. Fa parte dell'apparato respiratorio:
A) l'esofago
B) la trachea
C) lo stomaco
D) il crasso
E) nessuna delle altre alternative è corretta

34. La struttura dell'occhio utile a mettere a fuoco gli oggetti è:
A) il cristallino
B) l'iride
C) la retina
D) i coni e i bastoncelli
E) nessuna delle altre alternative è corretta

35. Quale, tra le seguenti, è una funzione della bile?
A) Rompere la struttura quaternaria delle proteine
B) Scomporre i carboidrati in zuccheri semplici
C) Emulsionare i grassi
D) Favorire la motilità duodenale
E) Digerire le proteine

36. I virus sono:
A) i batteri patogeni
B) particelle infettive submicroscopiche costituite da proteine e acidi nucleici che si replicano esclusivamente all'interno delle cellule parassitate
C) i batteri non patogeni
D) particelle infettive submicroscopiche costituite da sole proteine e acidi nucleici che si replicano sia all'interno che all'esterno delle cellule
E) un sinonimo di prioni

37. Che cos'è una vitamina?

A) Una sostanza sintetizzata dall'organismo
B) Una sostanza di riserva
C) Un antibiotico
D) Una sostanza fondamentale per l'organismo introdotta dall'esterno
E) Nessuna delle altre alternative è corretta

38. Molti vaccini contro varie malattie sono molto spesso:

A) microrganismi la cui virulenza è stata attenuata
B) microrganismi la cui virulenza non è stata attenuata
C) anticorpi modificati
D) anticorpi non modificati
E) nessuna delle altre alternative è corretta

39. Quale delle seguenti malattie è ereditata come autosomica recessiva?

A) Fibrosi cistica
B) Malaria
C) Sindrome di Down
D) Morbo di Parkinson
E) Morbo di Basedow

40. Nella doppia elica del DNA:

A) la guanina è complementare alla timina
B) l'adenina è complementare all'uracile
C) le coppie di basi complementari sono formate da due purine o da due pirimidine
D) le basi azotate sono appaiate a caso
E) le coppie di basi complementari sono formate da una purina e una pirimidina

Test di Chimica

41. Nell'acqua, che è un solvente polare, si scioglie meglio:

A) NaCl (soluto ionico)
B) un grasso
C) la benzina
D) un sapone
E) nessuna delle altre alternative è corretta

42. 42. Il numero N = 6,022 · 10^{23}:

A) corrisponde alla costante dei gas perfetti
B) non esiste
C) corrisponde al numero quantico principale
D) è il numero di particelle contenute in una mole di sostanza
E) nessuna delle altre alternative è corretta

43. Per un gas ideale, se a temperatura costante:

A) si triplica il volume, la pressione diventa la terza parte
B) nessuna delle altre alternative è corretta
C) si aumenta il volume, la pressione resta costante
D) si dimezza la pressione, il volume si quadruplica
E) si raddoppia la pressione, si raddoppia anche il volume

44. Gli enzimi catalizzano le reazioni chimiche. Questo significa che:
- A) forniscono energia ai reagenti
- B) sottraggono energia ai reagenti
- C) le rendono energeticamente possibili
- D) aumentano la temperatura delle reazioni
- E) le accelerano, diminuendo l'energia di attivazione (E_a) di una reazione

45. Il composto KOH è:
- A) superossido di potassio
- B) perossido di potassio
- C) idrossido di potassio
- D) ossido acido di potassio
- E) idrossido di sodio

46. Indicare l'alogeno:
- A) Xe
- B) Ca
- C) I
- D) Fe
- E) nessuna delle altre alternative è corretta

47. Nel volume totale di un litro sono sciolte 0,1 moli di NH_3 e 0,1 moli di HCl. Sapendo che per NH_3 pKb = 4,75, la soluzione risultante avrà un pH:
- A) acido
- B) basico
- C) neutro
- D) uguale a 7
- E) superiore a 7

48. Si possono avere atomi di idrogeno quaternari in un alcano?
- A) Sì, sempre
- B) Sì, dipende dall'alcano considerato
- C) Sì, talvolta
- D) Sì, nel metano
- E) No, mai

49. Il legame chimico tra gli atomi di carbonio nell'etano è un legame di tipo:
- A) covalente polare
- B) covalente apolare
- C) ionico
- D) dativo
- E) metallico

50. La combustione completa di uno zucchero porta alla formazione di:
- A) un chetone e monossido di carbonio
- B) un acido e anidride carbonica
- C) anidride carbonica e acqua
- D) anidride carbonica e alcol
- E) acqua e alcol

51. La sublimazione è il processo per mezzo del quale una sostanza passa direttamente:
A) dallo stato solido allo stato liquido
B) dallo stato solido allo stato aeriforme
C) dallo stato liquido allo stato solido
D) dallo stato liquido allo stato aeriforme
E) dallo stato aeriforme allo stato solido

52. Alcuni elementi della tavola periodica presentano più isotopi, che differiscono tra loro per numero di:
A) protoni
B) elettroni
C) neutroni
D) bosoni
E) fermioni

Test di Fisica e Matematica

53. Ponendo in serie tre condensatori di capacità uguale a 30 microfarad ciascuno, la capacità totale vale:
A) 10 microfarad
B) 150 microfarad
C) 100 microfarad
D) 25 microfarad
E) 90 microfarad

54. Sapendo che l'accelerazione di gravità terrestre g ha valore 9,8 m/s^2, qual è la Forza esercitata dalla Terra su una bambina con massa di 20 Kg?
A) 19,6 N
B) 4,9 N
C) 490 N
D) 204 N
E) 196 N

55. Qual è l'altezza dalla quale cade un corpo dal peso di 2 N, sapendo che il lavoro compiuto dalla forza di gravità è pari a 2 J?
A) 2 m
B) 10 m
C) 20 m
D) 1 m
E) 0,5 m

56. Un conduttore ohmico ha una resistenza di 300 Ω ed è attraversato da una corrente di 80 mA. Quale sarà la tensione applicata ai capi del conduttore?
A) 24 V
B) 24.000 V
C) 3.750 V
D) 3,75 V
E) 0,25 V

57. 57. $\log_3(1/3 \cdot 3^3 \cdot 1/9)$ è uguale a:

A) 0
B) 3
C) –1
D) nessuna delle altre risposte è corretta
E) 1

58. Quali sono le soluzioni dell'equazione $4x^2 + 3x - 1 = 0$?

A) x = 1 e x = –1/4
B) x = 1/2 e x = 4
C) x = 1/3 e x = –4
D) x = 1/4 e x = –1
E) x = –3 e x = 3/2

59. Quale è l'ordine decrescente dei seguenti numeri?

$a = 3^{-10}$;
$b = 10/3$;
$c = -10^3$;
$d = -3^{10}$;
$e = -3/10$;
$f = 10^{-3}$

A) f, b, a, d, c, e
B) b, f, a, e, c, d
C) a, f, b, d, c, e
D) c, d, e, b, f, a
E) d, c, e, a, f, b

60. L'espressione $(a^5 + a^2b^3)$ è uguale a:

A) $a^2(a^2 + b^2)(a + b)$
B) $(a + b)^2(a^2 - ab + b^2)$
C) $a^2(a^2 - 2ab + b^2)$
D) $a^2(a + b)(a^2 - ab + b^2)$
E) $a^3(a^2 - ab + b^2)$

********** FINE DELLE DOMANDE **********

PROVA DI AMMISSIONE AI CORSI DI LAUREA DELLE PROFESSIONI SANITARIE

Anno Accademico 2018/2019

Test di Ragionamento logico

1. **Da "Chi dorme non piglia pesci" segue logicamente che:**
   A) chi non dorme piglia pesci
   B) chi non piglia pesci non dorme
   C) chi piglia pesci non dorme
   D) il sonno e la veglia sono del tutto irrilevanti sulla quantità dei pesci pescati
   E) chi non piglia pesci dorme

2. **A quante ore equivalgono 508 minuti?**
   A) 8h e 08'
   B) 7h e 38'
   C) 7h e 58'
   D) 8h e 28'
   E) 8h e 18'

3. **"L'esame dell'affresco non ha consentito di dimostrare l'impossibilità che l'autore sia il grande Michelangelo".**
   Qual è il corretto significato della precedente affermazione?
   A) Michelangelo potrebbe essere l'autore dell'affresco
   B) Michelangelo è l'autore dell'affresco
   C) È impossibile che l'autore dell'affresco non sia Michelangelo
   D) Michelangelo non può essere l'autore dell'affresco
   E) Michelangelo non è l'autore dell'affresco

4. ***"È sbagliato non ammettere che la competenza del medico non è bastata per impedire che la prognosi risultasse errata".* Basandosi sulla precedente affermazione, individuare quale delle seguenti alternative è esatta.**
   A) Bisogna ammettere che il medico non è stato competente
   B) Grazie alla competenza del medico la prognosi non è risultata errata
   C) Solo un medico competente può fare una diagnosi corretta
   D) La prognosi è risultata errata nonostante la competenza del medico
   E) La prognosi è risultata corretta nonostante l'incompetenza del medico

5. **Quali, tra i termini proposti, completano correttamente la seguente proporzione verbale?**
   Lindo : Pulito = X : Y
   A) X = Profumato; Y = Fresco
   B) X = Lucido; Y = Rugoso
   C) X = Casa; Y = Strada
   D) X = Acuto; Y = Intelligente
   E) X = Giusto; Y = Errato

6. Quale dei seguenti fatti (se fosse vero) costituirebbe un controesempio alla frase *"se piove, prendo l'ombrello"?*

A) Piove e non prendo l'ombrello
B) Non piove
C) Piove e prendo l'ombrello
D) Non piove e non prendo l'ombrello
E) C'è il sole

7. Una cassetta piena di arance pesa 70 chilogrammi. Sapendo che il peso delle arance rappresenta il 98% del peso lordo, qual è il peso della tara?

A) 0,7 chilogrammi
B) 1,4 chilogrammi
C) 2,8 chilogrammi
D) 68,6 chilogrammi
E) 7 chilogrammi

8. Se le lancette di un orologio segnano le 12.30 di lunedì, tra 45 ore e 45 minuti saranno:

A) le 6.15 di martedì
B) le 22.15 di giovedì
C) le 10.15 di mercoledì
D) le 10.15 di lunedì
E) le 9.15 di mercoledì

9. La prossima settimana Anna deve incontrare: Loredana, Roberta, Marisa, Paola, Gianni e Simona e ha a disposizione solo le sere di venerdì, sabato e domenica. Anna decide quindi di incontrare due amici ogni sera. Per organizzare gli appuntamenti si deve, però, ricordare che:

I) Roberta e Simona non vogliono incontrarsi tra di loro;
II) Paola non può uscire il venerdì sera;
III) Gianni può solo la domenica sera;
IV) Marisa e Loredana escono solo insieme.

In base alle precedenti affermazioni, per poter incontrare tutti e sei gli amici, quale delle seguenti persone incontrerà sicuramente il venerdì?

A) Roberta o Simona
B) Simona
C) Roberta
D) Paola
E) Loredana

10. Quali tra i termini proposti completano correttamente la seguente proporzione verbale?
GVIMSO : HUJLTN = X : Y

A) X = ZDIQRK; Y = ACJPSJ
B) X = ZDISRK; Y = ACJPSJ
C) X = ZDIQRK; Y = ACJPSK
D) X = ZDIPRK; Y = ACJPSJ
E) X = ZDIQRK; Y = ACJOSJ

11. Completetare la frase seguente scegliendo tra le alternative proposte.
"Condizione ... affinché un triangolo abbia i tre lati uguali, è che abbia i tre angoli uguali".
A) sufficiente ma non necessaria
B) necessaria ma non sufficiente
C) sufficiente
D) necessaria e sufficiente
E) necessaria

12. *"Maurizio ama la caccia. Chi abita in campagna ama la caccia. Giuseppe abita in campagna".*
Se le affermazioni precedenti sono vere, quale delle seguenti è necessariamente vera?
A) Chi ama la caccia abita in campagna
B) Maurizio abita in campagna
C) Chi non abita in campagna non ama la caccia
D) Giuseppe ama la caccia
E) Maurizio non abita in campagna

13. In una cantina il giorno 1 viene estratto un litro di vino da una damigiana. Il giorno 2 vengono estratti due litri di vino, uno da una damigiana e uno da un'altra. Il giorno 3 tre litri da tre damigiane, e così via, fino al giorno 19, quando, dopo l'estrazione, tutte le damigiane utilizzate, che sono tutte uguali ed erano tutte piene all'inizio del primo giorno, ora risultano vuote.
Qual è la capienza in litri di ciascuna delle damigiane presenti nella cantina?
A) 190
B) 19
C) 38
D) 20
E) 10

14. La ricetta per 12 muffin richiede 240 grammi di farina. Luigi decide di prepararne solo otto per una cena con amici.
Di quanti grammi di farina ha bisogno Luigi?
A) 220
B) 160
C) 180
D) 30
E) 200

15. Di ritorno dal lavoro, Franca percorre 15 km in automobile impiegando 40 minuti in tutto. Sapendo che per metà del tempo Franca è costretta a ridurre la velocità media di un terzo per via del traffico, qual è la sua velocità media nei tratti non trafficati?
A) 30 km/h
B) 33 km/h
C) 42 km/h
D) 50 km/h
E) 27 km/h

16. Posizionare le figure in ordine decrescente di superficie.

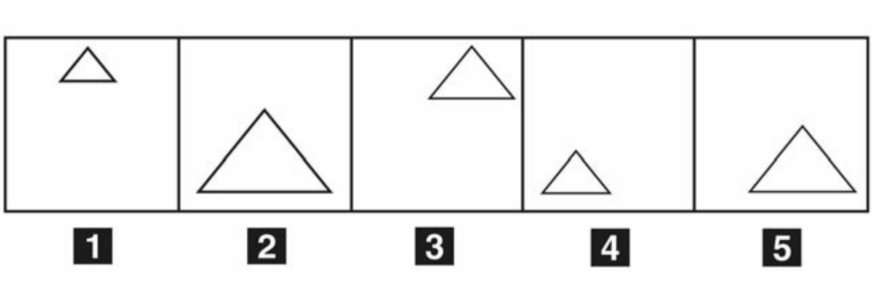

A) 1-3-4-5-2
B) 1-4-5-3-2
C) 1-4-3-2-5
D) 2-5-3-4-1
E) 2-1-4-3-5

Brano 1

Leggere il brano e rispondere a ogni quesito solo in base alle informazioni contenute (esplicitamente o implicitamente) nel brano e non in base a quanto il candidato eventualmente conosca sull'argomento.

Così tanto intelligenti che di più non si può: è questa la sintesi di una ricerca condotta dagli scienziati dell'Università di Cambridge secondo i quali il cervello umano avrebbe raggiunto il limite fisico del proprio sviluppo e delle proprie capacità. Secondo i ricercatori ci sarebbero insomma dei limiti biologici che impedirebbero all'uomo di diventare più intelligente di quanto lo sia adesso.

Gli scienziati inglesi hanno analizzato la struttura del cervello e hanno calcolato il suo fabbisogno energetico. «Abbiamo dimostrato che il cervello umano, per funzionare, consuma energia e questa evidenza da sola è sufficiente per affermare che esiste un limite alle sue performance» spiega Simon Laughlin, professore di neurobiologia presso l'ateneo britannico.

Una capacità deduttiva molto più sviluppata rispetto a quella attuale richiederebbe una quantità di energia significativamente maggiore perché il nostro cervello dovrebbe mettere in relazione tra loro informazioni provenienti da molte più fonti.

Altri studi sostengono che le connessioni tra le diverse aree del cervello non potrebbero essere più efficienti di quanto lo siano ora senza grandi investimenti in termini di energia. E proprio l'efficienza di queste connessioni sembra essere direttamente legata al quoziente intellettivo degli individui: chi ha la rete di comunicazione cerebrale più efficiente e veloce ha una maggior capacità di elaborazione e quindi è più intelligente.

Aumentare sensibilmente la "potenza di calcolo" del cervello richiederebbe insomma un aumento più che proporzionale nei consumi energetici.

«Prevedere il futuro è sempre rischioso, ma sembra evidente che le possibilità di sviluppo della nostra intelligenza siano fortemente limitate da fattori energetici», spiega Martijn van den Heuvel, docente di psichiatria presso l'Utrecht medical center, in Olanda.

(da: "www.focus.it”)

17. L'efficienza delle connessioni interne al cervello umano, secondo alcuni studiosi: (vedi Brano 1)

A) è connessa al quoziente intellettivo degli individui e per aumentare richiederebbe un grosso investimento di energia
B) non è connessa al quoziente intellettivo degli individui e per aumentare richiederebbe un grosso investimento di energia
C) è connessa al quoziente intellettivo, ma non alla capacità deduttiva degli individui
D) è connessa alla capacità deduttiva, ma non al quoziente intellettivo degli individui
E) è inversamente proporzionale al quoziente intellettivo degli individui

18. Secondo quanto riportato nel Brano 1, è più intelligente colui che:

A) riesce ad aumentare la propria potenza di calcolo
B) possiede connessioni cerebrali più attive e funzionanti
C) consuma neurologicamente più energia degli altri
D) dimostra negli studi citati un fabbisogno energetico minore del resto delle persone
E) dimostra di poter superare i limiti biologici propri dell'essere umano

19. Secondo una ricerca condotta dall'Università di Cambridge: (vedi *Brano 1*)

A) il cervello umano non ha raggiunto lo sviluppo massimo delle sue capacità, ma comunque non è plausibile un'ulteriore crescita dell'intelligenza
B) il cervello umano avrebbe raggiunto il limite di sviluppo delle sue capacità, a causa di limiti biologici che non consentono un'ulteriore crescita dell'intelligenza
C) il cervello umano con lo sviluppo delle sue capacità è causa di limiti biologici, che non consentono un'ulteriore crescita dell'intelligenza
D) il cervello umano, anche se ha raggiunto il limite di sviluppo delle sue capacità, può ancora contemplare un'ulteriore crescita dell'intelligenza
E) prevedere il futuro delle possibilità di sviluppo dell'intelligenza umana è sempre rischioso

20. Simon Laughlin: (vedi *Brano 1*)

A) ha ipotizzato che il cervello umano consumi energia per funzionare; questo comporterebbe un limite alle sue performance
B) ha dimostrato che il cervello umano consuma energia per funzionare, ma questo non implica un limite alle sue performance
C) ha ipotizzato che il cervello umano consumi energia per funzionare, analizzando la struttura del cervello e il suo fabbisogno energetico
D) ha dimostrato che il cervello umano consuma energia per funzionare, quindi ha un limite alle sue performance
E) ha calcolato il fabbisogno energetico del cervello umano, ovvero quanta energia sarebbe necessario consumare per sviluppare la massima performance potenziale

Test di Cultura generale

21. La data del 7 dicembre 1941 evoca:

A) l'inizio dell'operazione Barbarossa contro l'Unione Sovietica
B) l'attacco di Pearl Harbor
C) la dichiarazione di guerra degli Stati Uniti al Giappone
D) la dichiarazione di guerra di Hitler e Mussolini agli Stati Uniti
E) la battaglia delle Midway

22. Chi ha scritto l'*"Ulisse"*?

A) James Joyce
B) Vasco Pratolini
C) Alessandro Baricco
D) Alberto Moravia
E) Robert Musil

Test di Biologia

23. Un insieme integrato di cellule aventi la stessa funzione costituisce:
- A) un sistema
- B) un apparato
- C) un organo
- D) una molecola
- E) un tessuto

24. Le corde vocali sono situate:
- A) al di sotto dei bronchi
- B) in posizioni diverse a seconda della tonalità del suono che viene emesso
- C) nella trachea
- D) nella faringe
- E) nella laringe

25. I tRNA:
- A) sono responsabili della trascrizione del DNA
- B) sono RNA coinvolti nella terminazione della trascrizione
- C) trasportano i singoli amminoacidi ai ribosomi durante la sintesi proteica
- D) trasportano i singoli amminoacidi ai lisosomi durante la sintesi proteica
- E) sono RNA secreti nell'ambiente extra-cellulare

26. Quale struttura anatomica NON è presente in un essere umano di sesso maschile?
- A) Uretere
- B) Vescica
- C) Tuba di Falloppio
- D) Uretra
- E) Gonadi

27. La sindrome di Klinefelter è data da un’anomalia del numero dei cromosomi sessuali ed è caratterizzata da un genotipo:
- A) XXY
- B) XYY
- C) X0
- D) Y0
- E) XXX

28. In caso di faringite streptococcica, quale deve essere considerata la terapia d'elezione?
- A) La somministrazione di un corticosteroide
- B) La somministrazione esclusiva di un antipiretico
- C) L'idratazione per via endovenosa
- D) La somministrazione di penicillina
- E) La somministrazione di lassativi

29. La celiachia è un’allergia all'assunzione di alcuni alimenti caratterizzati dalla presenza di:
- A) amido resistente
- B) fibra alimentare
- C) lattosio
- D) proteine del glutine
- E) OGM

30. L'emoglobina è una molecola che si trova:
A) nel plasma
B) nei globuli rossi
C) nei globuli bianchi
D) nelle piastrine
E) nei vasi linfatici

31. L'informazione genetica è localizzata:
A) nei ribosomi
B) nessuna delle altre alternative è corretta
C) nei lisosomi
D) nel citoplasma
E) nei perossoni

32. In biologia, si definisce trascrizione la sintesi di:
A) DNA
B) RNA
C) polisaccaridi
D) lipidi
E) proteine

33. Quale delle seguenti affermazioni in merito alla traduzione è FALSA?
A) Durante la traduzione, una volta eseguito il suo compito, il tRNA, ormai privo dell'amminoacido che trasportava, viene rilasciato dal sito A del ribosoma
B) Il GTP rappresenta una fonte di energia durante i vari stadi della traduzione
C) La formazione del legame peptidico nel ribosoma è catalizzato dalla peptidil transferasi
D) Negli eucarioti la traduzione avviene prevalentemente nel citoplasma
E) 44 amminoacidi di un peptide sono codificati da 132 nucleotidi di un mRNA

34. Un carattere controllato da un gene mitocondriale si identifica, in quanto:
A) viene trasmesso sia dai maschi sia dalle femmine
B) viene trasmesso dalle femmine ai soli figli maschi
C) il maschio affetto non trasmette mai il carattere alla progenie
D) un figlio maschio non manifesta mai il carattere
E) una donna affetta trasmette il carattere alle sole figlie femmine

35. La vitamina C se assunta in grande quantità:
A) diminuisce le difese immunitarie
B) protegge dalle infezioni
C) nessuna delle altre alternative è corretta
D) provoca lo scorbuto
E) limita l'assorbimento del ferro

36. I gameti sono:
A) recettori cutanei
B) cellule nervose e gliali
C) cellule uovo e spermatozoi
D) cellule muscolari e satelliti
E) cellule epiteliali

37. Un muscolo scheletrico dei vertebrati:

A) risulta collegato alle ossa per mezzo di legamenti
B) può piegare ma non estendere gli arti
C) lo si trova nelle pareti dei vasi sanguigni e dell'apparato digerente
D) è quasi sempre collegato a entrambe le estremità dello stesso osso lungo
E) può consentire la rotazione di parti del corpo

38. Quale delle seguenti affermazioni riguardanti i mitocondri NON è corretta?

A) I mitocondri contengono molecole di DNA e ribosomi; il genoma mitocondriale viene ereditato per via paterna
B) Nel mitocondrio si svolge la respirazione cellulare in presenza di ossigeno
C) La membrana interna dei mitocondri è ripiegata in numerose creste e contiene gli enzimi della catena respiratoria
D) La principale funzione dei mitocondri è la produzione di energia
E) Secondo una teoria, la presenza dei mitocondri nelle cellule eucariotiche deriva da una simbiosi tra un'ancestrale cellula eucariotica e una cellula procariotica

39. Da quali caratteristiche dipende la virulenza di un virus?

A) Infettività e potenziale patogeno
B) Invasività e tossigenicità
C) Invasività, tossigenicità e potenziale patogeno
D) Infettività e tossigenicità
E) Invasività, infettività, potenziale patogeno

40. Il termine mughetto è comunemente usato per indicare:

A) un'infezione batterica responsabile dell'arrossamento dei genitali dei neonati
B) un altro modo per chiamare la micosi che causa il *"piede d'atleta"*
C) una micosi a carico della mucosa orale, generalmente causata da *Candida albicans*
D) un'infezione virale tipica della zona dei gomiti caratterizzata da arrossamenti e pelle squamosa
E) un'infezione batterica che rovina le unghie

Test di Chimica

41. Il fluoro è un:

A) lantanoide
B) metallo alcalino
C) alogeno
D) metallo alcalino-terroso
E) gas nobile

42. $FeO + Mn \rightarrow MnO + Fe$
rappresenta una reazione di:

A) dissociazione
B) condensazione
C) doppio scambio
D) ossidoriduzione
E) neutralizzazione

43. Il numero di Avogadro rappresenta il numero di molecole contenute:
- A) in una mole
- B) in un m^3
- C) in un cm^3
- D) in un kg
- E) in un dm^3

44. Quale delle seguenti definizioni del petrolio è corretta?
- A) È una miscela di idrocarburi
- B) È un liquido di odore gradevole
- C) È facilmente estraibile
- D) È il prodotto della decomposizione di alberi
- E) È una miscela di gas

45. Le proteine sono macromolecole i cui monomeri sono:
- A) acidi grassi
- B) lipidi
- C) nucleotidi
- D) amminoacidi
- E) glucidi

46. La formula semistrutturale CH_2=CH_2 è quella:
- A) del propilene
- B) dell'etilene
- C) dell'isobutilene
- D) del cicloesilene
- E) del ciclopentanofenantrene

47. Se a temperatura costante si dimezza il volume di un gas, la sua pressione:
- A) si dimezza
- B) si mantiene costante finché non varia la T
- C) aumenta ma non raddoppia
- D) diminuisce di un terzo
- E) si raddoppia

48. L'atomo di sodio e l'atomo di potassio:
- A) appartengono al secondo gruppo della tavola periodica
- B) possiedono lo stesso numero di protoni
- C) appartengono allo stesso periodo della tavola periodica
- D) possiedono lo stesso numero di elettroni nei rispettivi livelli esterni
- E) sono dei non metalli

49. Una molecola si dice idrofoba se essa:
- A) è polare
- B) è solubile in acqua
- C) possiede atomi di azoto
- D) è carica negativamente
- E) è insolubile in acqua

50. Se si fanno reagire un acido e una base monoprotici in quantità equimolecolari, la soluzione risultante:
A) dipende dalla forza relativa dell'acido e della base 1
B) sarà acida
C) sarà neutra
D) dipende dalla concentrazione dell'acido e della base
E) sarà basica

51. La chimica organica è la chimica:
A) dell'ossigeno
B) dell'azoto
C) del carbonio
D) dell'idrogeno
E) del calcio

52. Il cloro a pressione e a temperatura ambiente si trova soltanto sotto forma di:
A) solido
B) liquido e gas in equilibrio
C) gas
D) liquido
E) soluzione

Test di Fisica e Matematica

53. Ponendo in parallelo due condensatori di capacità uguale a 80 microfarad ciascuno, la capacità totale vale:
A) 40 microfarad
B) 80 microfarad
C) 400 microfarad
D) 64 microfarad
E) 160 microfarad

54. Quale dei seguenti enunciati corrisponde alla prima legge di Ohm?
A) L'intensità di corrente in un conduttore è proporzionale alla differenza di potenziale applicata ai suoi estremi
B) La resistenza di un conduttore è inversamente proporzionale alla temperatura assoluta
C) La potenza dissipata in un conduttore è proporzionale alla corrente
D) La corrente in un conduttore si annulla a 0 K
E) La resistenza di un conduttore dipende dalle sue caratteristiche geometriche

55. Con quale accelerazione si deve muovere un martello del peso di 1 Kg per sviluppare una forza di 2 N?
A) 4 m/s^2
B) 2 m/s^2
C) 1 m/s^2
D) 10 m/s^2
E) 6 m/s^2

56. L'energia cinetica di un'automobile di massa uguale a 1.000 Kg che viaggia alla velocità di 6 m/s è pari a:

A) 3.000 J
B) 18.000 J
C) 36.000 J
D) 6.000 J
E) 9.000 J

57. L'equazione $x – 4 (2 – x) = –33$ ha soluzione per x uguale a:

A) 5
B) 35
C) –3
D) 2
E) –5

58. Disporre in ordine crescente i seguenti numeri:
$a = 3^4$; $b = –(4^3)$; $c = –3^{–4}$; $d = 4^{–3}$

A) b < c < d < a
B) c < b < a < d
C) c < b < d < a
D) b < d < c < a
E) b < c < a < d

59. Semplificando l'espressione $(x^4 – x^2) / (x^2 – 1)$ si ottiene:

A) $x – 1$
B) x^2
C) $x^2 / (x + 1)$
D) $–x^3$
E) $2x + 1$

60. Se x è un qualsiasi numero positivo, allora $\log_{10}x + \log_{10}x^2$ è uguale a:

A) $2 \log_{10}x$
B) $\log_{10}(x + x^2)$
C) $3 \log_{10}x$
D) $\log_{10}(3x)$
E) nessuna delle altre alternative è corretta

********** FINE DELLE DOMANDE **********

PROVA DI AMMISSIONE AI CORSI DI LAUREA DELLE PROFESSIONI SANITARIE

Anno Accademico 2017/2018

Test di Ragionamento logico

1. ***"Tutti i delfini nuotano. Tutti i delfini sono mammiferi. Tutti i mammiferi vivono a lungo".***
Quale delle seguenti affermazioni può essere dedotta dalle precedenti?
   A) Tutti i mammiferi sono delfini
   B) I delfini vivono a lungo
   C) Se un mammifero nuota, è un delfino
   D) I delfini sono gli unici mammiferi che nuotano
   E) I delfini non vivono a lungo

2. ***"Senza l'invito non è possibile partecipare all'inaugurazione, a meno di essere amico dell'organizzatore o di essere un'autorità del settore; tutti gli ingegneri sono autorità del settore".***
Se la precedente affermazione è vera chi, tra i seguenti, NON è certo di potere partecipare all'inaugurazione?
   A) Un ingegnere senza invito
   B) Il migliore amico dell'organizzatore senza invito
   C) Il commercialista dell'organizzatore senza invito
   D) Un cugino dell'organizzatore con l'invito
   E) Un ingegnere con l'invito

3. **In base ai risultati di uno studio condotto l'anno scorso, il 10% dei cittadini italiani è biondo. Sono biondi l'11% delle donne e l'8% degli uomini.**
Quale delle seguenti affermazioni è quindi necessariamente vera?
   A) L'anno scorso il 90% delle cittadine italiane non era biondo
   B) Non è possibile stabilire se fossero più numerosi gli uomini non biondi oppure le donne italiane non bionde l'anno scorso
   C) Nessuna delle altre alternative è corretta
   D) L'anno scorso il 92% degli uomini italiani era castano
   E) In Italia l'anno scorso le donne erano più numerose degli uomini

4. **Morgana deve distribuire un bonus di produzione di 3.500 euro tra i suoi quattro dipendenti. Progetta di destinarne la metà a Olivier, un quarto a Getulio, un quinto a Lario e un decimo a Sasha. Così facendo:**
   A) le resterebbero 112,5 euro non distribuiti
   B) esaurirebbe il bonus, dividendolo tra i quattro dipendenti
   C) le resterebbero 175 euro non distribuiti
   D) eccederebbe il bonus complessivo di 112,5 euro
   E) eccederebbe il bonus complessivo di 175 euro

5. **Se le lancette di un orologio segnano le 16.45 di venerdì, tra 51 ore e 30 minuti saranno:**
   A) le 20.15 di domenica
   B) le 16.15 di mercoledì
   C) le 8.15 di lunedì
   D) le 20.15 di martedì
   E) le 19.15 di domenica

6. **Nel tentativo di guadagnare qualcosa in più per andare in vacanza, 4 amici hanno svolto vari lavoretti per i loro vicini. Prima di iniziare, hanno pattuito che tutti i soldi guadagnati sarebbero stati condivisi equamente tra di loro. Si sa che:**
   - **Marta ha guadagnato euro 66;**
   - **Belinda ha guadagnato euro 43;**
   - **Gioacchina ha guadagnato euro 68;**
   - **Emmanuelle ha sorpreso tutti gli altri guadagnando euro 119 e quindi deve dei soldi agli altri amici.**

   Quanto deve ricevere Belinda da Emmanuelle?
   A) Euro 31
   B) Euro 76
   C) Euro 45
   D) Euro 6
   E) Euro 8

7. **La frase: "È stata respinta la prova della negazione della certezza che i cattivi non si astengono dal manifestare avversione per i buoni":**
   A) significa che i cattivi non amano i buoni
   B) significa che i buoni amano i cattivi
   C) significa che i buoni non amano i cattivi
   D) significa che i cattivi amano i buoni
   E) è priva di significato

8. **Se gli organizzatori del concerto di Capodanno non si adegueranno alla nuova normativa europea sulla sicurezza, potrebbero incorrere in un'ingente sanzione amministrativa che finirebbe con il ridurre i fondi per il concerto e potrebbe risultare estremamente dannoso per le finanze degli organizzatori. Questi sono quindi costretti ad adeguarsi alla normativa sulla sicurezza per non mettere a rischio il futuro dell'evento. Quale delle seguenti affermazioni esprime il messaggio principale del brano precedente?**
   A) Nessuna delle altre alternative è corretta
   B) Se non si riesce a innalzare il livello di sicurezza vi è il rischio di incorrere in una sanzione amministrativa ingente
   C) Se le norme vengono seguite allora il concerto di Capodanno sarà salvo anche per l'anno prossimo
   D) La sicurezza durante il concerto di Capodanno ha raggiunto livelli pericolosamente bassi
   E) Se non si adegua alla normativa europea, il concerto di Capodanno sarà a serio rischio di annullamento

9. **Il piastrellista Angelino pavimenta una stanza con X piastrelle rettangolari di dimensioni 4 x 7 cm, il cui costo unitario è 2 euro. Il piastrellista Aureliano pavimenta una stanza della stessa dimensione ma usando piastrelle da 6,5 x 7,5 cm, dal costo unitario di 2,1 euro. Quale rivestimento è costato di meno?**
   A) Quello utilizzato dal piastrellista Angelino
   B) Quello utilizzato dal piastrellista Aureliano, che è costato meno della metà dell'altro
   C) Quello utilizzato dal piastrellista Aureliano
   D) Quello utilizzato dal piastrellista Angelino, che è costato meno della metà dell'altro
   E) Non è possibile determinarlo senza conoscere la superficie delle due stanze

10. Quali, tra i termini proposti, completano correttamente la seguente proporzione verbale?
Abbiente : ricco = X : Y
A) X = aereo Y = cielo
B) X = giusto Y = errato
C) X = volante Y = macchina
D) X = stravagante Y = bizzarro
E) X = ambiente Y = chiuso

11. Maria Luisa frequenta un Corso di Laurea articolato in 30 esami. Canonicamente, i voti degli esami sono interi e vanno da 18 a 30. Attualmente la sua media è di 27 e sa che se nei prossimi due esami prenderà gli stessi voti di Bruno la sua media scenderà a 26,8.
Che voti ha preso Bruno nei due esami in questione?
A) 20 e 20
B) 22 e 18
C) 30 e 24
D) 30 e 22
E) 21 e 19

12. Si dispone di una bilancia a due piatti con il braccio sinistro che misura il quadruplo del braccio destro. Se nel piatto sinistro vengono posti 10 pesi uguali fra loro, quanti pesi dello stesso tipo devono essere posizionati nel piatto destro affinché la bilancia risulti in equilibrio?
A) 40
B) Non è possibile equilibrare la bilancia con un numero intero di pesi
C) 20
D) 10
E) 30

13. *"Se prendo il treno in orario allora arriverò a Napoli entro l'orario di cena, ma solo se arriverò a Napoli entro l'orario di cena allora mangerò una pizza e un babà".*
In base alla precedente affermazione, è certamente vero che:
A) se non mangerò una pizza e un babà significa che non ho preso il treno in orario
B) se non arriverò a Napoli entro l'orario di cena significa che non mangerò una pizza e un babà e che non ho preso il treno in orario
C) se ho mangiato una pizza e un babà significa che ho preso il treno in orario
D) se non arriverò a Roma entro l'orario di cena significa che non mangerò una pizza e un babà e che non ho preso il treno in orario
E) se arriverò a Napoli entro l'orario di cena significa che mangerò una pizza e un babà e che ho preso il treno in orario

14. *"Non si può negare che non sia lecito vietare l'accesso alle spiagge libere".*
In base alla precedente affermazione, è certamente vero che:
A) è lecito vietare l'accesso alle spiagge libere
B) non è illecito negare l'accesso alle spiagge libere
C) le spiagge libere sono soggette a regolamentazione restrittiva
D) bisogna tutelare l'accesso a tutte le spiagge
E) le spiagge libere devono essere accessibili

15. Quali tra i termini proposti completano correttamente la seguente proporzione verbale?
ETGKQM : FSHJRL = X : Y

A) X = XBGQPI Y = YAHNQH
B) X = XBGOPI Y = YAHNQI
C) X = XBGNPI Y = YAHNQH
D) X = XBGOPI Y = YAHNQH
E) X = XBGOPI Y = YAHMQH

16. Disporre in ordine decrescente le cinque figure:

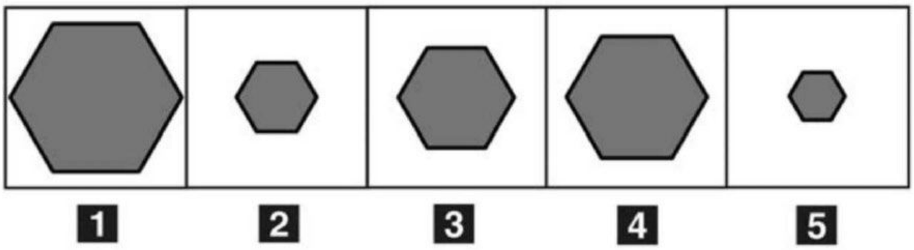

A) 1; 4; 3; 2; 5
B) 1; 3; 4; 2; 5
C) 1; 4; 3; 5; 2
D) 1; 4; 5; 3; 2
E) 5; 2; 3; 4; 1

Brano 1

Leggere il brano e rispondere a ogni quesito solo in base alle informazioni contenute (esplicitamente o implicitamente) nel brano e non in base a quanto il candidato eventualmente conosca sull'argomento.

[1] A pochi giorni dall'inaugurazione delle Olimpiadi di Pechino, mentre il Comitato Olimpico Cinese dichiara aperta la guerra al doping con ogni mezzo, un team di ricercatori americani annuncia alla stampa di aver raggiunto l'ultima frontiera della chimica applicata allo sport: il doping mirato. Ronald Evans, dell'Howard Hughes Medical Institute di La Jolla (California), ha infatti messo a punto un farmaco capace di migliorare le prestazioni degli atleti sulle gare di resistenza, come la maratona. Per i test, Evans ha somministrato ad alcuni topi una sostanza chiamata GW1516 e li ha fatti "allenare" sulla ruota per 5 settimane. I topi dopati hanno corso fino al 70% in più dei loro compagni, ugualmente allenati ma non trattati con il nuovo farmaco. Evans ha poi somministrato una seconda sostanza ad altri topi: l'AICAR, un acceleratore del metabolismo dei grassi e degli zuccheri. Questi, pur senza alcun allenamento, hanno corso fino al 44% più a lungo dei topi "puliti".

[2] Visti i risultati dei test e – per sua stessa ammissione – vista la relativa semplicità del cocktail dopante, Evans ha sì pubblicato la sua ricerca, ma ha anche fornito alla WADA, l'Agenzia Mondiale Antidoping, le informazioni chiave per identificare queste sostanze nel sangue degli atleti che volessero "accorciare" i loro tempi di preparazione. Obiettivo delle ricerche di Evans, che non è nuovo a studi di questo tipo, è quello di migliorare le condizioni di vita di chi è costretto all'immobilità da incidenti o malattie.

(Da: "Doping: un cocktail bomba oscura le Olimpiadi" di Alessandro Bolla).

17. Qual è l'obiettivo delle ricerche di Evans?

A) Migliorare le prestazioni degli atleti sulle gare di resistenza
B) Aiutare chi non si può muovere a seguito di incidenti o malattie
C) Migliorare le prestazioni dei topi sulla ruota
D) Pubblicare le sue ricerche scientifiche su riviste specializzate
E) Fornire alla WADA informazioni sulle nuove sostanze dopanti utilizzate dagli atleti

18. Secondo quanto riportato nel brano, Evans:
- A) custodisce gelosamente il segreto della formula del doping che ha scoperto
- B) lavora per l'Agenzia mondiale antidoping
- C) ha dato all'Agenzia mondiale antidoping le informazioni necessarie per scoprire eventuali usi delle sostanze dopanti da lui scoperte
- D) è un grande atleta e scienziato
- E) ha effettuato i suoi test prima sui topi e poi sugli sportivi

19. Il brano è verosimilmente tratto da:
- A) un libro sulla storia delle Olimpiadi
- B) una rivista divulgativa
- C) un fascicolo informativo di una campagna contro i test sugli animali
- D) un articolo di cronaca sportiva
- E) una telecronaca in diretta da Pechino

20. Secondo quanto riportato nel brano, il Comitato olimpico cinese:
- A) nessuna delle altre alternative è corretta
- B) è favorevole al doping
- C) ha chiesto l'aiuto dell'Howard Hughes Medical Institute di La Jolla per la guerra al doping
- D) considera possibile il pericolo di doping solo per le gare di atletica
- E) ha diffuso i risultati dei test sul "doping mirato"

Test di Cultura generale

21. Il Dolce Stil novo prende le mosse dalla poesia:
- A) neoclassica
- B) religiosa
- C) didattica
- D) burlesca
- E) cortese

22. La prima guerra d'indipendenza italiana fu combattuta contro:
- A) la Francia
- B) la Prussia
- C) lo Stato Pontificio
- D) la Spagna
- E) l'Austria

Test di Biologia

23. La determinazione del sesso nella specie umana, come in molti altri organismi, dipende:
- A) dall'ovulo
- B) dallo zigote
- C) dagli autosomi
- D) dallo spermatozoo
- E) nessuna delle altre alternative è corretta

24. Funzionalmente, l'emoglobina può essere definita una proteina:
- A) di difesa
- B) di riserva
- C) enzimatica
- D) di trasporto
- E) nessuna delle altre alternative è corretta

25. In ciascun gamete di un essere umano si trovano:
- A) 23 cromosomi
- B) 46 cromosomi
- C) 23 coppie di cromosomi
- D) 46 autosomi
- E) 2 cromosomi sessuali

26. La struttura presente sia nella cellula eucariotica sia nella procariotica è:
- A) nessuna delle altre alternative è corretta
- B) il ribosoma
- C) il mitocondrio
- D) un nucleo ben definito
- E) il nucleolo

27. Il daltonismo è un carattere ereditario legato al cromosoma X. Se un uomo daltonico sposa una donna sana, cioè che non presenta il carattere recessivo sul cromosoma X, quale tra le seguenti affermazioni risulta vera?
- A) Le figlie sono portatrici sane del daltonismo
- B) I figli maschi sono portatori sani del daltonismo
- C) Nessuno dei figli e delle figlie porta il gene alterato
- D) I figli maschi sono daltonici
- E) Le figlie sono daltoniche

28. Da una cellula nervosa viene prelevato materiale patogeno infettante. In esso viene isolata una proteina con una sequenza di amminoacidi uguale a quella di una proteina presente nella cellula ospite, ma non viene riscontrata la presenza di acidi nucleici.
Cosa si può dedurre?
- A) Il materiale infettante è costituito da un viroide
- B) Il materiale infettante è costituito da un prione
- C) Si può escludere che il materiale infettante sia un virus, un batterio, un fungo o un prione perchè in ogni caso si sarebbe dovuta riscontrare la presenza di acidi nucleici
- D) Il materiale infettante è costituito dal residuo di un chemioterapico, probabilmente un anticorpo monoclonale
- E) Non si può mai riscontrare una situazione come quella descritta perché non esistono organismi privi di acidi nucleici

29. Il rene svolge tutte le seguenti funzioni TRANNE:
- A) intervenire nel mantenimento dell'equilibrio idrico-salino dell'organismo
- B) intervenire nella regolazione ormonale della pressione sanguigna
- C) eliminare molecole introdotte con la dieta e non digerite
- D) provvedere all'escrezione dei prodotti del catabolismo cellulare
- E) intervenire nella regolazione della quantità di globuli rossi presenti nel sangue attraverso la secrezione di eritropoietina

30. I lisosomi sono:
- A) enzimi secreti dal pancreas
- B) enzimi prodotti dalla tiroide
- C) organuli citoplasmatici contenenti enzimi idrolitici
- D) organuli presenti nel nucleo
- E) cellule prive di nucleo

31. Lo zigote è la cellula diploide prodotta dalla fusione di:
- A) due cellule autosomiche
- B) due cellule eterotrofe
- C) due cellule zigomatiche
- D) quattro gameti
- E) due gameti

32. Le proteine sono polimeri di amminoacidi legati covalentemente mediante legame:
- A) peptidico
- B) fosfodiesterico
- C) idrogeno
- D) tioesterico
- E) ionico

33. Il crossing-over è un processo che:
- A) si realizza nel corso dell'anafase mitotica
- B) porta a un aumento della variabilità genetica
- C) caratterizza tanto la mitosi quanto la meiosi
- D) dà luogo alla formazione di cromosomi identici a quelli presenti prima del crossing-over
- E) porta a un aumento del numero di cromosomi aploidi

34. Relativamente alla cellula, per "trasporto attivo" si intende:
- A) l'eliminazione, attraverso la membrana plasmatica, di sostanze di rifiuto
- B) il libero passaggio di molecole e ioni attraverso la membrana plasmatica
- C) il passaggio di acqua attraverso la membrana plasmatica
- D) il passaggio di sostanze attraverso la membrana plasmatica contro gradiente di concentrazione
- E) il passaggio di sostanze attraverso la membrana plasmatica secondo gradiente di concentrazione e con consumo di ATP

35. La parete cellulare delle cellule vegetali è formata principalmente da:
- A) fosfolipidi
- B) glicogeno
- C) cellulosa
- D) collagene
- E) elastina

36. Quale delle seguenti alternative rappresenta l'equazione globale della fotosintesi?
- A) $6\ CO_2 + 6\ H_2O \rightarrow C_6H_{12}O_6 + 6\ O_2$
- B) $C_6H_{12}O_6 + 6\ O_2 \rightarrow 6\ CO_2 + 6\ H_2O$
- C) $6\ CO_2 + 6\ O_2 \rightarrow C_6H_{12}O_6 + 6\ H_2O$
- D) $6\ CO_2 + 6\ H_2O \rightarrow 6\ C_6H_{12}O_6 + 6\ O_2$
- E) $6\ CO_2 + 6\ H_2O + 6\ O_2 \rightarrow C_6H_{12}O_6$

37. Solo una delle seguenti classi di sequenze nucleotidiche è comune alle librerie genomiche e di cDNA:

A) introni
B) sequenze ripetute in tandem
C) sequenze intergeniche
D) esoni
E) promotori

38. Una sostanza o agente chimico sono teratogeni se:

A) sono in grado di produrre un aumento del numero di tumori e in grado comunque di danneggiare le cellule umane
B) per inalazione, ingestione e penetrazione cutanea possono produrre difetti genetici ereditari o aumentarne la frequenza
C) al contatto diretto, prolungato o ripetuto con la pelle o con le mucose, possono provocare una reazione infiammatoria
D) possono provocare anomalie nello sviluppo di un embrione, di un feto o di un neonato
E) nessuna delle altre alternative è corretta

39. Quale delle seguenti molecole NON contiene azoto?

A) Un peptide
B) Un enzima
C) Una proteina
D) Un trigliceride
E) Un nucleoside

40. Qual è la funzione del tRNA?

A) Trasportare l'informazione genetica dal nucleo (DNA) al ribosoma
B) Fungere da stampo per la sintesi del DNA
C) Trasportare gli aminoacidi al ribosoma
D) Formare i ribosomi complessandosi alle proteine ribosomali
E) Intervenire nella sintesi proteica nel ribosoma con funzioni catalitiche

Test di Chimica

41. Durante le reazioni chimiche, gli atomi:

A) subiscono processi che coinvolgono le particelle del nucleo
B) subiscono variazioni del numero atomico
C) conservano invariata la configurazione elettronica
D) conservano invariata la configurazione elettronica esterna
E) possono subire variazioni del numero di elettroni che circondano il nucleo

42. Il cesio è il metallo alcalino più elettropositivo, il fluoro l'alogeno più elettronegativo. Tra loro quindi:

A) formano legami covalenti
B) non posso formare legami
C) formano legami di coordinazione
D) formano legami ionici
E) formano legami sia ionici sia covalenti

43. Quale tra le seguenti reazioni NON è tipica degli alcani?
A) Sostituzione
B) Addizione elettrofila
C) Deidrogenazione
D) Alogenazione
E) Combustione

44. Il composto HCOOH è:
A) un fenolo
B) un alcool
C) un'ammina
D) l'acido carbonico
E) un acido carbossilico

45. Per ottenere un sale si devono far reagire:
A) nessuna delle altre alternative è corretta
B) un idrossido e l'acqua
C) un acido e l'acqua
D) un acido e un'anidride
E) un acido e un idrossido

46. Come viene chiamato il passaggio dallo stato liquido allo stato aeriforme?
A) Evaporazione
B) Condensazione
C) Fusione
D) Brinamento
E) Sublimazione

47. Quale dei seguenti elementi è un alogeno?
A) Co
B) B
C) At
D) Ti
E) Ag

48. Variando il valore di pH di una soluzione da 4 a 6 la concentrazione degli ioni idrogeno in soluzione:
A) aumenta di 100 volte
B) si dimezza
C) diminuisce di 100 volte
D) diminuisce di 2 volte
E) aumenta di 2 volte

49. Le proprietà chimiche degli elementi sono determinate:
A) dal numero atomico
B) dalla tavola periodica degli elementi
C) dal numero di massa
D) dal loro pH
E) dal numero di neutroni presenti nel nucleo

50. Il sale da cucina è il cloruro di sodio, NaCl, solubile in acqua liquida fino a una concentrazione massima di 348 g per kg di acqua a temperatura ambiente.
Se si prova a scioglierne in quantità maggiore senza variare la temperatura:

A) il sale in eccesso galleggia sulla superficie della soluzione
B) l'acqua comincia a bollire
C) l'acqua solidifica
D) il sale in eccesso si raccoglie sul fondo del recipiente
E) il sale evapora

51. Indicare l'idrocarburo.

A) CH_3CH_2OH
B) $HCOOCH_2$
C) CH_3OH
D) C_6H_{14}
E) CH_3COOH

52. Cosa si ottiene per combustione del metano?

A) CO_2 e O_2
B) H_2O e O_2
C) CO_2 e H_2
D) CH_4 e O_2
E) CO_2 e H_2O

<u>Test di Fisica e Matematica</u>

53. Individuare fra le seguenti l'espressione NON equivalente a 0,006:

A) 60/10.000
B) 6/10.000
C) 6/1.000
D) 600/100.000
E) 0,600/100

54. La soluzione dell'equazione 6x + 11 = 2x – 1 è:

A) 2
B) –1
C) 3
D) 1
E) –3

55. L'equazione $3 - e^{(x-1)} = 1$:

A) ha un'unica soluzione che è diversa da $x = \log_e 3$
B) ha l'unica soluzione $x = \log_e 3$
C) ha infinite soluzioni
D) non ha soluzioni
E) ha due soluzioni distinte

56. Stabilire quale, tra le seguenti curve, NON è un'iperbole.

A) $x^2/2 + y^2/3 = 1$
B) $x^2 – y^2 = 15$
C) $x^2/2 – y^2/3 = 1$
D) $xy = 2$
E) $x^2 – y^2 = 1$

57. Ponendo in parallelo tre condensatori di capacità uguale a 60 microfarad ciascuno, la capacità totale vale:

A) 20 microfarad
B) 60 microfarad
C) 180 microfarad
D) 400 microfarad
E) 120 microfarad

58. Sulle altalene una mamma applica la stessa forza di spinta a una bambina che pesa 13 kg e a un bambino che pesa 26 kg. L'accelerazione impressa a ognuno di essi è:

A) inversamente proporzionale alla massa
B) proporzionale al quadrato della massa
C) indipendente dalla massa
D) la medesima
E) proporzionale alla massa

59. Ciascuna delle tre resistenze del circuito rappresentato in figura può dissipare una potenza massima W. Qual è la potenza massima che il circuito risultante può dissipare?

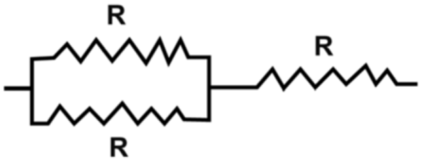

A) (2/3)W
B) 3W
C) (3/2)W
D) W
E) 2W

60. Un corpo di volume V viene immerso in un liquido. Se il corpo affonda, quale delle seguenti relazioni che legano il peso specifico del corpo P_C al peso specifico del liquido P_L del liquido è corretta?

A) $P_C < P_L$
B) $P_C = P_L$
C) $9{,}81 \cdot P_C = P_L$
D) $P_C > P_L$
E) $V \cdot P_C = P_L$

********** FINE DELLE DOMANDE **********

PROVA DI AMMISSIONE AI CORSI DI LAUREA DELLE PROFESSIONI SANITARIE

Anno Accademico 2016/2017

Test di Ragionamento logico

1. **“Se piove non usciamo di casa”. Se la precedente affermazione è vera, quale delle seguenti è necessariamente vera?**
   A) Se non usciamo di casa allora piove
   B) Se non piove usciamo di casa
   C) Se usciamo di casa allora non piove
   D) Non usciamo di casa solo quando piove
   E) Se e solo se piove usciamo di casa

2. **“Non si può non dimostrare la non estraneità dell'imputato al delitto”. La precedente affermazione è equivalente a:**
   A) il delitto è stato compiuto con l'ausilio determinante dell'imputato
   B) l'imputato è certamente da assolvere
   C) l'imputato potrebbe non avere a che fare alcunché con il delitto
   D) l'imputato non è estraneo al delitto
   E) l'imputato è estraneo al delitto

3. **“Veronica è una brava studentessa. Tutti gli studenti di Farmacia sono bravi studenti. Tutti i bravi studenti sono preparati”. Date tali premesse, si può dedurre che:**
   A) solo alcuni studenti di Farmacia sono preparati
   B) tutti gli studenti preparati sono bravi studenti
   C) Veronica è una studentessa di Farmacia
   D) Veronica è preparata
   E) solo alcuni bravi studenti sono preparati

4. **“È sbagliato non ammettere che la scarsità di neve non è bastata per impedire lo svolgimento regolare della gara sciistica”. Basandosi sulla precedente affermazione, individuare quale delle seguenti alternative è esatta.**
   A) La gara sciistica si è svolta regolarmente nonostante la scarsità di neve
   B) Bisogna ammettere che la gara sciistica non si è svolta regolarmente
   C) Si può affermare che la scarsità di neve è stata sufficiente a impedire lo svolgimento regolare della gara sciistica
   D) La scarsità di neve ha di fatto impedito lo svolgimento regolare della gara sciistica
   E) Grazie all'abbondanza di neve la gara sciistica si è svolta regolarmente

5. **Un autobus ha percorso i 5/9 del suo tragitto. Se ha percorso 1.500 metri, quanti metri deve ancora percorrere l'autobus per completare il tragitto?**
   A) 2.400
   B) 1.200
   C) 1.750
   D) 2.700
   E) 3.500

6. Cinque amici, Antonio, Vincenzo, Luigi, Diego e Sandro, sono nati in cinque città diverse: Perugia, Bari, Catania, Genova e Venezia, non necessariamente in questo ordine. Tutti hanno un'età diversa: 28, 26, 25, 24 e 22 anni, non necessariamente in questo ordine. Si sa che: Diego è il più giovane ed è nato in una città meridionale; Luigi è il più anziano ed è nato a Venezia; chi è nato a Catania ha 2 anni meno di Luigi e non si chiama Antonio; Vincenzo proviene da Perugia e ha un'età divisibile per 5.
In base alle precedenti informazioni, quanti anni ha Antonio e in quale città è nato?

A) 26 anni ed è nato a Genova
B) 24 anni ed è nato a Genova
C) 24 anni ed è nato a Bari
D) 26 anni ma non si può stabilire dove è nato
E) 26 anni ed è nato a Bari

7. Un bicchiere d'acqua pesa 150 grammi. Sapendo che il peso del bicchiere vuoto rappresenta il 30% del peso totale, qual è il peso dell'acqua?

A) 105 grammi
B) 130 grammi
C) 100 grammi
D) 115 grammi
E) 120 grammi

8. Quali, tra i termini proposti, completano correttamente la seguente proporzione verbale?
sasso : ghiaione = X : Y

A) X = frana; Y = slavina
B) X = calciatore; Y = allenamento
C) X = parola; Y = sillaba
D) X = mucca; Y = mandria
E) X = marmo; Y = sabbia

9. Liam ha 6 accendini apparentemente identici, uno dei quali è però più pesante degli altri.
Avendo a disposizione una bilancia a due piatti, quante pesate saranno sufficienti per essere certi di individuarlo?

A) 2
B) 3
C) 4
D) 5
E) 6

10. Quali tra i termini proposti completano correttamente la seguente proporzione verbale?
inetto : X = Y : esiziale

A) X = incapace; Y = meritato
B) X = assente; Y = presente
C) X = inane; Y = esistenziale
D) X = investito; Y = essenziale
E) X = adatto; Y = salutare

11. Ascanio deve raggiungere i colleghi che si trovano a un meeting in una località inglese. Giunto all'aeroporto di Londra decide di prendere una macchina a noleggio. La benzina in Inghilterra costa 3 sterline al gallone. Si sa che Londra e la località da raggiungere distano 240 miglia, che ogni gallone equivale a circa 3,75 litri di benzina, che una sterlina vale 1,25 euro e che la macchina consuma 1 litro di benzina ogni 20 km. Sapendo che un chilometro equivale a 0,60 miglia, quale sarà la spesa (in euro) per la benzina sostenuta da Ascanio per raggiungere i colleghi da Londra e per tornare all'aeroporto?

A) 36 euro
B) 40 euro
C) 32 euro
D) 44 euro
E) 20 euro

12. Qual è stata la percentuale di sconto se un oggetto che costava 50 euro viene venduto a 32 euro?

A) 72%
B) 18%
C) 36%
D) 9%
E) 54%

13. Un'enciclopedia è composta di 8 volumi, ognuno di 600 pagine. Se i volumi sono disposti normalmente affiancati in una libreria, quante pagine ci sono contando tutte le pagine nell'intervallo fra la prima pagina del secondo volume e l'ultima pagina del quarto volume?

A) 1.200
B) 0
C) 600
D) 1.800
E) 2.400

14. "Non esiste favola senza lieto fine". Se la precedente affermazione è FALSA, quale delle seguenti è necessariamente vera?

A) Tutte le favole hanno un lieto fine
B) Nessuna favola ha un lieto fine
C) Esiste almeno una favola senza lieto fine
D) Tutte le favole sono senza lieto fine
E) Tutte le storie con un lieto fine sono favole

15. Nel luglio del 1984 alcuni scienziati trovarono a Nairobi uno scheletro di "Homo erectus" ben conservato risalente a 1.600.000 anni fa. Lo scheletro apparteneva a un individuo dell'altezza di 1,82 metri. Le sue ossa furono ritrovate in una palude. Esse erano, per dimensione e per forma, come quelle di un uomo moderno. Sotto le ossa fu trovato materiale vulcanico derivante da un'eruzione avvenuta 1.650.000 anni fa.
Quale delle seguenti affermazioni può essere ricavata dal testo su riportato?

A) Gli individui appartenenti alla specie umana non assomigliavano all'uomo attuale
B) La specie umana non esisteva 1,6 milioni di anni fa
C) La specie umana è aumentata di statura spontaneamente nel corso dei millenni
D) L'uomo ha raggiunto le sue dimensioni attuali più di 1.500.000 anni fa
E) La specie umana ha avuto origine in Europa

16. Se la lettera N identifica una qualunque cifra numerica (singola), la lettera P identifica una qualunque cifra (singola) pari e la lettera D identifica una qualunque cifra (singola) dispari, allora DPDD è un numero:

A) divisibile per 2
B) pari di 4 cifre
C) dispari di 3 cifre
D) dispari di 2 cifre
E) dispari di 4 cifre

Brano I

Leggere il brano e rispondere a ogni quesito solo in base alle informazioni contenute (esplicitamente o implicitamente) nel brano e non in base a quanto il candidato eventualmente conosca sull'argomento.
La torbiera? Un ambiente pericoloso, minaccioso, oscuro. Persino Frodo, il protagonista del Signore degli anelli, ha corso il rischio di morire in una torbiera (le "Paludi morte"). Eppure, visti con gli occhi di un ricercatore, pochi ambienti sono più affascinanti di queste distese di muschi in decomposizione, di piccoli cespugli e di specie vegetali e animali molto adattate. E le torbiere non sono neppure ambienti marginali: coprono infatti 400 milioni di ettari delle terre emerse, in gran parte nelle nazioni più vicine al Polo Nord, come Russia e Finlandia, e rappresentano circa il 50% delle zone umide del mondo.
Le torbiere si creano quando il territorio umido, come un lago non molto profondo o una palude, è lentamente ricoperto da uno strato di muschi che, nel giro di migliaia di anni, vanno a riempire l'intero bacino. L'ambiente che si forma è una strana combinazione di morte e vita, perché il muschio, di solito del genere Sphagnum, è sepolto nelle profondità a mano a mano che i suoi piccoli steli crescono verso la luce. Si forma così un tappeto di piante vive che ricopre metri e metri di materiale spugnoso che si decompone a fatica. Lontani dall'ossigeno dell'aria, infatti, i muschi e i resti organici sepolti rimangono intatti per secoli. È un ambiente a volte estremamente acido, che quindi ospita specie adatte a un ecosistema particolare. Le piante, per esempio, devono riuscire a sopravvivere con poco azoto, che si trova solo nei terreni ricchi di materiale organico decomposto. Per questo nelle torbiere vivono spesso piante carnivore come la Drosera, che estraggono l'azoto dalle loro prede: piccoli animali come insetti o ragni.
L'estrema immobilità delle torbiere le ha trasformate in un vero e proprio archivio vivente. Scavare nello strato, profondo metri, di stagni morti o in via di decomposizione, permette di risalire, quasi anno per anno, ai cambiamenti di vegetazione avvenuti nel corso dei secoli (spesso la torba ha cominciato a depositarsi oltre 10 mila anni fa). Il polline, le foglie o i fiori delle piante testimoniano le modifiche nel clima della regione.
E a volte è possibile trovare corpi umani, le "mummie delle torbiere", che secondo alcuni studi sono abitanti locali dell'età del ferro, forse sacrificati durante misteriose cerimonie.

(M. Ferrari, «Focus»)

17. La torbiera viene definita come una combinazione di morte e di vita perché: (vedi Brano I)

A) insetti e ragni si nutrono di piante morte
B) le specie animali che vi si trovano si nutrono di materiale in decomposizione
C) in superficie vi è materiale in lenta decomposizione e in profondità piante vive
D) le specie vegetali che vi si trovano prosperano grazie al materiale in decomposizione
E) in superficie vi sono piante vive e in profondità materiale in lentissima decomposizione

18. Quale delle seguenti affermazioni sulle torbiere è FALSA? (vedi Brano I)
- A) Sono tipiche di zone particolarmente ricche di luce
- B) Costituiscono circa le metà delle zone umide del mondo
- C) Coprono più di 300 milioni di ettari delle terre emerse
- D) Spesso costituiscono un ambiente acido
- E) Non vi si trovano piante d'alto fusto

19. Stando al contenuto del brano I, come si origina una torbiera?
- A) Attraverso la colonizzazione di un terreno umido da parte di un muschio chiamato Sphagnum. In seguito, il tappeto di muschio viene colonizzato anche da ragni, insetti e da una pianta carnivora, la Drosera
- B) Mediante la proliferazione di piante che necessitano di poco azoto in una zona umida e depositano, strato dopo strato, resti di polline, foglie e fiori, che andranno a formare un tappeto ideale per la proliferazione del muschio
- C) Attraverso la lenta proliferazione dei muschi che, nel corso di migliaia di anni, arrivano a ricoprire per intero la superficie di uno specchio d'acqua poco profondo
- D) Mediante la rapida colonizzazione di una palude o di un lago poco profondo da parte di muschi, piante che cercano terreni acidi e piante carnivore
- E) Nessuna delle altre alternative è corretta

20. La torbiera può essere definita un archivio vivente perché: (vedi Brano I)
- A) nell'età del ferro veniva usata come cimitero
- B) vi si trova una grandissima varietà di specie animali e vegetali
- C) vi sono stati rinvenuti resti di corpi umani risalenti all'età preistorica
- D) lontani dall'ossigeno e dall'acqua, i muschi e i resti organici si conservano per secoli
- E) lontani dall'ossigeno dell'aria, i muschi e i resti organici sepolti si conservano per secoli

Test di Cultura generale

21. Quale delle seguenti opere NON è stata scritta da Leonardo Sciascia?
- A) La scomparsa di Majorana
- B) Il giorno della civetta
- C) A ciascuno il suo
- D) La cognizione del dolore
- E) Todo modo

22. Le leggi razziali approvate dal regime fascista in Italia costituirono:
- A) delle misure di discriminazione e persecuzione ai danni della popolazione di religione ebraica
- B) delle misure di chiusura dell'immigrazione alle persone di colore
- C) delle misure di selezione genetica per contrastare i matrimoni misti tra italiani e stranieri
- D) un provvedimento imposto da Hitler dopo la Conferenza di Monaco
- E) il preludio alla rottura delle relazioni tra Mussolini e Hitler

Test di Biologia

23. Quale delle seguenti strutture è una ghiandola?
- A) Colon
- B) Epiglottide
- C) Cistifellea
- D) Pancreas
- E) Pleura

24. Gli anticorpi sono:
A) nucleotidi
B) polisaccaridi
C) fosfolipidi
D) proteine
E) amminoacidi

25. Quale delle seguenti affermazioni relative alle malattie autoimmuni è corretta?
A) Sono caratterizzate dalla produzione di autoanticorpi
B) Consistono in una risposta immunitaria verso antigeni esogeni
C) Insorgono nel periodo perinatale di immaturità immunologica
D) Si verificano quando si instaura una tolleranza indotta da bassa dose di antigene
E) Derivano da eccessiva selezione clonale

26. L'unità strutturale e funzionale dei viventi è:
A) il genoma
B) la cellula
C) il DNA
D) l'RNA
E) la molecola

27. Il flusso dell'informazione genetica si verifica da:
A) RNA a proteine a DNA
B) proteine a RNA a DNA
C) DNA a proteine a RNA
D) RNA a DNA a proteine
E) DNA a RNA a proteine

28. I gameti sono:
A) le cellule sessuali sia femminili sia maschili
B) l'insieme degli organi dell'apparato genitale maschile
C) solo le cellule sessuali maschili
D) le cellule riproduttive nelle specie che hanno riproduzione asessuata
E) solo le cellule sessuali femminili

29. Gli individui di gruppo sanguigno 0:
A) hanno sicuramente qualche parente di gruppo sanguigno 0
B) possono avere figli esclusivamente di gruppo sanguigno 0
C) hanno sempre entrambi i genitori di gruppo sanguigno 0
D) hanno ereditato un allele di tipo 0 da ciascun genitore
E) hanno necessariamente almeno un genitore di gruppo sanguigno 0

30. La validità della legge di Mendel, detta dell'assortimento indipendente, può essere verificata sperimentalmente solo nel caso di caratteri codificati da geni localizzati:
A) sugli stessi cromatidi
B) su cromosomi omologhi
C) sugli stessi cromosomi
D) su cromosomi diversi
E) su mitocondri

31. Il trasporto attivo delle sostanze nutritive all'interno della cellula batterica:
- A) avviene con esclusivo utilizzo di meccanismo di tipo “simporto”
- B) avviene sempre con modificazione del substrato
- C) avviene con esclusivo utilizzo di meccanismo di tipo “uniporto”
- D) avviene secondo gradiente di concentrazione
- E) necessita di un dispendio energetico per il trasporto

32. Come si dividono le cellule procariote?
- A) Per scissione semplice
- B) Per mitosi
- C) Per meiosi
- D) Per fissione binaria
- E) Nessuna delle altre alternative è corretta

33. Le elicasi nella duplicazione del DNA:
- A) rompono i legami fosfodiesterici tra i nucleotidi dello stesso filamento
- B) rompono i legami covalenti che mantengono unite le due emieliche
- C) rompono i legami a idrogeno che mantengono unite le due emieliche
- D) mantengono distese le due emieliche del DNA
- E) rompono i legami a idrogeno tra i nucleotidi dello stesso filamento

34. Quale delle seguenti affermazioni sulla RNA polimerasi è corretta?
- A) Trascrive sia gli esoni sia gli introni
- B) Può produrre molte catene polipeptidiche contemporaneamente tramite la formazione di poliribosomi
- C) Produce solo mRNA
- D) Ha una funzione nella traduzione
- E) Crea legami covalenti tra i nucleotidi del DNA e i nucleotidi complementari dell'RNA

35. Che cos'è il peptidoglicano?
- A) Una glicoproteina che viene sintetizzata dai batteri
- B) Il componente fondamentale della parete cellulare delle cellule vegetali
- C) Il componente fondamentale della parete cellulare batterica
- D) Il componente fondamentale della parete cellulare dei soli batteri gram-negativi
- E) Il componente fondamentale della parete cellulare delle cellule animali

36. Gli amminoacidi “non essenziali” sono quegli amminoacidi:
- A) di cui è meglio evitare la somministrazione con la dieta
- B) che danno origine ai corpi chetonici
- C) di cui l'organismo può fare a meno
- D) che fanno parte di proteine non essenziali
- E) che l'organismo è capace di sintetizzare

37. La mitosi è:
- A) la fase del ciclo dove avviene la sintesi del DNA
- B) sinonimo di ciclo cellulare
- C) la divisione delle sole cellule epidermiche
- D) nessuna delle altre alternative è corretta
- E) un processo di divisione cellulare

38. L'ATP è:

A) un monomero di acido nucleico (DNA e RNA)
B) un nucleotide trifosfato
C) nessuna delle altre alternative è corretta
D) un nucleoside
E) un acido trifosforico

39. I linfociti sono cellule:

A) epiteliali
B) della glia
C) epatiche
D) muscolari
E) del sistema immunitario

40. Cosa sono i vertebrati terrestri, e quindi l'uomo?

A) Ammoniotelici
B) Uricotelici
C) Ureotelici
D) Ureotelici, ammoniotelici e uricotelici
E) Nessuna delle altre alternative è corretta

Test di Chimica

41. Nella seguente reazione: 2 NaCl + $Br_2 \rightarrow Cl_2$ + 2 NaBr:

A) il cloro si riduce e il sodio si ossida
B) il cloro si ossida e il bromo si riduce
C) il bromo e il cloro si ossidano e il sodio si riduce
D) non si hanno ossidazioni né riduzioni
E) il cloro si riduce e il bromo si ossida

42. Due soluzioni, non elettrolitiche, separate da una membrana semipermeabile, si dicono isotoniche:

A) se contengono sostanze differenti ma in concentrazioni uguali
B) se le concentrazioni delle soluzioni rimangono invariate
C) se contengono la stessa sostanza in concentrazioni differenti
D) se il soluto passa dalla soluzione più concentrata a quella più diluita
E) in nessuno dei casi indicati nelle altre alternative

43. Quale tra le seguenti è la formula di un composto avente formula minima CH_2O e peso molecolare 180?

A) $C_2H_4O_2$
B) CH_2O
C) $C_3H_6O_3$
D) $C_6H_{12}O_6$
E) $C_8H_4O_8$

44. Se la soluzione acquosa 0,1 M di una sostanza ha un pH uguale 4,5, la sostanza in soluzione è:

A) un acido debole
B) un acido forte
C) una sostanza neutra
D) una base di Lewis
E) acido cloridrico

45. La molecola biatomica del cloro (Cl_2) contiene un legame:
A) covalente eteropolare
B) covalente omopolare
C) covalente doppio
D) dativo
E) ionico

46. L'elemento più elettronegativo è:
A) il carbonio
B) l'ossigeno
C) l'idrogeno
D) l'elio
E) il fluoro

47. Gli isotopi 12 e 14 del carbonio differiscono tra loro per:
A) un protone
B) due protoni
C) due neutroni
D) un protone e un neutrone
E) il numero di elettroni

48. Quale dei seguenti composti organici presenta nella sua molecola un triplo legame C-C?
A) CH_4
B) $C_2H_5NH_2$
C) C_2H_2
D) HCOOH
E) CH_3NH_2

49. Gli amminoacidi si legano tra loro per formare:
A) i polisaccaridi
B) i polimeri acidi
C) le proteine
D) le ammine
E) le ammidi

50. Una soluzione acquosa di KBr rispetto all'acqua pura ha:
A) lo stesso punto di ebollizione
B) lo stesso punto di congelamento
C) punto di ebollizione più alto
D) volume maggiore
E) punto di ebollizione più basso

51. La temperatura di fusione dell'acqua è la temperatura alla quale:
A) l'acqua liquida evapora
B) il ghiaccio si trasforma in gas
C) il vapor acqueo liquefà
D) l'acqua liquida congela
E) coesistono i tre stati liquido, solido e aeriforme

52. La somma algebrica dei numeri di ossidazione degli atomi di NH_4^+ è:

A) 0
B) +1
C) –1
D) –4
E) +4

Test di Fisica e Matematica

53. Sapendo che l'accelerazione di gravità vale 9,8 m/s^2, qual è il peso di una valigia con massa uguale a 10 kg?

A) Nessuna delle altre risposte è corretta
B) 980 N
C) 98 N
D) 9,8 N
E) 0,98 N

54. Un corpo ha una massa di 30 g e un volume di 50 cm^3. Ponendolo in acqua, che cosa succede?

A) Resta sospeso in prossimità della superficie
B) Nessuna delle altre alternative è corretta
C) Viene sommerso in profondità
D) Affonda
E) Galleggia

55. La somma di due vettori aventi lo stesso punto di applicazione è il vettore:

A) dato dalla diagonale del parallelogramma costruito su di essi
B) avente per modulo la somma dei moduli e direzione parallela a entrambi i vettori
C) avente per modulo la somma dei moduli e per direzione quella del vettore di modulo maggiore
D) avente per modulo la somma dei moduli e direzione perpendicolare a entrambi i vettori
E) avente per modulo la differenza dei moduli e per direzione quella del vettore di modulo maggiore

56. La resistenza di un filo lungo 6 metri è pari a 10 ohm. Se si applica ai suoi estremi una differenza di potenziale pari a 20 V, quanto vale la corrente che attraversa il filo?

A) 20 A
B) 20 W
C) 0,5 A
D) 2 A
E) 10 W

57. La disequazione $(x + 2)(x + 3) < 0$ è verificata per:

A) $x < -3$
B) $x < -3$; $x > -2$
C) $-3 < x < -2$
D) $x < 2$
E) $x > 3$

58. L'equazione di secondo grado $x^2 + 1 = 0$, nell'insieme dei numeri reali:

A) è verificata soltanto per $x = -1$
B) è sempre verificata
C) è verificata per $x = 1$ e $x = -1$
D) è verificata soltanto per $x = 1$
E) non è mai verificata

59. Ridurre ai minimi termini (semplificare) la frazione algebrica $(9x^2 - 1) / (12x^2 - 4x)$.

A) $(3x + 1) / 4x$
B) $(3x - 1) / 4x$
C) $(3x - 1) / (4x - 4)$
D) $(9x - 1) / (12x - 4)$
E) $(9x - 1) / (x - 3)$

60. Sia $f(x) = (x^2 - 1)^{1/3}$, allora $f(3)$ vale:

A) 1
B) 4
C) 2
D) ± 2
E) $2\sqrt{2}$

********** FINE DELLE DOMANDE **********

PROVA DI AMMISSIONE AI CORSI DI LAUREA DELLE PROFESSIONI SANITARIE

Anno Accademico 2015/2016

Test di Ragionamento logico

1. **Una squadra di 15 operai edifica un palazzo in 30 giorni. Quanti giorni avrebbe impiegato una squadra di 9 operai?**
   A) 60
   B) 40
   C) 50
   D) 45
   E) 18

2. **Indicare quale, fra i termini proposti, completa logicamente la seguente proposizione verbale: trama : vello = brama : X**
   A) X = merlo
   B) X = bello
   C) X = spello
   D) X = agnello
   E) X = pelo

3. **Quale, tra le coppie di termini proposti, completa logicamente la seguente proporzione verbale? X : intonso = territorio: Y**
   A) X = libro; Y = inesplorato
   B) X = capitolo; Y = regione
   C) X = intatto; Y = selvaggio
   D) X = libraio; Y = mappa
   E) X = cultura; Y = geografia

4. **Carlo, Dario, Paolo e Ugo sono quattro atleti che partecipano a una gara di velocità su pista. Sono date le seguenti premesse:**
   – Paolo arriva prima di Carlo;
   – Carlo arriva prima di Dario;
   – Ugo arriva prima di Carlo;
   – La corsa non è stata vinta da Ugo.
   Quale, tra le seguenti affermazioni, NON è corretta?
   A) Ugo è arrivato dopo Dario
   B) L'ordine di arrivo non è alfabetico
   C) Paolo è il vincitore della corsa
   D) Ugo non è arrivato per ultimo
   E) Ugo è arrivato prima di Dario

5. **In una scuola elementare, frequentata da 245 alunni, sono stati attivati due corsi pomeridiani. Si sa che 196 alunni frequentano il corso di spagnolo, 176 il corso di nuoto, 34 nessuno dei due corsi.**
Quanti alunni frequentano entrambi i corsi?
   A) 211
   B) dati sono insufficienti per rispondere al quesito
   C) 35
   D) 161
   E) 15

6. **Un gioco ha le seguenti regole: se un numero è divisibile per 2 vale 6 punti; se è divisibile per 6 vale 4 punti; se è divisibile per 4 vale 2 punti. In base a tali regole, quanto vale il numero 44?**
   A) 0 punti
   B) 6 punti
   C) 4 punti
   D) 10 punti
   E) 8 punti

7. **"Se la giornalista non avesse trovato il messaggio, non avrebbe potuto evitare che la notizia si diffondesse". In base alla precedente affermazione, quale delle seguenti è certamente vera?**
   A) Essendosi diffusa la notizia, allora la giornalista non ha trovato il messaggio
   B) È possibile che la notizia non si sarebbe diffusa se la giornalista non avesse trovato il messaggio
   C) Avendo trovato il messaggio, la giornalista ha potuto evitare che la notizia si diffondesse
   D) Anche se non avesse trovato il messaggio è possibile che la giornalista avrebbe comunque evitato che la notizia si diffondesse
   E) Il messaggio obbligava la giornalista a non diffondere la notizia

8. **Se le lancette di un orologio segnano le 2.45 di mercoledì, tra 50 ore e 30 minuti saranno:**
   A) le 5.15 di venerdì
   B) le 4.15 di venerdì
   C) le 1.15 di martedì
   D) le 17.15 di sabato
   E) le 5.15 di lunedì

9. **"Se i candidati si applicano, conseguiranno l'iscrizione". Sulla base dell'affermazione precedente, quale delle seguenti affermazioni è vera?**
   A) Se un candidato si applica, conseguirà il massimo risultato
   B) Se un candidato è stato iscritto è possibile che si sia applicato
   C) Tutte le altre affermazioni sono vere
   D) Verranno iscritti solo candidati che si sono applicati
   E) Se un candidato non si applica, verrà escluso

10. **In base alle informazioni in suo possesso, il professore ordinario non può non negare che è falso quanto affermato dal suo ricercatore, il quale dichiarò di non conoscere l'autore della scoperta del secolo. Basandosi sulla precedente affermazione, individuare quale delle seguenti alternative è esatta.**
   A) Il ricercatore conosce l'autore della scoperta del secolo
   B) Non è possibile sapere se il ricercatore conosce l'autore della scoperta del secolo
   C) Il ricercatore non conosce l'autore della scoperta del secolo
   D) Il ricercatore è l'autore della scoperta del secolo
   E) Nessuna delle altre alternative è corretta

11. Una società che inizialmente fatturava 200 milioni di euro ha visto calare del 20% il fatturato nel primo anno, del 10% nel secondo e del 50% nel terzo. Qual è il suo fatturato alla fine del terzo anno?

A) 128 milioni di euro
B) 54 milioni di euro
C) 72 milioni di euro
D) 64 milioni di euro
E) 40 milioni di euro

12. "Francesca è alta. Le bionde sono tutte carine. Le persone alte sono agili". Se le precedenti informazioni sono corrette, quale delle seguenti informazioni addizionali NON consentirebbe di concludere che Francesca è carina?

A) Francesca è bionda
B) Le persone alte sono bionde
C) Le persone alte sono carine
D) Le persone agili sono bionde
E) Francesca è agile

13. Un'asta di metallo lunga 1 metro è sospesa per il suo centro. A 35 cm dall'estremità destra è agganciato un peso di 5 kg, mentre all'estremità opposta è agganciato un peso di 48 kg. Cosa è necessario fare per equilibrare l'asta e mantenerla in posizione orizzontale?

A) Aggiungere, al peso agganciato a sinistra, un ulteriore peso di 155 kg
B) Aggiungere, al peso agganciato a destra, un ulteriore peso di 155 kg
C) Aggiungere, al peso agganciato a destra, un ulteriore peso di 160 kg
D) Aggiungere, al peso agganciato a sinistra, un ulteriore peso di 160 kg
E) Nulla, l'asta è già in equilibrio

14. La mamma di Giulia ha 30 anni, ossia la metà degli anni di suo padre Paolo (nonno di Giulia), che ha il triplo degli anni di sua nipote Sandra, la quale ha 7 anni in più del fratello Giacomo, il quale ha 9 anni in più di Giulia. Quanti anni ha quest'ultima?

A) 7
B) 4
C) 6
D) 5
E) 3

15. Si consideri la seguente affermazione: "Nessun gatto ha 3 zampe". Dire che tale affermazione è FALSA equivale ad affermare che:

A) tutti i gatti hanno 4 zampe
B) tutti i gatti hanno un numero di zampe diverso da 3
C) tutti i gatti hanno 3 zampe
D) almeno un gatto ha 3 zampe
E) almeno un gatto ha un numero di zampe diverso da 3

16. L'affermazione "quando dormo molto mi riposo" è equivalente all'affermazione:

A) per me è necessario ma non sufficiente dormire molto per riposarmi
B) se non sono riposato allora non ho dormito molto
C) a volte capita che non mi riposi pur avendo dormito molto
D) dormo molto o mi riposo
E) se sono riposato vuol dire che ho dormito molto

Brano I

Leggere il brano e rispondere ad ogni quesito collegato solo in base alle informazioni contenute.

Sfruttando l'elevata capacità di modificarsi del virus, un laboratorio di Strasburgo, di cui fanno parte due ricercatori italiani, ha individuato un "mutante" in grado di portare alla morte delle cellule tumorali con bassi dosaggi di medicinali.

Usare una malattia per sconfiggerne un'altra può sembrare paradossale, ma presto potrebbe rivelarsi una nuova frontiera delle cure anti-tumore. È la pista seguita dal laboratorio Retrovirus ed evoluzione molecolare di Strasburgo, diretto da Matteo Negroni dell'Istituto di biologia molecolare e cellulare del CNRS (il Centro nazionale francese di ricerca scientifica), che ha modificato il genoma del virus ottenendo ottimi risultati nella morte delle cellule tumorali, con un dosaggio di medicinali inferiore di 300 volte rispetto a quello utilizzato nelle terapie moderne.

L'HIV presenta una caratteristica fondamentale: l'elevata capacità di mutazione. Sfruttando questa peculiarità del virus, i ricercatori ne hanno combinato il genoma, privato della sua forza infettiva, con un gene umano presente in tutte le cellule, la desossicitidina chinasi (o dCK), che funziona da "attivatore" dei medicinali antitumorali. "In questo modo – spiega Matteo Negroni – si è ottenuta una banca dati di ottanta proteine mutanti. Tra loro i ricercatori hanno poi individuato la variante con la capacità maggiore di agire sulle cellule cancerose, uccidendole anche con un basso dosaggio di medicinali".

La proteina modificata, poi, non ha bisogno di essere iniettata in tutte le cellule malate. "Basta infatti che agisca su poche di loro perché, grazie all'effetto conosciuto come bystander, si assista alla morte delle altre cellule", continua lo studioso.

La ricerca, pubblicata su PLoS Genetics, apre prospettive verso un trattamento terapeutico alternativo, in grado di limitare i problemi legati alla tossicità di alcuni medicinali. Gli esperimenti, durati tre anni e guidati da un'altra ricercatrice italiana, Paola Rossolillo, sono stati effettuati solo in provetta.

"Bisognerà aspettare un anno e mezzo, forse due, per iniziare la sperimentazione preclinica sugli animali. Finanziamenti permettendo", conclude Negroni. Test sulla modificazione del virus dell'Aids potrebbero rivelarsi utili anche per la cura di altre malattie.

17. Quale delle seguenti affermazioni è vera? (*vedi Brano I*)

A) Tutte le altre affermazioni sono vere
B) La scoperta di cui si parla nel testo è stata fatta da un'équipe di ricercatori francesi
C) A breve inizieranno i test per verificare la validità dell'esperimento del laboratorio di Strasburgo
D) Grazie alla scoperta fatta, in futuro le cure antitumorali potrebbero essere al tempo stesso più leggere e più efficaci
E) L'effetto bystander impedisce che un medicinale si propaghi e raggiunga le cellule malate

18. Quale delle seguenti alternative sintetizza meglio il modo in cui hanno proceduto i ricercatori? (*vedi Brano I*)

A) Hanno associato il gene della dCK al genoma dell'HIV per ottenere una serie di proteine da cui estrarre quella più tossica per le cellule tumorali
B) Hanno iniziato l'analisi in laboratorio e hanno poi continuato la sperimentazione preclinica sugli animali
C) Hanno analizzato il comportamento del gene della desossicitidina al momento della somministrazione di medicinali antitumorali
D) Hanno studiato l'effetto bystander per capire quante cellule potessero essere eliminate grazie alla proteina dell'HIV
E) Hanno analizzato la banca dati delle proteine mutanti dell'HIV per capire quale potesse essere più efficace nel distruggere le cellule cancerose

19. Secondo quanto riportato nel testo: (*vedi Brano I*)
- A) i medicinali anti-tumore hanno problemi di tossicità
- B) vi sono ben ottanta proteine mutanti efficaci nell'eliminazione delle cellule cancerose
- C) il virus dell'Aids difficilmente potrà essere impiegato per curare altre patologie
- D) la desossicitidina chinasi si trova solo in alcune cellule
- E) Matteo Negroni dirige uno dei laboratori di ricerca dell'ospedale di Strasburgo

20. La proteina modificata non deve essere necessariamente inoculata in tutte le cellule cancerose perché: (*vedi Brano I*)
- A) in parecchi casi è più che sufficiente che agisca su poche cellule per ottenere un buon risultato
- B) l'effetto che ha sulle poche cellule in cui viene inoculata si trasmette anche alle altre
- C) in alta concentrazione potrebbe riacquistare la sua forza infettiva
- D) il livello di tossicità non sarebbe più tollerabile dal corpo del malato
- E) perde di efficacia se viene inoculata numerose volte

Test di Cultura Generale

21. L'espressione "guerra fredda" indica:
- A) la campagna di guerra combattuta dalle armate italiane in Russia durante la prima guerra mondiale
- B) la guerra totale al terrorismo internazionale promossa da George W. Bush
- C) la guerra commerciale tra i Paesi scandinavi
- D) la contrapposizione tra il blocco filoamericano e quello filosovietico dopo il 1945
- E) un lungo conflitto svoltosi nei Paesi nordici alla fine dell'Ottocento

22. Di quale autore si dice che abbia "sciacquato i panni in Arno"?
- A) Italo Calvino
- B) Alessandro Manzoni
- C) Dante Alighieri
- D) Italo Svevo
- E) Giosue Carducci

Test di Biologia

23. La generazione di due figli aploidi è il risultato:
- A) della mitosi
- B) della metafase
- C) della meiosi
- D) della micosi
- E) della telofase

24. Un processo patologico che alteri il controllo della concentrazione di proteine ed elettroliti fra l'interno e l'esterno della cellula verosimilmente potrebbe essere localizzato:
- A) nel tubulo renale
- B) nei mitocondri
- C) nella membrana cellulare
- D) nei lisosomi
- E) nell'apparato di Golgi

25. Gli esoni sono:
A) organelli citoplasmatici
B) regioni specializzate di proteine
C) sequenze di DNA non codificanti
D) RNA non codificanti
E) sequenze di DNA codificanti

26. I prodotti della digestione sono assorbiti principalmente a livello:
A) del colon e selezionati secondo la funzione
B) dell'intestino tenue e immessi in parte nei capillari e in parte nei vasi linfatici
C) del colon e immessi in parte nei capillari e in parte nei vasi linfatici
D) dello stomaco le proteine, dell'intestino tenue i lipidi e del crasso i glucidi
E) dell'intestino crasso e immessi nella circolazione linfatica ed ematica

27. A seguito del riconoscimento dell'antigene, un linfocita T helper:
A) prolifera
B) produce anticorpi
C) dedifferenzia
D) extravasa
E) degranula

28. La cellulosa è:
A) un polisaccaride con funzione di riserva energetica
B) una catena di peptidi
C) un polimero del β -glucosio
D) un monosaccaride con funzione strutturale
E) un polimero dell' α -glucosio

29. Nel corso della gametogenesi, i cromosomi vanno incontro a:
A) due divisioni senza nessuna replicazione
B) una divisione e due interfasi
C) due replicazioni e una divisione
D) una replicazione e due divisioni
E) due replicazioni e due divisioni

30. Il lisosoma è un vacuolo citoplasmatico:
A) contenente enzimi idrolitici
B) in cui si svolge la maggior parte delle reazioni cataboliche del metabolismo cellulare
C) contenente sostanze di riserva
D) contenente enzimi ossidativi che demoliscono il perossido di idrogeno
E) in cui si svolge la sintesi proteica

31. Le fasi del ciclo cellulare sono, nell'ordine:
A) $S - G_1 - G_2 - M$
B) $G_1 - S - G_2 - M$
C) $G_1 - M - S - G_2$
D) $G_1 - G_2 - S - M$
E) $M - S - G_1 - G_2$

32. La conquista delle terre emerse è stata realizzata da:
A) nessuna delle altre alternative è corretta
B) ciclostomi
C) anfibi
D) pesci
E) alghe verdi-azzurre

33. Nell'evoluzione convergente:
A) le specie si diversificano nel tempo
B) popolazioni diverse tendono ad assomigliarsi, anche se sono imparentate solo alla lontana
C) specie diverse mostrano un mutuo adattamento
D) popolazioni simili e imparentate, se vivono in ambienti separati, si diversificano nel tempo
E) si ha la comparsa di caratteri omologhi

34. Qual è il rischio di generare figli affetti da emofilia quando nella coppia di genitori il padre ne è affetto e la madre è sana?
A) Nessuna delle altre alternative è corretta
B) 25%
C) 100% dei figli maschi
D) 50% dei figli maschi
E) 50%

35. Dove avviene lo splicing dell'RNA?
A) Nei ribosomi
B) Nelle cellule procariotiche
C) Nei cloroplasti
D) Nel nucleo cellulare
E) Nei virus a RNA

36. Citosina e timina sono:
A) nucleotidi
B) basi puriniche
C) amminoacidi
D) zuccheri
E) basi pirimidiniche

37. Quale dei seguenti cariotipi determina la sindrome di Klinefelter?
A) 47, XXY
B) 46, YY
C) 47, XYY
D) 48, XXYY
E) 47, XX + 21

38. I "postulati di Koch" riguardano i criteri per:
A) la classificazione delle malattie causate da microrganismi
B) la determinazione della sensibilità di un microrganismo agli antibiotici
C) riconoscere che un particolare microorganismo sia causa di una particolare malattia
D) l'identificazione di un microrganismo
E) la classificazione di un microorganismo

39. Nei batteri, quale delle seguenti strutture rappresenta un organo per la conservazione della specie?
A) La parete cellulare
B) I flagelli
C) La spora batterica
D) I pili
E) La membrana citoplasmatica

40. Gli acidi grassi si distinguono in saturi, monoinsaturi e polinsaturi a seconda della:
A) presenza di uno o più doppi legami
B) presenza di uno o più atomi di ossigeno
C) presenza di uno o più gruppi carbossilici
D) presenza di acidi grassi essenziali
E) lunghezza dell'acido grasso

Test di Chimica

41. La desinenza -one, secondo le regole internazionali di nomenclatura, spetta:
A) nessuna delle altre alternative è corretta
B) alle ammidi
C) ai sali di acidi a lunga catena
D) ai chetoni
E) agli esteri

42. Quale fra le seguenti è la formula dell'alcool etilico?
A) CH_2OH I CH_2OH
B) CH_3-CH_2-CH_2-CH_2OH
C) CH_3-CH_2-CH_2OH
D) CH_3-CH_2OH
E) CH_3-OH

43. In chimica, la condensazione è il passaggio di stato:
A) da solido a liquido
B) da gassoso a solido
C) da liquido a gassoso
D) da gassoso a liquido
E) da solido a gassoso

44. 18 g di acqua sono necessari per trasformare 56 g di ossido di calcio in idrossido di calcio. Quanti grammi di acqua sono necessari per trasformare 25 g di ossido di calcio?
A) 8,036
B) 9,000
C) 12,000
D) 3,000
E) 3,234

45. Il PH di una soluzione di NaOH 10^{-7} M sarà:

A) tra 6,5 e 7,0
B) tra 9,0 e 9,5
C) tra 5,5 e 6,0
D) tra 8,0 e 8,5
E) tra 7 e 7,5

46. Un liquido bolle:

A) quando la tensione di vapore è zero
B) quando la tensione di vapore eguaglia la pressione atmosferica
C) quando la temperatura oltrepassa i 100 °C
D) quando si formano abbondanti bollicine
E) a 100 °C

47. Due atomi di magnesio che possiedono ugual numero atomico ma numero di massa diverso – rispettivamente 25 e 26 – rappresentano:

A) due elementi con diverso numero di protoni
B) due isotopi del medesimo elemento
C) due elementi con lo stesso numero di neutroni
D) due elementi diversi
E) due isomeri del medesimo elemento

48. Quanto pesa una mole di ferro (numero di massa 56, numero atomico 26)?

A) 56 g
B) 56 mg
C) 82 g
D) 56 kg
E) 26 g

49. Come viene definito il legame che unisce due atomi di idrogeno?

A) Legame allosterico
B) Legame neutro
C) Legame a idrogeno
D) Legame covalente
E) Legame ionico

50. Il massimo numero di ossidazione del fosforo è:

A) 5
B) 4
C) 2
D) –2
E) 3

51. Completare in modo corretto la frase: "L'energia di un orbitale …".

A) è indipendente dal numero quantico secondario
B) diminuisce al crescere del numero quantico principale
C) aumenta al crescere del numero quantico principale
D) non dipende dal valore del numero quantico principale
E) dipende dal numero quantico principale ed è indipendente dal numero quantico secondario

52. Secondo Avogadro, volumi uguali di gas diversi, nelle stesse condizioni di temperatura e di pressione:

A) contengono lo stesso numero di molecole
B) contengono lo stesso numero di atomi
C) contengono lo stesso numero di molecole solo se esse sono monoatomiche
D) contengono lo stesso numero di molecole solo se i gas hanno lo stesso volume molecolare
E) hanno la stessa massa

Test di Fisica e Matematica

53. La resistenza R di un filo conduttore di sezione S e lunghezza l:

A) aumenta all'aumentare di S
B) dipende da l ma non da S
C) diminuisce all'aumentare di l
D) dipende da S ma non da l
E) aumenta all'aumentare di l

54. Qual è la velocità di un camion che percorre un tragitto di 153 km in 1h 30'?

A) 100 km/h
B) 102 km/h
C) 95 km/h
D) 114 km/h
E) 107 km/h

55. Nel Sistema Internazionale, un corpo di massa 10 kg pesa:

A) 98 N
B) 9,8 N
C) 19,6 N
D) 980 N
E) 196 N

56. Per sollevare di 10 metri un ascensore di massa totale uguale a 800 kg, utilizzando un motore di potenza uguale a 8.000 W, saranno approssimativamente necessari:

A) 20 secondi
B) 1 minuto
C) 80 secondi
D) 5 secondi
E) 10 secondi

57. Indicare il minore tra i seguenti numeri: 0,22; 25/100; $21 \cdot 10^{-2}$; 1/5; 1/4.

A) 1/5
B) $21 \cdot 10^{-2}$
C) 1/4
D) 25/100
E) 0,22

58. Qual è la soluzione della disequazione (x – 5)(x – 5) > 0?

A) $-5 < x < 5$
B) $x > 5$
C) $x \neq 5$
D) Nessuna delle altre alternative è corretta
E) Tutti i valori di x

59. L'equazione con valore assoluto |x| = 1 ammette:

A) nessuna soluzione
B) tre soluzioni, $x = -1$, $x = 0$ e $x = 1$
C) una soluzione, $x = -1$
D) due soluzioni, $x = -1$ e $x = 1$
E) una soluzione, $x = 1$

60. Quale, fra i monomi qui sotto elencati, è un minimo comune multiplo dei seguenti monomi: $3p^2 r^4$; $-2q^2r^2$; $5p^2r^2$; $-6q^3r^3$?

A) $p^2q^3r^4$
B) r
C) $30p^2q^3r^4$
D) $30p^2q^2r^2$
E) r^2

********** FINE DELLE DOMANDE **********

PROVA DI AMMISSIONE AI CORSI DI LAUREA DELLE PROFESSIONI SANITARIE

Anno Accademico 2014/2015

Test di Ragionamento logico

1. **Completare correttamente la seguente successione, utilizzando l'alfabeto italiano: E; 9; D; 10; C; 19; B; 29; ?; ?**
   - A) D; 57
   - B) R; 60
   - C) A; 48
   - D) S; 60
   - E) 48; A

2. **Il distributore automatico di un ufficio permette di selezionare tra una vasta gamma di caffè a partire da due miscele: caffè normale o caffè decaffeinato. Il distributore propone caffè di due tipi: ristretto o macchiato con uno di tre tipi di latte (intero, parzialmente scremato, scremato); e, per coloro che lo vogliono zuccherato, permette di selezionare l'aggiunta automatica di uno o due cucchiaini di zucchero oppure uno o due cucchiaini di dolcificante.**
   Quanti tipi diversi di caffè è possibile selezionare nel distributore automatico?
   - A) 48
   - B) 32
   - C) 40
   - D) 11
   - E) 24

3. **Se:**
   $\Delta - 2 \cdot @ = \# + 2 \cdot @$
   $\# = 2 \cdot @$
   $\Delta = 3$
   allora # è uguale a:
   - A) 1
   - B) 0
   - C) 3
   - D) 2
   - E) non ci sono elementi sufficienti per rispondere

4. **Quanto tempo si impiega per contare fino a 10.000, se si conta alla velocità di un numero al secondo?**
   - A) Tra le 3 e le 4 ore
   - B) Più di 24 ore
   - C) Meno di 2 ore
   - D) Tra le 2 e le 3 ore
   - E) Più di 4 ore

5. Posti A = {27, 28, 29} e B = {28, 29, 30}, qual è l'insieme risultante dall'unione di A e B?

A) {28, 29}
B) {27, 28, 29, 30}
C) {27, 28, 28, 29, 29, 30}
D) {28, 29, 30}
E) Un insieme vuoto

6. La piccola Fiorella sta giocando con 378 tessere quadrate di legno colorato, tutte delle stesse dimensioni. Costruisce con le tessere, affiancandole, il più grande quadrato possibile. Considerando il lato di ogni tessera come unità di misura u, quanto vale il perimetro del quadrato ottenuto?

A) 266 u
B) 126 u
C) 72 u
D) 80 u
E) 76 u

7. Si consideri la seguente affermazione: "Ogni numero pari è somma di due numeri primi". Qual è la sua negazione logica?

A) Esistono alcuni numeri primi aventi somma pari
B) La somma di due numeri primi è sempre pari
C) Non tutte le somme di due numeri primi sono pari
D) Nessun numero pari è somma di due numeri primi
E) Esiste almeno un numero pari che non si possa esprimere come somma di due numeri primi

8. Un paio di pantaloni costava 64 euro e con i saldi il suo prezzo attuale è pari ai 3/4 del prezzo iniziale. A quanto ammonta lo sconto applicato?

A) 16 euro
B) 48 euro
C) 20 euro
D) 12 euro
E) 20%

9. Se in una certa zona la piovosità media è di 360 millimetri all'anno, quanto piove mediamente in due anni e cinque mesi?

A) 900 millimetri
B) 720 millimetri
C) 870 millimetri
D) 270 millimetri
E) 570 millimetri

10. Quali tra i termini proposti completano correttamente la seguente proporzione?
Virus : epidemia = X : Y

A) X = vento Y = mulino
B) X = scintilla Y = incendio
C) X = contagio Y = guarigione
D) X = fiammifero Y = accendino
E) X = maremoto Y = terremoto

11. "Se nevica, Arianna accende il camino". Se la precedente affermazione è vera, allora è sicuramente vero che:

A) non nevica e quindi Arianna tiene spento il camino
B) se Arianna accende il camino, vuol dire che nevica
C) se non nevica Arianna non accende il camino
D) se Arianna non accende il camino, vuol dire che non nevica
E) Arianna non accende il camino quando c'è il sole

12. In figura sono rappresentate due bilance a due piatti in equilibrio con alcuni oggetti (sfere, cubi e piramidi).

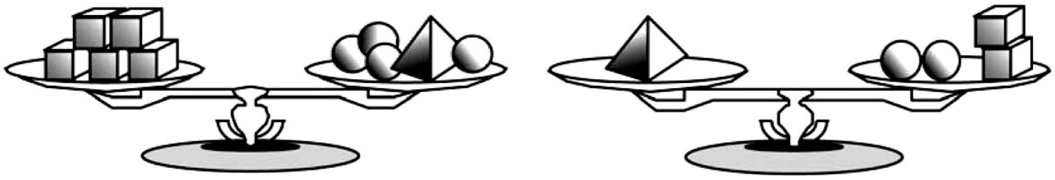

A quanti cubi corrisponde la massa di una piramide?

A) 2
B) 6
C) 5
D) 3
E) 4

13. Individuare il numero mancante.

70	58	39
39	22	15
32	37	?

A) 25
B) 24
C) 23
D) 26
E) 27

Brano 1

Leggere il testo del seguente problema.

Cinque coppie di amici (i coniugi Bresso, Elmi, Gallo, Ornati e Pellini) hanno trascorso una vacanza di una settimana su un'isola caraibica. Le coppie hanno organizzato attività comuni tutti i giorni tranne uno, il penultimo, in cui ogni coppia ha fatto qualcosa di diverso rispetto agli altri, scegliendo tra le seguenti attività (non necessariamente in quest'ordine): visita alla capitale dell'isola, immersione sulla barriera corallina, partita a golf, escursione nella foresta, gita a pesca. Ogni coppia ha inoltre acquistato un souvenir differente, scegliendo tra: una statuetta di legno, il modellino di una nave, una T-shirt, un poster con una foto della spiaggia più famosa dell'isola, una confezione di dolcetti tipici (non necessariamente in quest'ordine). Si sa che:

1) La coppia che è andata a fare un'immersione sulla barriera corallina non ha acquistato i dolcetti tipici del posto;

2) La coppia che è andata a pesca poi ha acquistato un poster con la foto della spiaggia più famosa dell'isola da regalare al nipotino;

3) La coppia che ha visitato la capitale dell'isola ha acquistato una bellissima statuetta di legno che, insieme al modellino di nave scelto dalla coppia che è andata a fare immersione sulla barriera corallina, è stato votato miglior souvenir da tutti gli amici;

4) I coniugi Pellini non sono andati a fare un'escursione nella foresta;

5) Il signor Bresso è rimasto deluso dal suo risultato a golf. Il signor Elmi avrebbe voluto seguire i coniugi Gallo nella visita della capitale dell'isola, ma alla fine ha preferito andare a pesca con la moglie;

6) Il signor Pellini ha apprezzato molto la T-shirt acquistata dai Bresso.

14. Facendo riferimento soltanto ai primi quattro punti del *brano 1*, che cosa si può dedurre con certezza?

A) La statuetta è stata acquistata da coniugi Pellini
B) La famiglia Bresso non ha certamente acquistato i dolcetti
C) La T-shirt potrebbe essere stata acquistata dalla coppia che ha giocato a golf
D) I coniugi Pellini hanno acquistato o la T-shirt o i dolcetti tipici
E) La T-shirt potrebbe essere stata acquistata dalla coppia che ha visitato la capitale

15. Facendo riferimento al *brano 1*, quale dei seguenti abbinamenti è corretto?

A) Elmi – visita alla capitale – statuetta di legno
B) Pellini – escursione nella foresta – T-shirt
C) Elmi – pesca – dolcetti tipici
D) Gallo – visita alla capitale – modellino di nave
E) Pellini – immersione sulla barriera corallina – modellino di nave

16. Facendo riferimento al *brano 1*, quale delle seguenti affermazioni è corretta?

A) I coniugi Gallo hanno acquistato un modellino di nave
B) La famiglia Pellini ha comprato una T-shirt
C) Nessuna delle altre alternative è corretta
D) I coniugi Elmi hanno acquistato un poster di ritorno dall'escursione nella foresta
E) I coniugi Elmi hanno comprato una statuetta di legno

17. Facendo riferimento al *brano 1*, quale delle seguenti affermazioni è FALSA?

A) I coniugi Pellini non hanno acquistato né una statuetta di legno né una T-shirt
B) I coniugi Elmi hanno acquistato un modellino di nave
C) I dolcetti tipici sono stati scelti come souvenir dai coniugi Ornati
D) Chi ha scelto l'escursione nella foresta non ha acquistato né una T-shirt né un poster
E) La coppia che ha fatto un'immersione nella barriera corallina ha acquistato un modellino di nave

18. **Facendo riferimento al *brano 1*, che cosa hanno acquistato i coniugi Gallo?**
- A) La statuetta di legno
- B) La T-shirt
- C) Il modellino di nave
- D) Il poster
- E) I dolcetti tipici

Brano 2

Leggere il brano e rispondere ad ogni quesito collegato solo in base alle informazioni contenute.

Le ricette realizzate dagli ormai famosissimi chef televisivi saranno pure gustose e bellissime da vedersi, ma risultano piene di grassi, calorie, sale e zuccheri in modo maggiore rispetto ai piatti pronti del supermercato, di quelli da tirare fuori dal congelatore e da mettere direttamente nel microonde o in padella. I dati che hanno portato a queste conclusioni arrivano da un articolo pubblicato sul sito del "British Medical Journal".

Già studi precedenti avevano suggerito che sia i pasti pronti che si acquistano al supermercato sia le ricette degli chef televisivi hanno il potere di influenzare la dieta delle persone, ma nessuno studio finora ne aveva esaminato nel dettaglio le caratteristiche nutrizionali.

Quindi, un team di ricercatori britannici dell'Nhs Tees e della Newcastle University ha analizzato il contenuto nutrizionale delle ricette di quattro chef televisivi e dei piatti pronti di tre diverse marche da supermercato, per un totale di 100 pasti per ogni gruppo. Hanno poi confrontato i dati raccolti con le linee guida sull'alimentazione pubblicate dall'Organizzazione mondiale della sanità (OMS) e dalla Food Standards Agency (FSA), l'autorità responsabile della sicurezza e dell'igiene alimentare nel Regno Unito.

Dal confronto con i dati dell'OMS e dell'FSA è emerso che nessuna ricetta e nessun pasto pronto rispetta pienamente le raccomandazioni impartite dalle due autorità, e che entrambi i tipi di pasti tendevano a essere ad alto contenuto di proteine, grassi, grassi saturi e sale, a basso contenuto di carboidrati e nei limiti indicati per quanto riguarda lo zucchero. In particolare, poi, i piatti degli chef del piccolo schermo sono risultati meno sani dei piatti pronti, in quanto contenevano più calorie, proteine, grassi e grassi saturi e molte meno fibre rispetto ai piatti pronti (il 4% dei quali sono risultati conformi alle raccomandazioni dell'OMS). «Questo studio dimostra che né le ricette create dagli chef televisivi, né i piatti pronti prodotti dalle tre principali catene di supermercati britannici sono in grado di soddisfare gli standard nutrizionali nazionali e internazionali per una dieta equilibrata – spiegano gli autori –. Inoltre, le ricette tv sembrano meno sane rispetto ai piatti pronti».

(da: Miriam Cesta, *"Attenti agli chef in tv: ricette meno salutari dei piatti pronti del supermercato"*, Il Sole 24 Ore)

19. **Le seguenti sostanze risultano essere presenti in quantità troppo elevate nei piatti realizzati da famosi chef televisivi, tranne una. Quale? (*vedi brano 2)***
- A) Grassi saturi
- B) Proteine
- C) Sale
- D) Carboidrati
- E) Nessuna delle altre alternative è corretta

20. Da quanto riportato nel *brano 2*, quale dei seguenti dati NON è corretto?

A) Alcuni dei piatti pronti presi in esame sono perfettamente in linea con quanto suggerito dall'OMS e dalla FSA
B) Le ricette realizzate in tv hanno sicuramente un aspetto più invitante dei piatti pronti del supermercato
C) Ci sono persone che variano la loro dieta in funzione dei piatti pronti della grande distribuzione e delle ricette che imparano in tv
D) La ricerca di cui parla il brano non è stata condotta negli Stati Uniti
E) La FSA opera solo in ambito nazionale per quanto riguarda la sicurezza degli alimenti

21. Quale tra le seguenti affermazioni NON è deducibile dal *brano 2*?

A) Una piccola percentuale dei piatti pronti esaminati dallo studio è in linea con le raccomandazioni dell'OMS
B) Sono già diversi gli studi che hanno preso in esame le sostanze nutritive tipiche dei pasti pronti
C) Se si ha bisogno di assumere più fibre, bisogna evitare i piatti proposti in tv dagli chef
D) Per diverse ragioni, i piatti proposti in tv sembrano essere addirittura meno sani di quelli pronti
E) La ricerca ha analizzato le ricette proposte da quattro chef televisivi e i piatti pronti di tre marche da supermercato

22. Qual è il consiglio che deriva dai risultati dello studio descritto nel *brano 2*?

A) I più famosi chef della televisione contestano le più accreditate linee guida sull'alimentazione
B) Cucinate almeno una volta la settimana un piatto proposto dagli chef in tv
C) Evitate la tv e il supermercato
D) Cercate di variare la vostra dieta alternando piatti pronti e piatti cucinati in casa, prendendo spunto il più possibile dai famosi chef della televisione
E) Cucinate in casa e ricorrete in rari casi ai piatti pronti del supermercato, mai o quasi mai a quelli proposti in tv

23. La ricerca di cui si parla nel *brano 2* è stata condotta da ricercatori dell'Università di Newcastle e del Nhs Tees:

A) e ha analizzato i pasti di 700 persone
B) e ha analizzato 100 pasti per ogni gruppo
C) in collaborazione con l'OMS e la FSA, e ha analizzato 100 pasti per ogni gruppo
D) in collaborazione con l'OMS e la FSA, e ha analizzato i pasti di 100 persone
E) in collaborazione con tre catene di supermercati, alcuni chef televisivi, l'OMS e la FSA

Test di Cultura Generale

24. Secondo la Costituzione italiana, il diritto alla salute è:

A) un diritto dei cittadini
B) un diritto fondamentale dell'individuo e un dovere della collettività
C) un'esigenza di sicurezza pubblica
D) un diritto fondamentale della persona e un interesse della collettività
E) un diritto riconosciuto solo agli indigenti

25. Quale tra i seguenti Stati sudamericani è il più meridionale?
A) Brasile
B) Paraguay
C) Cile
D) Uruguay
E) Colombia

26. Quale delle seguenti alternative riporta gli eventi nel corretto ordine cronologico?
A) Affare Dreyfus, Rivoluzione francese, Conferenza di Yalta, Prima guerra mondiale, Guerra franco-prussiana
B) Guerra franco-prussiana, Affare Dreyfus, Conferenza di Yalta, Prima guerra mondiale, Rivoluzione francese
C) Guerra franco-prussiana, Conferenza di Yalta, Prima guerra mondiale, Rivoluzione francese, Affare Dreyfus
D) Rivoluzione francese, Guerra franco-prussiana, Affare Dreyfus, Prima guerra mondiale, Conferenza di Yalta
E) Rivoluzione francese, Guerra franco-prussiana, Conferenza di Yalta, Prima guerra mondiale, Affare Dreyfus

27. "Agitare un liquido in un recipiente" equivale a:
A) debordare
B) affogare
C) schiumare
D) scialacquare
E) sciabordare

Test di Biologia

28. Nelle cellule eucariotiche animali, il DNA è contenuto:
A) nel nucleo e nei ribosomi
B) in tutti i compartimenti cellulari
C) nel nucleo e nei mitocondri
D) solo nel nucleo
E) nel nucleo e nei perossisomi

29. I virus sono:
A) cellule eucariote
B) cellule procariote
C) commensali
D) particelle submicroscopiche bioparassite
E) parassiti facoltativi

30. Gli eritrociti sono:
A) globuli rossi
B) nessuna delle altre risposte è corretta
C) macrofagi
D) monociti
E) globuli rossi e globuli bianchi

31. Il cardias è:
- A) l’orifizio che congiunge esofago e stomaco
- B) il canale attraverso cui l'esofago si congiunge con lo stomaco
- C) il muscolo cardiaco
- D) l’orifizio che congiunge stomaco e duodeno
- E) una membrana che avvolge il ventricolo sinistro del cuore

32. Il sistema nervoso autonomo:
- A) si attiva solo in situazioni di pericolo
- B) è controllato dalla volontà
- C) innerva la muscolatura scheletrica
- D) contribuisce a mantenere l’omeostasi dell’organismo
- E) fa parte del sistema nervoso centrale

33. Durante la contrazione dei sarcomeri:
- A) i filamenti sottili di actina si accorciano
- B) le linee Z si allontanano
- C) i filamenti sottili di actina scorrono su quelli spessi di miosina
- D) i filamenti di miosina si accorciano e quelli di actina si allungano
- E) i filamenti sottili di miosina scorrono su quelli spessi di actina

34. Una sostanza riconosciuta come estranea all'organismo dal sistema immunitario è:
- A) un anticorpo
- B) un antigene
- C) una citochina
- D) un’immunoglobulina
- E) un interferone

35. Un enzima:
- A) è il prodotto di una reazione chimica
- B) ha una struttura lipidica
- C) accelera la velocità di una reazione chimica
- D) viene consumato durante la reazione chimica
- E) è il reagente di una reazione chimica

36. Le mutazioni sono:
- A) modificazioni casuali di proteine, non trasmissibili
- B) meccanismi con cui le cellule si difendono da eventi potenzialmente letali
- C) modificazioni casuali del materiale genetico, ereditabili dalle cellule figlie
- D) meccanismi di morte programmata delle cellule
- E) modificazioni del materiale genetico, non trasmissibili

37. Nel DNA si formano due legami idrogeno tra le coppie di basi complementari:
- A) citosina e timina
- B) timina e guanina
- C) uracile e timina
- D) adenina e timina
- E) guanina e citosina

38. Che differenza c'è tra un nucleo aploide e un nucleo diploide?

A) Il nucleo aploide presenta corredo cromosomico n, quello diploide presenta corredo cromosomico 2n
B) Il nucleo aploide presenta corredo cromosomico 2n, quello diploide presenta corredo cromosomico n
C) Il nucleo aploide ha n cromosomi e quello diploide 2n + 1 cromosomi
D) Il nucleo aploide ha n cromosomi e quello diploide non ha cromosomi
E) Nessuna delle altre risposte è corretta

39. L'adenosina trifosfato (ATP) è:

A) il mediatore di alcuni ormoni
B) un costituente del DNA
C) una vitamina
D) un fosfolipide
E) la molecola utilizzata come moneta di scambio energetico

40. Durante la sistole ventricolare sono:

A) aperte le valvole tra ventricoli e arterie
B) chiuse le valvole tra ventricoli e arterie
C) chiuse le valvole tra vene e atri
D) aperte le valvole tra atri e ventricoli
E) aperte le valvole tra atri e arterie

41. L'allele è:

A) nessuna delle altre alternative è corretta
B) un costituente della membrana plasmatica
C) una cellula con filamenti
D) la forma alternativa di un gene
E) la forma alternativa di una proteina

42. Una coppia ha avuto due figli maschi. Qual è la probabilità che il terzo figlio sia una femmina?

A) 25%
B) 37%
C) 50%
D) 12,50%
E) 100%

Test di Chimica

43. Il solfito acido di sodio corrisponde a:

A) $NaSO_3$
B) $NaHSO_4$
C) Na_2SO_3
D) $NaHSO_3$
E) NaHS

44. Si definiscono isotopi:
- A) atomi con uguale numero atomico e diverso numero di elettroni
- B) atomi con uguale numero atomico e uguale numero di massa
- C) atomi con uguale numero atomico e diverso numero di massa
- D) atomi con diverso numero atomico e uguale numero di elettroni
- E) atomi con diverso numero atomico e uguale numero di massa

45. L'ossido di potassio, posto in acqua, forma:
- A) un sale
- B) una soluzione acida
- C) una soluzione basica
- D) potassio libero
- E) idruro di potassio

46. Per decomposizione termica del nitrato di sodio si ottengono sia nitrito di sodio solido sia ossigeno molecolare gassoso: $NaNO_3 \rightarrow NaNO_2 + O_2$. Individuare la reazione bilanciata:
- A) $2NaNO_3 \rightarrow 2NaNO_2 + O_2$
- B) $3NaNO \rightarrow 3NaNO_2 + O_2$
- C) $2NaNO_3 \rightarrow 2NaNO_2 + 3/2O_2$
- D) $NaNO_3 \rightarrow NaNO_2 + O_2$
- E) $2NaNO_3 \rightarrow 2NaNO_2 + 3O_2$

47. Una soluzione 0,1 molare si prepara sciogliendo 0,1 moli di soluto in:
- A) un chilo di solvente
- B) 100 millilitri di solvente
- C) un litro di solvente
- D) un litro di soluzione
- E) un chilo di soluzione

48. Un atomo ha numero di massa 18 e numero atomico 8; i neutroni contenuti nel suo nucleo sono:
- A) 18
- B) 8
- C) 16
- D) 10
- E) 23

49. Il calcio è un:
- A) metallo di transizione
- B) metallo alcalino terroso
- C) metallo alcalino
- D) metallo terroso
- E) non metallo

50. Lo ione negativo di un atomo deriva:
- A) dall’acquisto di uno o più elettroni
- B) dalla perdita di un protone
- C) dalla perdita di un neutrone
- D) dall’acquisto di un neutrone e di un elettrone
- E) dalla trasformazione di un protone in elettrone

51. Il numero di Avogadro indica:
A) il numero di molecole presenti in una mole
B) il numero di molecole presenti in un litro d'acqua
C) un numero di grammi uguale al peso atomico o molecolare
D) il numero di atomi presenti in 1 g di carbonio
E) il numero di grammi di ossigeno presenti in una mole

52. I trigliceridi sono:
A) alcoli trivalenti
B) acidi carbossilici
C) alcoli terziari
D) idrocarburi
E) lipidi

Test di Fisica e Matematica

53. Le renne di Babbo Natale compiono un lavoro pari a 25.000 J per trascinare la slitta su di una distesa pianeggiante esercitando una forza di 100 N; qual è la distanza coperta dalla slitta?
A) 350 m
B) 250 m
C) 2.500 m
D) 25 m
E) 3.500 m

54. Quanto vale il modulo della somma di due vettori antiparalleli se i loro moduli valgono rispettivamente 3 e 21?
A) 3
B) 18
C) 21
D) 24
E) 63

55. Quale delle seguenti grandezze ha per unità di misura il joule (J)?
A) Quantità di moto
B) Forza
C) Energia
D) Induttanza
E) Potenza

56. Come si chiama in fisica il movimento di un corpo che percorre una traiettoria a velocità costante?
A) Rettilineo
B) Circolare
C) Parabolico
D) Uniforme
E) Armonico

57. Qual è l'equazione della parabola di vertice V = (1, 0) e passante per A = (0, 3)?

A) $y = x^2 - 3x + 3$
B) $y = x^2 + 6x + 2$
C) $y = 3x^2 - 6x + 3$
D) $y = 3x^2 + 6x + 3$
E) $y = 3x^2 - 6x - 3$

58. In un triangolo rettangolo un cateto misura 6 dm e la differenza tra l'ipotenusa e questo cateto è pari a 4 dm. Quanto misura l'altro cateto del triangolo?

A) 15 dm
B) 6 dm
C) 8 dm
D) 14,39 dm
E) 800 cm

59. Le soluzioni dell'equazione $x^2 - 9x + 14 = 0$ sono:

A) $x = 7$ e $x = 2$
B) $x = 7$ e $x = 1$
C) $x = -7$ e $x = -2$
D) $x = -7$ e $x = 2$
E) $x = 7$ e $x = -2$

60. I grafici delle funzioni $y = -x^2$ e $y = x^2$:

A) non hanno intersezioni
B) si sovrappongono
C) si intersecano in 2 punti distinti
D) hanno una sola intersezione
E) nessuna delle altre alternative è corretta

********** FINE DELLE DOMANDE **********

PROVA DI AMMISSIONE AI CORSI DI LAUREA DELLE PROFESSIONI SANITARIE

Anno Accademico 2013/2014

Test di Logica e Cultura generale

1. In base alla Costituzione italiana, i delegati delle Regioni che partecipano all'elezione del Presidente della Repubblica sono eletti:

A) dal popolo
B) dalle Giunte regionali
C) dai Consigli regionali
D) dai Presidenti delle Giunte regionali
E) da ciascuna camera del Parlamento

2. Qual è la capitale della Turchia?

A) Nicosia
B) Istanbul
C) Smirne
D) La Valletta
E) Ankara

3. Quale figura retorica esprime "la parte per il tutto"?

A) Metafora
B) Chiasmo
C) Iperbole
D) Anadiplosi
E) Sineddoche

4. Completare correttamente la seguente successione numerica: 101; 104; 79; 65; 68; 43; ?; ?

A) 29; 32
B) 29; 4
C) 46; 32
D) 18; 21
E) 29; 42

5. Quali tra i termini proposti completano correttamente la seguente proporzione?
lunedì : X = mercoledì : Y

A) X = martedì; Y = venerdì
B) X = primo; Y = quarto
C) X = domenica; Y = martedì
D) X = sabato; Y = martedì
E) X = giovedì; Y = lunedì

6. Una biblioteca contiene 160 libri così suddivisi per materia: biologia 20%; medicina 30%; letteratura 35%; chimica 5%; storia 10%. I libri di quali materie, tra loro sommati, sono 48?
A) Quelli di biologia e quelli di letteratura
B) Quelli di medicina e quelli di storia
C) Quelli di biologia e quelli di storia
D) Quelli di letteratura e quelli di storia
E) Quelli di biologia e quelli di chimica

7. Si dispone di una bilancia a due piatti con il braccio sinistro che misura il triplo del braccio destro. Se nel piatto destro vengono posti 36 pesi tutti uguali fra loro, quanti pesi dello stesso tipo devono essere posizionati nel piatto sinistro affinché la bilancia risulti in equilibrio?
A) 7
B) 16
C) 8
D) 18
E) 12

8. Una regola di elaborazione trasforma l'ottupla (1, 4, 6, 3, 9, 7, 8, 5) in (4, 1, 6, 3, 9, 7, 8, 5) e questa in (4, 6, 1, 3, 9, 7, 8, 5). Individuare l'ottupla successiva secondo la stessa regola.
A) (4, 6, 3, 1, 9, 7, 8, 5)
B) (4, 6, 1, 3, 9, 7, 8, 5)
C) (4, 6, 1, 3, 7, 9, 8, 5)
D) (4, 6, 1, 9, 3, 7, 8, 5)
E) (4, 6, 3, 1, 7, 9, 8, 5)

9. Cinque treni, A, B, C, D ed E, partono dalla stessa stazione diretti in cinque città differenti. Si sa che:
i) C è più veloce di B ma arriva dopo di questo che è, invece, il primo ad arrivare;
ii) A è l'ultimo ad arrivare anche se è più veloce di D e meno veloce di B;
iii) E, il secondo treno ad arrivare a destinazione, è più veloce di D, ma meno veloce di A.
In base alle precedenti informazioni il quinto treno più lento è:
A) A
B) C
C) D
D) B
E) non è possibile determinarlo

10. La piccola Aurelia sta giocando con 985 tessere di legno colorato, tutte a forma di triangolo equilatero e aventi le stesse dimensioni. Ha costruito con esse, affiancandole, il triangolo equilatero più grande possibile; quante tessere sono avanzate ad Aurelia?
A) 25
B) 24
C) 23
D) 31
E) 26

11. Quale dei seguenti avvenimenti si colloca cronologicamente tra la Rivoluzione francese e l'Unità d'Italia?

A) La guerra di Crimea
B) La Rivoluzione americana
C) L'avvento del Terzo Reich
D) La Rivoluzione inglese
E) La firma dei Patti Lateranensi

12. Individuare tra i seguenti termini il sinonimo di "ablazione":

A) aspettativa
B) offerta
C) asportazione
D) furto
E) ovazione

Brano 1

Leggere il brano e rispondere a ogni quesito solo in base alle informazioni contenute (esplicitamente o implicitamente) nel brano e non in base a quanto il candidato eventualmente conosca sull'argomento.

Le vicende alimentari dell'Europa del Settecento sembrano ripercorrere cammini noti: espansione demografica, insufficienze produttive, sviluppo agricolo. Una storia che ricorda da vicino quella dei secoli XI-XII, o del XVI. Solo che questa volta le dimensioni del fenomeno sono ingigantite. La popolazione europea, che aveva raggiunto a metà del Trecento una punta di forse 90 milioni di individui, e che attorno al 1700 (dopo la grande crisi e la successiva lenta ripresa) era attestata sui 125 milioni, cresce da allora in poi rapidissimamente: 145 milioni a metà del XVIII secolo, 195 alla fine. Il sistema produttivo è messo a dura prova, le carestie si abbattono a intervalli regolari sulla popolazione. Alcune di esse (tristemente celebre quella del 1709-10) colpiscono l'intera Europa, dalla Spagna all'Italia, dalla Francia all'Inghilterra, dalla Germania alla Svezia, ai Paesi dell'Est. Altre investono territori più circoscritti: quella del 1739-41 colpì soprattutto Francia e Germania; quella del 1741-43 l'Inghilterra; quella del 1764-67 fu particolarmente grave nelle regioni meridionali (Spagna, Italia); quella del 1771-74 nei Paesi del Nord. Nell'insieme, gli anni "difficili" del XVIII secolo sembrano numerosi come non mai (a eccezione forse che nell'XI secolo). Ciò non vuol dire che la gente muoia di fame: se così fosse stato, l'exploit demografico sarebbe a dir poco incomprensibile. Siamo invece di fronte a un malessere diffuso, a uno stato di sottonutrizione permanente che viene per così dire "assimilato" (fisiologicamente e culturalmente) come condizione normale di vita.

All'aumentata richiesta di cibo si rispose, per cominciare, nel modo più semplice e tradizionale: l'espansione dei coltivi. In Francia, nei decenni precedenti la Rivoluzione, le terre a coltura passarono da 19 a 24 milioni di ettari nel giro di trent'anni. In Inghilterra, nella seconda metà del secolo, furono recintati e messi a coltura centinaia di migliaia di ettari di terreni incolti e boschivi. In Irlanda, in Germania, in Italia si prosciugarono paludi e acquitrini. Contemporaneamente si misero a punto nuove tecniche produttive, in un clima di fervore scientifico e di sperimentazione agronomica che per la prima volta riuscì a incontrarsi con gli interessi imprenditoriali dei proprietari terrieri. Si parla a ragione, per quest'epoca, di una vera rivoluzione agricola: tale fu, dal punto di vista tecnico, l'abbandono della pratica del maggese e l'impiego delle leguminose da foraggio in regolare rotazione con i cereali. Ciò consentì, da un lato, di integrare le pratiche zootecniche nel sistema agrario, superando la tradizionale separazione fra attività pastorali e attività agricole; dall'altro, di accrescere sensibilmente i rendimenti del suolo, reso più fertile sia dalla presenza delle leguminose (che possiedono la proprietà di fissare l'azoto nel terreno) sia dalla maggiore disponibilità di concime animale.

Queste e altre trasformazioni segnarono l'avvio del capitalismo agrario, che in certe regioni europee – soprattutto l'Inghilterra e poi la Francia – fu il primo passo verso l'affermarsi dell'economia industriale. All'ampliamento dei terreni coltivati e al perfezionamento delle tecniche produttive si affiancò lo sviluppo di colture particolarmente robuste, sicure e redditizie: quelle stesse che avevano trovato una prima timida diffusione (in ambiti localmente limitati) fra Quattro e Cinquecento, e che vengono ora "riscoperte" come soluzione a basso costo di pressanti esigenze alimentari. Il riso, dopo un certo declino nel XVII secolo, legato anche alle polemiche sull'opportunità igienica e ambientale di far ristagnare l'acqua nei campi, torna in auge nel Settecento come alternativa ai cereali tradizionali: in certe zone esso viene introdotto per la prima volta; in altre viene per così dire reintrodotto. Analoga destinazione sociale ha il grano saraceno, anch'esso "riscoperto" nel Settecento oppure, in certe regioni,

introdotto per la prima volta.

Ma sono soprattutto il mais e la patata a conquistare un ruolo di assoluto primo piano, sbarazzando il campo da molti antichi concorrenti: fra XVIII e XIX secolo, la tradizionale varietà di cereali inferiori – base millenaria della dieta popolare – viene progressivamente riducendosi a favore dei nuovi protagonisti.

13. Quale tra le seguenti affermazioni è ricavabile dal *brano 1*?
A) I proprietari terrieri non furono disposti a rischiare i loro capitali
B) Solo in Inghilterra e in Francia si sperimentarono nuovi sistemi agrari
C) Le nuove tecniche agrarie si avvalsero dell'apporto della scienza del Settecento
D) L'estensione dei boschi e delle paludi ostacolò il progresso agricolo
E) L'espansione demografica dell'Europa nel Settecento è stata possibile solo grazie all'aumento delle superfici destinate a coltura

14. Secondo il *brano 1*, quali prodotti furono riscoperti nel Settecento?
A) Il farro e la segale
B) Il mais e la patata
C) I cereali inferiori
D) Il riso e il grano saraceno
E) Le leguminose

15. Qual è, a giudizio dell'autore del *brano 1*, il tratto distintivo dell'espansione storica vissuta dall'Europa del Settecento, considerato che egli stesso attribuisce un carattere ricorsivo alle vicende narrate?
A) La portata, ovvero le dimensioni dei cambiamenti in essere
B) Il fatto che nel Settecento la gente muoia di fame
C) Il fatto che i cambiamenti furono per molto tempo limitati a Francia e Inghilterra
D) Il numero di anni "difficili", senza uguali nei secoli precedenti
E) La totale assenza di carestie, che diede forte impulso alla manodopera agricola

16. Secondo quanto affermato nel *brano 1*, quali fattori furono alla base della rivoluzione agricola del XVIII secolo?
A) L'interesse dei governanti verso l'agricoltura
B) L'abbandono del maggese e la diminuzione dell'allevamento
C) L'introduzione della rotazione triennale e dell'aratro pesante
D) Il diffuso malessere per uno stato di permanente denutrizione
E) L'introduzione delle leguminose da foraggio e l'integrazione tra allevamento e sistema agrario

17. Da quanto riportato nel *brano 1*, si può dedurre che:
A) si accentuò la separazione tra allevamento e agricoltura
B) l'economia industriale ricevette un impulso dal capitalismo agrario
C) il commercio dei cereali favorì l'accumulo di capitali
D) il riso e il grano saraceno sono le colture più robuste, sicure e redditizie tra quelle “riscoperte” nel Settecento
E) la popolazione europea superò i 100 milioni nel corso del XIV secolo

Brano 2

Leggere il testo del seguente problema.
Al primo anno del corso di laurea in Lingue, sei compagni di studi sono chiamati a scegliere ciascuno due corsi opzionali fra i sei disponibili: inglese, francese, tedesco, spagnolo, cinese, esperanto. È noto che:
1) Arianna e Beatrice hanno litigato, e non desiderano frequentare alcun corso in comune;
2) Chiara è la migliore amica di Arianna: anche lei eviterà accuratamente la compagnia di Beatrice;
3) Damiano ed Eluana sono entrambi iscritti al corso di esperanto, ma hanno fatto scelte diverse per quanto riguarda il secondo corso a cui iscriversi: lui tedesco, lei spagnolo;
4) il cinese ha la fama di essere una lingua difficile: solo Chiara l'ha scelto. L'inglese è stato invece scelto da tre studenti. Tutti gli altri corsi avranno ciascuno due studenti;
5) Beatrice è una studentessa Erasmus proveniente da Bordeaux, quindi non le interessa frequentare il corso di francese. Anche Frank è uno studente Erasmus, proveniente da Berlino, ma questo non influirà sulla sua scelta di frequentare o meno il corso di tedesco.

18. Chi frequenterà il corso di inglese? *(vedi brano 2)*

A) Beatrice, Eluana e Frank
B) Beatrice, Damiano e Frank
C) Beatrice, Damiano ed Eluana
D) Arianna, Chiara e Frank
E) Arianna, Damiano e Frank

19. A metà anno accademico Giovanna, sorella di Beatrice, decide di iscriversi al corso di laurea in Lingue e deve scegliere anch'essa due corsi. Sapendo che Giovanna condivide le amicizie e le inimicizie di sua sorella, le sue possibilità di scelta sono ristrette a: *(vedi brano 2)*

A) tedesco, spagnolo e inglese
B) spagnolo, inglese e tedesco
C) inglese, spagnolo ed esperanto
D) tedesco, spagnolo ed esperanto
E) spagnolo, inglese e francese

20. Oltre al cinese, quale altro corso sarà frequentato da sole ragazze? *(vedi brano 2)*

A) Francese
B) Inglese
C) Tedesco
D) Esperanto
E) Spagnolo

21. Quale sarà o quali saranno i compagni di corso di Beatrice, considerando entrambi i corsi a cui si è iscritta la ragazza? *(vedi brano 2)*

A) Damiano e Frank
B) Solo Damiano
C) Damiano ed Eluana
D) Eluana e Frank
E) Solo Frank

22. **Dopo aver registrato tutte le iscrizioni, la presidentessa del corso di laurea annulla il corso di cinese per lo scarso interesse mostrato dagli studenti e invita Chiara a ripiegare su un altro corso. Tenendo conto dei vincoli espressi, la scelta di Chiara ricadrà:** ***(vedi brano 2)***
A) su francese, tedesco o esperanto
B) su francese, tedesco o spagnolo
C) su francese o esperanto
D) necessariamente sull'esperanto
E) su tedesco, spagnolo o esperanto

23. **Su un pianeta vivono tre persone: Antonio, Marco, Giovanni. Esiste una persona sul pianeta, più ricca di tutte le altre. Marco è più ricco di Antonio. Antonio è più povero di Giovanni. Quale delle seguenti conclusioni è sicuramente FALSA?**
A) Antonio è il più povero
B) Giovanni è il più ricco
C) Giovanni è più povero di Marco
D) Marco è il più ricco
E) Marco e Giovanni hanno la stessa quantità di soldi

24. **Il prodotto delle tre facce visibili del dado in figura dà come risultato 90. Quanto valgono rispettivamente A e B?**

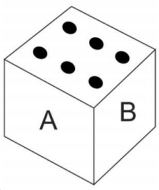

A) 5; 3
B) 4; 2
C) 3; 1
D) 4; 1
E) 5; 4

25. **Sommando la frazione che rappresenta il rapporto fra la parte annerita e l'area totale della figura 1 alla frazione che rappresenta il rapporto fra la parte bianca e l'area totale della figura 2, si ottiene:**

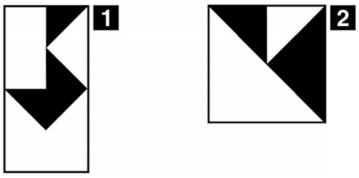

A) 5/8
B) 10/16
C) 6/8
D) 7/8
E) 3/4

26. La figura 1 sta a 72 come la figura 2 sta a:

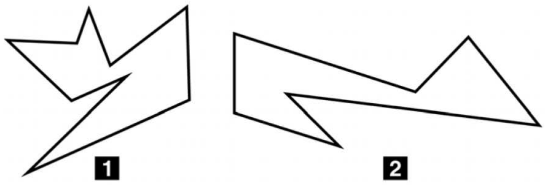

A) 49
B) 56
C) 63
D) 98
E) 48

27. *"Tutte le persone alte sono buone"*. Negare questa affermazione significa affermare con certezza che:

A) nessuna persona bassa è buona
B) almeno una persona bassa non è buona
C) nessuna persona alta è buona
D) almeno una persona alta non è buona
E) tutte le persone basse sono buone

28. Si tenga presente che a segno uguale corrisponde cifra uguale.

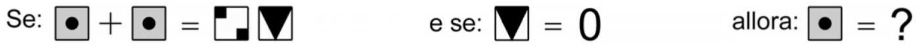

A) 1
B) 7
C) Impossibile determinarlo
D) 3
E) 5

29. Il primo disegno in figura rappresenta la vista laterale di un solido.

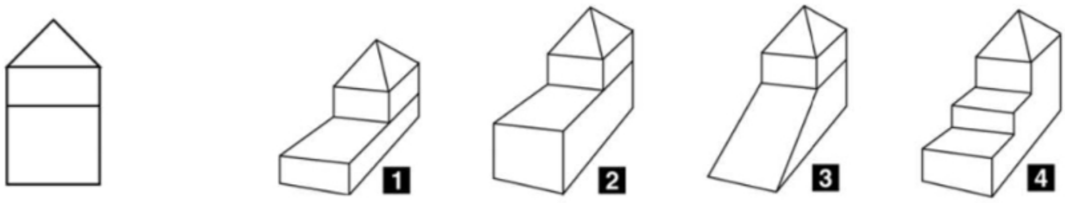

Quale o quali tra i solidi numerati sono compatibili con tale vista?

A) Solo il solido 2
B) Solo il solido 3
C) I solidi 1 e 3
D) Il solido 4
E) I solidi 2 e 3

30. L'affermazione "quando corro a lungo consumo grassi" è equivalente a:

A) se non consumo grassi allora non ho corso a lungo
B) a volte capita che non consumi grassi pur avendo corso a lungo
C) non consumo grassi pur avendo corso a lungo
D) se consumo grassi vuol dire che ho corso a lungo
E) corro a lungo o consumo grassi

Test di Biologia

31. I bronchi:
- A) compongono le ultime vie aeree
- B) fanno parte dell'apparato digerente
- C) fanno parte dell'apparato urinario
- D) non esistono nella specie umana, ma solo nei pesci
- E) il quesito è senza soluzione univoca o corretta

32. Durante il movimento di adduzione, un arto:
- A) si allontana dal corpo
- B) compie movimenti oscillatori
- C) si avvicina al corpo
- D) compie movimenti circolari
- E) effettua una torsione di 90°

33. Quale delle seguenti affermazioni relative al DNA è FALSA?
- A) Alcuni virus contengono DNA
- B) Il DNA è presente sia negli eucarioti sia nei procarioti
- C) Il DNA è formato da nucleotidi
- D) È presente anche nei ribosomi
- E) Il DNA ha una struttura a doppia elica

34. L'emoglobina è una proteina che si trova:
- A) in tutte le cellule eucariote
- B) in tutte le cellule eucariote e procariote
- C) nel plasma
- D) negli eritrociti
- E) solo negli epatociti

35. Nel citoplasma della cellula vegetale si trovano delle cavità chiamate:
- A) vacuoli digestivi
- B) nucleosomi
- C) vacuoli
- D) carioplasmi
- E) nessuna delle altre risposte è corretta

36. Le strutture intestinali deputate all'assorbimento sono i:
- A) microtubuli
- B) microvilli
- C) microfilamenti
- D) filamenti di actina
- E) vasi linfatici

37. Il plasma è:
- A) la parte liquida del sangue
- B) un sinonimo di sangue
- C) la parte corpuscolata del sangue
- D) la parte liquida del sangue che rimane dopo la coagulazione
- E) il sangue senza la componente dei globuli rossi

38. La mitosi è un meccanismo per mezzo del quale:
A) si riproducono i virus, i batteri e gli organismi eucarioti
B) si riproducono i batteri
C) vengono sintetizzati gli zuccheri
D) si riproducono sia gli organismi eucarioti sia gli organismi procarioti
E) una cellula eucariote produce due cellule geneticamente uguali

39. Il crossing-over:
A) non è presente negli insetti
B) favorisce il riassortimento del corredo genetico
C) permette la riproduzione sessuale
D) avviene nella profase della meiosi e della mitosi
E) nessuna delle altre risposte è corretta

40. I virus:
A) sono parassiti endocellulari obbligati
B) sono sempre circondati da membrana
C) sono visibili al microscopio ottico
D) sono in grado di produrre energia
E) sono dotati di nucleo

41. Con il termine "anfigonia" si indica:
A) la riproduzione degli anfibi
B) una malattia delle gonadi
C) la riproduzione che avviene mediante fusione tra gamete maschile e femminile
D) la riproduzione che avviene mediante fusione tra due gameti maschili
E) l'istinto predatorio delle rane

42. Per organismo "diploide" si intende un organismo:
A) in cui trascrizione e traduzione sono accoppiate
B) costituito da due cellule
C) in cui ogni cromosoma è rappresentato due volte
D) con due cromosomi
E) costituito da cellule con due nuclei

43. Quando una molecola di clorofilla è colpita da energia luminosa:
A) tutte le lunghezze d'onda sono assorbite
B) la luce rossa è riflessa
C) tutte le lunghezze d'onda sono riflesse
D) la luce verde è la più assorbita
E) la luce verde non è assorbita

44. Animali la cui temperatura corporea è variabile in rapporto alla temperatura ambientale, sono detti:
A) eterozigoti
B) omeotermi
C) autotrofi
D) il quesito è senza soluzione univoca o corretta
E) eterotrofi

Test di Chimica

45. I due nuclidi ^{14}C e ^{14}N hanno in comune il numero:
A) di massa
B) atomico
C) di neutroni
D) di elettroni
E) di protoni

46. Una soluzione A è ipertonica rispetto a una soluzione B se:
A) la soluzione A si trova a una temperatura maggiore rispetto alla soluzione B
B) la soluzione A è più acida della soluzione B
C) separando le due soluzioni mediante una membrana semipermeabile, si instaura un flusso netto di solvente da A verso B
D) separando le due soluzioni mediante una membrana semipermeabile, si instaura un flusso netto di solvente da B verso A
E) i valori delle proprietà colligative della soluzione B sono maggiori di quelli della soluzione A

47. Attribuire il nome al seguente composto: $Pb(HSO_4)_2$.
A) Solfato di piombo
B) Idrogenosolfito di piombo (II)
C) Bisolfato piombico
D) Solfato acido di piombo (II)
E) Solfito acido piomboso

48. Tutti i legami chimici:
A) sono forze di attrazione fra elettroni e nuclei
B) sono forze di attrazione
C) sono forze di attrazione tra nuclei
D) sono forze di attrazione e di repulsione
E) non sono mai di natura elettrostatica

49. Sapendo che gli elementi Na, Mg, P, S e Cl occupano rispettivamente il I, II, V, VI e VII gruppo e appartengono tutti allo stesso periodo, quale di essi avrà la più bassa energia di ionizzazione?
A) P
B) S
C) Cl
D) Mg
E) Na

50. Un acido è detto poliprotico quando in acqua:
A) è molto dissociato
B) è poco dissociato
C) può cedere più di un protone al solvente
D) forma più ioni H^+
E) è costituito da un polimero

51. Quando, in una reazione di ossido-riduzione, una specie chimica perde elettroni:
A) si ossida, quindi è l'agente riducente perché riduce l'altra specie chimica
B) si riduce, quindi è l'agente ossidante perché ossida l'altra specie chimica
C) si ossida e si riduce contemporaneamente
D) il suo numero di ossidazione diminuisce
E) si ossida, quindi è l'agente ossidante perché ossida l'altra specie chimica

52. Una soluzione con pH = 9 è da considerarsi:
A) debolmente acida
B) acida
C) neutra
D) basica
E) isotonica

Test di Fisica e Matematica

53. ***cos*(*a* + *b*) equivale a:**
A) $1 - sen(a + b)$
B) $2cos(a) \cdot sen(b)$
C) $cos(a) \cdot cos(b) - sen(a) \cdot sen(b)$
D) sen(a – b)
E) $cos(a) \cdot sen(b) + sen(a) \cdot cos(b)$

54. L'unità di misura dell'intensità di corrente è:
A) C/m^2
B) C/s
C) C
D) C/m
E) Cs

55. Una resistenza percorsa da una corrente I = 2 A dissipa per effetto Joule una potenza P = 28 W. Determinare il valore della tensione ai capi della resistenza.
A) 7 V
B) 28 V
C) 14 V
D) Non è possibile rispondere perché non si conosce il valore della resistenza
E) 56 V

56. Si consideri un pattinatore che sta pattinando su una pista perfettamente orizzontale. Il lavoro compiuto dalla forza peso sarà:
A) maggiore di zero
B) nullo
C) minore di zero
D) indeterminato
E) pari a 9,81 J

57. L'energia interna di un gas perfetto è:

A) la somma delle energie cinetiche e potenziali delle diverse molecole
B) inversamente proporzionale alla temperatura
C) la temperatura del gas
D) nessuna delle altre risposte è corretta
E) la somma delle energie cinetiche delle diverse molecole

58. L'equazione $x^2 + 49 = 0$ ha soluzioni:

A) reali
B) $x_1 = x_2 = -7$
C) $x_1 = x_2 = 7$
D) non reali
E) $x_1 = 7$; $x_2 = -7$

59. Sapendo che l'angolo AOB misura 50°, quanto misura l'angolo ABC?

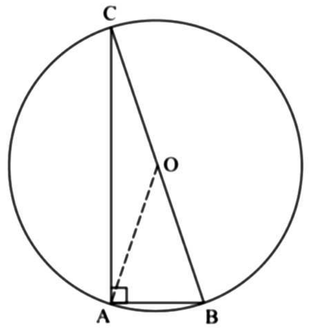

A) 65°
B) 25°
C) 50°
D) 90°
E) 155°

60. Quale delle seguenti equazioni rappresenta la retta passante per l'origine degli assi e per il punto (6;3)?

A) $y = 3x + 3$
B) y = 2x
C) $y = x - 3$
D) y = x
E) $y = (1/2)x$

********** FINE DELLE DOMANDE **********

PROVA DI AMMISSIONE AI CORSI DI LAUREA DELLE PROFESSIONI SANITARIE

Anno Accademico 2012/2013

Test di Logica e Cultura generale

1. Come è definito dalla Costituzione l'esercizio del voto?
- A) Un obbligo politico
- B) Un obbligo civile
- C) Un diritto politico
- D) Un dovere civico
- E) Un diritto civile

2. Il "welfare state" indica:
- A) lo stato sociale che assicura il benessere dei cittadini
- B) il superamento delle discriminazioni razziali
- C) la tutela dei diritti del bambino
- D) la parità dei diritti uomo-donna
- E) il raggiungimento del benessere psicofisico

3. Lo "statuto dei lavoratori" è:
- A) un documento unitario dei sindacati confederali per difendere i diritti dei lavoratori
- B) un protocollo di intesa per la stesura dei contratti
- C) un accordo privato tra azienda e lavoratori
- D) un contratto sindacale
- E) una legge dello Stato italiano

4. Quale tra i seguenti numeri romani indica il valore più alto?
- A) LXXVIII
- B) CCCVII
- C) DCCLXXIV
- D) CCXCV
- E) XXIX

5. Quale dei seguenti codici NON è una raccolta di "norme giuridiche"?
- A) Codice della navigazione
- B) Codice etico
- C) Codice di diritto canonico
- D) Codice di diritto penale
- E) Codice di diritto civile

6. "Glabro" vuol dire:
- A) senza nei
- B) senza complicazioni
- C) senza pudore
- D) senza peli
- E) senza fronzoli

7. Tra i seguenti termini d'ambito medico, qual è l'unico che indica una patologia?
A) Parotide
B) Biopsia
C) Apofisi
D) Paresi
E) Glicemia

8. Qual è l'unica parola che significa "rottura"?
A) Elucubrazione
B) Estrazione
C) Rifrazione
D) Diffrazione
E) Effrazione

9. Il verbo catalizzare significa:
A) analizzare
B) purificare
C) accelerare
D) scaricare
E) decontaminare

10. La rivoluzione industriale inglese, che si profilò nella seconda metà del XVIII secolo, affermò nella prima metà del secolo successivo il protagonismo di una delle seguenti forze motrici:
A) elettricità
B) macchina a vapore
C) acqua
D) vento
E) motore a scoppio

11. Delle seguenti locuzioni, quale esprime il significato del termine «astenia»?
A) Mancanza d'appetito
B) Sensibilità ridotta
C) Eccitazione generale
D) Scomposta iperattività
E) Mancanza di forze

12. Il logopedista si occupa di:
A) educazione fonetica
B) corsa compestre
C) chirurgia del piede
D) cura estetica degli arti inferiori
E) computers

13. Qual è il significato di bulimia?
A) Mancanza di memoria
B) Disturbo del linguaggio
C) Appetito insaziabile
D) Perdita dell'appetito
E) Mancanza di ossigeno

14. Che cos'è la dialettica?
A) Una branca della medicina
B) L'arte del discutere e del persuadere
C) La parlantina
D) Lo studio dei dialetti
E) La capacità d'ingannare

15. .... non si propone di guidare la coscienza degli individui verso il bene morale. Indica piuttosto i comportamenti che è opportuno tenere nell'ambito della professione o quelli da evitare per impedire che l'immagine sociale della professione stessa venga offuscata. Quale parola funge da soggetto a questo periodo?
A) La normativa
B) La correttezza
C) La statistica
D) L'etica
E) La deontologia

16. Quattro delle seguenti parole hanno una radice etimologica comune. Individuate quella anomala:
A) antibiotico
B) antifonario
C) antiquario
D) antipatia
E) antiemetico

17. Tema centrale dei *Malavoglia* di Verga è:
A) una storia di contrabbando di generi alimentari
B) l'iniquità dell'imposizione dell'IVA sul commercio dei lupini
C) il rischio di naufragi sulla costa siciliana
D) la difficile condizione di vita dei pescatori siciliani
E) una delle prime storie di mafia

18. *"Il sonno della ragione genera mostri".* Chi è l'autore di questa frase diventata famosa?
A) Francisco Goya
B) Galileo Galilei
C) Voltaire
D) Albert Einstein
E) Immanuel Kant

19. Il XX settembre è la ricorrenza di:
A) la proclamazione di Roma capitale
B) la breccia di Porta Pia
C) la spedizione dei Mille
D) il trattato di Campoformio
E) la battaglia di Solferino

20. Durante la Resistenza italiana, da chi fu assunta la direzione politica della guerra partigiana?
A) Dal Corpo Volontari della Libertà
B) Dal Comando alleato
C) Dal Comitato di Liberazione Nazionale
D) Dal Partito d'Azione
E) Dal Partito comunista

21. Paola non ha sorelle. Chi è la sorella del figlio del nonno materno della figlia di Paola?

A) Una cugina di Paola
B) Non esiste tale persona
C) Una zia di Paola
D) Paola stessa
E) La mamma di Paola

22. Ho 40 conigli bianchi e 31 conigli neri suddivisi in 10 gabbie.
Quale delle seguenti affermazioni è sicuramente vera?

A) In tutte le gabbie, il numero dei conigli bianchi è maggiore o uguale a quello dei conigli neri
B) In ogni gabbia ci sono almeno 7 conigli
C) Esiste almeno una gabbia in cui ci sono almeno 8 conigli
D) Esiste almeno una gabbia in cui ci sono esattamente 4 conigli neri
E) Esiste almeno una gabbia in cui ci sono almeno 5 conigli bianchi

23. L'italiano che afferma che tutti gli italiani sono sempre bugiardi dice:

A) niente di nuovo
B) una cosa ovvia
C) una bugia
D) il vero
E) una affermazione perfettamente verificabile

24. Quale delle seguenti affermazioni è FALSA?

A) Ci sono numeri pari che sono multipli di 12
B) Ci sono numeri pari che non sono multipli di 12
C) I multipli di 12 sono tutti pari
D) Tra i multipli di 12 ci sono tutti i numeri pari
E) Tra i numeri pari ci sono tutti i multipli di 12

25. Un uomo ha più figli, alcuni maschi e altre femmine. Ogni femmina ha un numero di fratelli doppio di quello delle sorelle ed ogni maschio ha un numero di sorelle pari a quello dei fratelli.
Quanti figli e quante figlie ha quest'uomo?

A) 6 femmine e 4 maschi
B) 4 femmine e 2 maschi
C) 2 maschi e 2 femmine
D) 3 maschi e 4 femmine
E) 3 femmine e 4 maschi

26. Se affermo "nessun elefante ha tre zampe", allora deduco che:

A) tutti gli elefanti hanno quattro zampe
B) tutti gli elefanti hanno tre zampe
C) almeno un elefante ha un numero di zampe diverso da tre
D) tutti gli elefanti hanno un numero di zampe diverso da tre
E) almeno un elefante ha tre zampe

27. Gli italiani sono sciatori. Gli sciatori possono essere tennisti.
Indicare con quale delle seguenti conclusioni può essere completato il sillogismo proposto:
- A) gli italiani possono essere tennisti
- B) alcuni italiani sono tennisti
- C) il sillogismo non può essere completato
- D) gli italiani sono tennisti
- E) alcuni tennisti sono italiani

28. Individuare il numero che segue logicamente: 100, 95, 85, 70, 50, ?
- A) 30
- B) 35
- C) 25
- D) 20
- E) 15

29. In un ristorante 15 avventori stanno seduti, 10 mangiano una torta. Con questi dati si può concludere con certezza che il numero totale N degli avventori è:
- A) $N > 25$
- B) $N \geq 15$
- C) $N < 15$
- D) $N > 15$
- E) $N = 25$

30. Tutti i filosofi sono antipatici – qualche filosofo è italiano – dunque .... è antipatico. S'individui il CORRETTO COMPLETAMENTO del sillogismo:
- A) ogni filosofo
- B) ogni italiano
- C) qualche antipatico
- D) qualche filosofo
- E) qualche italiano

31. Una banda musicale marcia formando un quadrato di 6 x 6 elementi. Arrivata in piazza, si dispone su 3 file, formando un rettangolo, per suonare. Di quanti elementi è composta ciascuna fila?
- A) 12
- B) 24
- C) 16
- D) 9
- E) 6

32. Tre campane suonano ad intervalli di 4 minuti, 12 minuti e 9 minuti, rispettivamente. Se suonano insieme alle 8 di mattina, dopo quanto tempo suoneranno di nuovo insieme?
- A) 72 minuti
- B) 29 minuti
- C) 24 minuti
- D) 36 minuti
- E) 16 minuti

33. Una gallina ha fatto i pulcini. Maria dice: *"Sono nati tre pulcini e sono gialli"*. Gianni dice: "Sono nati due pulcini gialli e due neri". Anna dice: "sono nati solo pulcini gialli". Quanti pulcini sono nati, sapendo che una e una sola delle precedenti affermazioni è falsa?

A) Non è possibile stabilire il numero con certezza
B) 4
C) 3
D) 6
E) 2

34. Se su un biliardo ci sono due biglie davanti a due biglie e due biglie dietro a due biglie, qual è il numero minimo di biglie presenti?

A) 5
B) 8
C) 6
D) 2
E) 4

35. Completare la seguente serie numerica: 11, 7, 22, 21, 44, ?, ?, 189, 176

A) 28; 55
B) 63; 66
C) 35; 77
D) 63; 88
E) 42; 88

36. Se A viene prima di C, E viene prima di C, C viene prima di D ed A viene prima di E, una delle seguenti affermazioni è falsa (F) mentre tutte le altre sono vere (V):

1. A è la prima della serie
2. E viene dopo D
3. E non è l'ultima della serie
4. E viene prima di D
5. L'ordine non è alfabetico

Quale tra le seguenti è dunque la sequenza corretta?

A) 1:F 2:V 3:V 4:V 5:V
B) 1:V 2:V 3:V 4:F 5:V
C) 1:V 2:V 3:V 4:V 5:F
D) 1:V 2:V 3:F 4:V 5:V
E) 1:V 2:F 3:V 4:V 5:V

37. Completare la seguente proporzione: Tranquillo : X = Y : Discreto

A) X = Lago, Y = Perbene
B) X = Tradito, Y = Descritto
C) X = Agitato, Y = Passabile
D) X = Irrequieto, Y = Invadente
E) X = Possibile, Y = Buono

38. Scartare l'intruso.

A) 17
B) 29
C) 31
D) 11
E) 27

39. In una comunità di 5000 persone il 5% dei membri viene colpito da una malattia infettiva, che richiede il ricovero nel 50% dei casi; quanti ricoveri sono avvenuti?
A) 125
B) 200
C) 150
D) 100
E) 50

40. Individuare il numero che completa correttamente la seguente successione: 7 – 14 – 42 – 168 – ?
A) 182
B) 840
C) 672
D) 310
E) 210

Test di Biologia

41. La colchicina inibisce la formazione del fuso mitotico. Nelle cellule eucariotiche trattate con questa sostanza:
A) viene persa la forma
B) non avviene la divisione cellulare
C) non avviene il crossing-over
D) non si condensano i cromosomi
E) non si duplica il DNA

42. La rana (*Rana esculenta*) e il rospo (*Bufo bufo*) appartengono:
A) a due classi diverse
B) a due ordini diversi
C) allo stesso genere, ma a due specie diverse
D) alla stessa specie, ma a generi diversi
E) allo stesso ordine, ma a genere e specie diversi

43. Durante lo sviluppo embrionale, come si chiama la struttura a 3 strati di cellule?
A) Blastula
B) Gastrula
C) Blastocele
D) Zigote
E) Archenteron

44. Il succo enterico:
A) è prodotto dal pancreas e versato nella cistifellea
B) è prodotto dal pancreas e versato nell’intestino
C) è prodotto dalla mucosa intestinale e versato nel duodeno
D) è prodotto dall’intestino e versato nello stomaco
E) è prodotto dallo stomaco

45. La rottura della membrana plasmatica e il rigonfiamento del nucleo si ottengono trattando le cellule con una soluzione:
- A) ipotonica
- B) di NaCl al 9 per mille
- C) isosmotica
- D) isotonica
- E) ipertonica

46. Qual'è il nome della cellula nervosa?
- A) Neurite
- B) Piastrina
- C) Neurone
- D) Dendrite
- E) Sarcomero

47. I mitocondri sono presenti nei batteri?
- A) Si, ma solo in particolari condizioni ambientali
- B) Si, ma solo nei batteri facoltativi
- C) Si, ma solo nei batteri a respirazione anaerobica
- D) No, mai
- E) Si, ma solo nei batteri a respirazione aerobica

48. Da quale ormone è stimolata l'ovulazione?
- A) Cortisolo
- B) ACTH
- C) TSH
- D) Ossitocina
- E) FSH

49. Il sito della sintesi proteica è il:
- A) lisosoma
- B) mitocondrio
- C) nucleo
- D) nucleolo
- E) reticolo endoplasmatico rugoso

50. La dicitura *Macaca silenus*, con la quale viene indicata la macaca dalla coda di leone, si riferisce alle seguenti categorie tassonomiche:
- A) genere, specie
- B) famiglia, specie
- C) ordine, specie
- D) classe, specie
- E) specie, sottospecie

51. L'articolazione fra le vertebre è detta:
- A) mobile
- B) semimobile
- C) immobile
- D) a leva
- E) fissa

52. I lieviti del genere *Saccharomices* sono:
A) vegetali
B) batteri
C) alghe
D) funghi
E) procarioti

53. Gli acari e le zecche appartengono:
A) al gruppo dei cefalopodi
B) alla classe degli aracnidi
C) alla classe dei nematodi
D) alla classe dei crostacei
E) alla classe degli insetti

54. I virus possono moltiplicarsi solo all'interno di una cellula ospite perchè:
A) sono troppo piccoli
B) sono molto sensibili ai raggi UV
C) non possiedono proteine
D) non possiedono DNA o RNA
E) sono privi di capacità biosintetica

55. Il numero delle molecole di adenina in una molecola di DNA è uguale al numero di molecole di:
A) fosforo
B) uracile
C) timina
D) guanina
E) citosina

56. L'allele è:
A) un gene disattivato
B) la parte non funzionale di una sequenza nucleotidica
C) un gene recessivo in omozigosi
D) un gene dominante in omozigosi
E) una delle due o più forme alternative di un gene

57. La circolazione doppia e completa compare per la prima volta:
A) negli uccelli
B) negli anfibi
C) in tutti i vertebrati
D) nei pesci ossei
E) nei ciclostomi

58. I protozoi:
A) sono organismi eucarioti unicellulari
B) si riproducono solo sessuatamente
C) presentano dimensioni intermedie tra quelle dei batteri e dei virus
D) sono incapaci di qualsiasi locomozione
E) sono sinonimi di procarioti

Test di Chimica

59. Il colesterolo è:
A) una vitamina
B) uno steroide
C) una tossina animale
D) una proteina vegetale
E) uno zucchero

60. Quali legami chimici determinano la struttura primaria di una proteina?
A) Legami dativi
B) Forze di Van der Waals
C) Interazioni idrofobiche
D) Legami idrogeno
E) Legami covalenti

61. Il legame covalente è dovuto alla compartecipazione di:
A) cariche positive e negative
B) un solo protone fra due atomi
C) elettroni e protoni fra due atomi
D) almeno due elettroni fra due atomi
E) almeno due protoni fra due atomi

62. Se 1 Kg di etilbenzene fonde a – 95 °C, 500 g della stessa sostanza:
A) fondono a 89 K
B) fondono a + 47,5 °C
C) fondono a + 95 °C
D) fondono a – 95 °C
E) fondono a – 47,5 °C

63. La distillazione è un metodo che separa due o più liquidi che si basa su:
A) la differente massa
B) la differente energia cinetica
C) il differente punto di ebollizione
D) il differente peso specifico
E) la differente densità

64. Quale delle seguenti soluzioni conduce in maniera apprezzabile la corrente elettrica?
A) Soluzione di metano in benzina
B) Soluzione acquosa di bromuro di sodio
C) Soluzione di cloruro di sodio in metanolo puro
D) Soluzione acquosa di alcool etilico
E) Soluzione acquosa di glucosio

65. Quanti elettroni possono essere contenuti al massimo in un orbitale?
A) 2
B) Dipende dal tipo dell'orbitale
C) 32
D) 18
E) 8

66. Quale delle seguenti sostanze si scioglie meglio in acqua?
A) La cellulosa
B) Un grasso neutro
C) La benzina
D) Il solfato di potassio
E) L'etere dietilico

67. Quale membrana viene usata per mettere in evidenza la pressione osmotica?
A) Nessuna delle altre risposte è corretta
B) Non si usano membrane speciali
C) Membrana semipermeabile
D) Membrana impermeabile
E) Membrana permeabile

68. Gli idruri sono composti binari:
A) nei quali l'idrogeno è legato a un altro elemento
B) di un metallo alcalino con idrogeno
C) di un metallo con l'ossigeno
D) di un non-metallo con l'ossigeno
E) di un alogeno con l'idrogeno

69. La struttura delle membrane cellulari è caratterizzata dalla presenza di:
A) esteri del colesterolo
B) triacilgliceroli
C) acidi grassi semplici
D) qualunque tipo di lipidi
E) fosfolipidi

Test di Fisica e Matematica

70. Quale delle seguenti disuguaglianze è VERA?
A) $10^{-100} < 100^{-10}$
B) $100^{-10} < 10^{-100}$
C) $10^{100} < -100^{10}$
D) $-10^{100} < -100^{10}$
E) $10^{100} < 100^{10}$

71. Quanti sono i divisori (con resto nullo) del numero 100, 1 e 100 compresi?
A) 7
B) 6
C) 4
D) 9
E) 2

72. Se il logaritmo in base 9 di *x* è uguale a −3 allora:
A) $x = 729$
B) l'equazione non ha senso perchè il valore di un logaritmo non può mai essere negativo
C) $x = 1/729$
D) $x = 1/3$
E) l'equazione non ha senso perchè la base è maggiore di 1

73. Due sfere hanno raggi di lunghezza l'una tripla dell'altra. Qual è il rapporto tra la misura del volume della sfera di raggio maggiore e quella del volume della sfera di raggio minore?

A) 2π

B) 3π

C) 27

D) 9

E) π

74. Data la sequenza di numeri 1, 2, 5, 4, 9, 6, 13, ... qual è il successivo termine?

A) Non può essere predetto perchè la sequenza è puramente casuale

B) 7

C) 10

D) 11

E) 8

75. Quanti sono i modi distinti di realizzare un poker d'assi (4 assi e 1 carta diversa) scegliendo in un mazzo di 52 carte da gioco? (L'ordine di scelta delle carte non ha importanza)

A) 52

B) 48

C) 26

D) 4

E) 13

76. Un corpo ha una certa massa M. Se viene portato sulla Luna, la sua massa:

A) dipende dalla densità dell'atmosfera lunare

B) si annulla

C) aumenta

D) diminuisce

E) non varia

77. Un vetro per occhiali protettivi lascia passare 1/5 della luce incidente. Quale frazione della luce incidente passerà attraverso tre strati sovrapposti dello stesso vetro?

A) $(4/5)^3$

B) 1/15

C) $(1/5)^3$

D) 3/5

E) nessuna

78. Dimensionalmente l'energia è:

A) il rapporto tra una potenza e un intervallo di tempo

B) il quadrato di una velocità

C) il rapporto tra una forza e uno spostamento

D) il prodotto di una forza per una velocità

E) il prodotto di una forza per uno spostamento

79. Una pattinatrice su ghiaccio sta piroettando con le braccia strette al corpo. A un certo punto allarga improvvisamente le braccia. Indicare l'affermazione più probabile tra le seguenti:

A) la velocità di rotazione dipende dall'affilatura dei pattini
B) la velocità di rotazione diminuisce
C) la velocità di rotazione dipende dallo stato del ghiaccio
D) la velocità di rotazione rimane inalterata
E) la velocità di rotazione aumenta

80. Se in un circuito chiuso costituito da una batteria, una resistenza e un conduttore, la corrente è uguale a 2 A e la resistenza totale è uguale a 10 ohm, la potenza dissipata dalla resistenza è:

A) 4 W
B) 40 W
C) 20 W
D) 200 W
E) 5 W

********** FINE DELLE DOMANDE **********

PROVA DI AMMISSIONE AI CORSI DI LAUREA DELLE PROFESSIONI SANITARIE

Anno Accademico 2011/2012

Test di Ragionamento logico

1. Una delle seguenti NON è un'istituzione europea.
A) Parlamento europeo
B) Commissione europea
C) Tribunale amministrativo europeo
D) Banca Centrale Europea
E) Consiglio europeo

2. Sir A. Fleming vinse il premio Nobel per aver scoperto:
A) un ormone surrenalico
B) la penicillina
C) un liquido di fissaggio
D) le piastrine
E) il lisozoma

3. Qual'è l'argomento del libro di Roberto Saviano *"Gomorra"*?
A) fatti fantastici e immaginari
B) malviventi e banditi italiani
C) autobiografia dell'autore
D) nomi e fatti della camorra
E) viaggio nell'impero economico napoletano

**4. "*Perciò io penso che il .... non nasca dal rimpianto di una mala azione già commessa ma dalla visione della propria colpevole disposizione. La parte superiore del corpo si china a guardare e giudicare l'altra parte e la trova deforme. Ne sente ribrezzo e questo si chiama ....*
Anche nella tragedia antica la vittima non ritornava in vita e tuttavia il .... passava. Ciò significava che la deformità era guarita e che ormai il pianto altrui non aveva alcuna importanza. Dove poteva esserci posto per il .... in me che con tanta gioia e tanto affetto correvo dalla mia legittima moglie? Da molto tempo non m'ero sentito tanto puro".**

(I. Svevo, "*La coscienza di Zeno*")

In questo testo UNO stesso termine ricorre per quattro volte: qual è?
A) rimorso
B) bene
C) dolore
D) pensiero
E) coraggio

5. **Dal brano seguente abbiamo espunto qualcosa.**
"*Quando .... si muove in modo uguale, in apparenza non si muove niente, come su una nave. Quando tutti vanno verso la dissolutezza, sembra che nessuno ci vada. Colui che si ferma mette in evidenza l'esagerazione degli altri, come se fosse un punto fisso.*"
(Tratto dal pensiero 592 di B. Pascal, tr. it. *La spiga*, Milano, 2002)
Lo si reintegri con UNA delle alternative sottostanti logicamente coerenti con il senso del brano.
A) uno
B) lo sfondo
C) qualcosa
D) qualcuno
E) tutto

6. **Dal brano seguente abbiamo espunto qualcosa.**
"*La curiosità spesso non è altro che vanità; si vuol conoscere solo per parlarne; non si viaggerebbe sul mare per non .... mai e per il solo piacere di vedere.*"
(Tratto dal pensiero 72 di B. Pascal, tr. it. *La spiga*, Milano, 2002)
Lo si reintegri con UNA delle alternative sottostanti logicamente coerenti con il senso del brano.
A) immaginarlo
B) parlarne
C) attraversarlo
D) rivederlo
E) tornarvi

7. **"*Il maestro disse: «Soltanto dopo i primi freddi ci accorgiamo che il pino e il cipresso conservano più a lungo le loro foglie*»."**
(da Confucio, *Massime*, a c. di P. Santangelo, Roma, Newton Compton, 2005, p. 92)
Fuor di metafora l'aforisma intende verosimilmente suggerire che:
A) gli uomini sono come le foglie degli alberi d'autunno
B) i primi freddi sfrondano la maggior parte delle piante
C) solo gli alberi d'alto fusto resistono alle intemperie
D) non tutte le specie vegetali possiedono la stessa vitalità
E) nei momenti difficili si riconoscono gli uomini superiori

8. **Tutti gli studenti amano i libri.**
Chi partecipa ad assemblee è sovente un rivoluzionario.
Alcuni rivoluzionari sono studenti.
Se le precedenti affermazioni sono vere, quale delle seguenti deduzioni è infondata?
A) alcuni studenti è probabile che partecipino ad assemblee
B) è possibile che alcuni rivoluzionari amino i libri
C) chi partecipa alle assemblee può amare i libri
D) non si può dire che chi studia non ama i libri
E) chi ama i libri è un rivoluzionario

9. **Il "welfare state" indica:**
A) il superamento delle discriminazioni razziali
B) Lo stato sociale che assicura il benessere dei cittadini
C) la tutela dei diritti del bambino
D) la parità dei diritti uomo-donna
E) il raggiungimento del benessere psicofisico

10. Lo “statuto dei lavoratori” è:
A) un documento unitario dei sindacati confederali per difendere i diritti dei lavoratori
B) un protocollo di intesa per la stesura dei contratti
C) una legge dello Stato italiano
D) un accordo privato tra azienda e lavoratori
E) un contratto sindacale

11. Quando si parla di “esecutivo” si fa riferimento:
A) ai prefetti
B) al Governo
C) all’insieme di Regioni, Provincie, Comuni
D) al Parlamento
E) al Presidente della Repubblica

12. Dirimere una questione vuole dire:
A) esasperarla
B) rinviarla
C) esprimerla
D) risolverla
E) travisarla

13. Una persona con molti interessi diversi si può definire un tipo:
A) eclettico
B) sinottico
C) didattico
D) dialettico
E) ellittico

14. Una delle frasi famose qui riportate NON è assegnata all’autore a cui è comunemente attribuita:
A) *“Libera Chiesa in libero Stato”* (Camillo Benso, Conte di Cavour)
B) *“Venni, vidi, vinsi”* (Giulio Cesare)
C) *“Volli, sempre volli, fortissimamente volli”* (Vittorio Alfieri)
D) *“Parigi val bene una messa”* (Napoleone Bonaparte)
E) *“Guai ai vinti”* (Brenno)

15. La spiegazione di uno dei seguenti modi di dire NON è CORRETTA:
A) è lapalissiano: è talmente evidente da essere scontato
B) sbarcare il lunario: vivere al di sopra delle proprie possibilità
C) mettere all’indice: vietare qualcosa ritenuto riprovevole
D) questioni di lana caprina: questioni senza importanza
E) andare per la maggiore: riscuotere un notevole successo

16. Indicare l’unica parola specifica dell’ambito biomedico:
A) antinflativo
B) antimicotico
C) antitetico
D) antinomico
E) antifrastico

17. Quale dei seguenti aggettivi, ricavati dal nome di celebri scrittori e filosofi, viene usato comunemente nel significato di “paradossale”?

A) cartesiano
B) dantesco
C) kantiano
D) boccaccesco
E) kafkiano

18. Se devo scrivere un testo descrittivo in prosa, per stabilire il posto che le parole occupano nella frase, come si compongono le frasi, come le frasi si dispongono a formare un periodo, faccio riferimento alle regole di:

A) semiotica
B) lessico
C) sintassi
D) metrica
E) prosodia

19. Uno stile caustico è proprio di uno scrittore:

A) letterario e tradizionalista
B) oscuro e pretenzioso
C) graffiante ed ironico
D) involuto e prolisso
E) scurrile e sboccato

20. Indicare la coppia in cui NON si verifica il tipo di relazione presente nelle altre quattro:

A) violino – regista
B) capitolo – romanzo
C) finestra – palazzo
D) ali – fringuello
E) tasca – cappotto

21. Qual’è l’unica parola che significa “rottura”?

A) effrazione
B) elucubrazione
C) estrazione
D) rifrazione
E) diffrazione

22. Una di queste frasi NON è una metafora:

A) quel bambino è un terremoto
B) Tizio è un’aquila
C) inciampare in una difficoltà
D) sei bello come il sole
E) quest’aula è una bolgia

23. Rosso Malpelo è un personaggio creato da:

A) Dacia Maraini
B) Andrea Cammilleri
C) Ignazio Silone
D) Mario Tobino
E) Giovanni Verga

24. Quali dei seguenti verbi si avvicina di più al significato di "asserire"?
A) annuire
B) affermare
C) conferire
D) assegnare
E) dimostrare

25. Una di queste parole NON si spiega etimologicamente con il prefisso "meta":
A) metastasi
B) metamorfosi
C) metafora
D) metallo
E) metabolismo

26. "Pagare a pronta cassa" significa:
A) pagare dopo sollecito di pagamento
B) pagare soltanto alla posta
C) pagare entro i termini indicati sul contratto
D) pagare in banca
E) pagare in contanti

27. Luigi Pasteur fu un:
A) allievo di Freud
B) regista cinematografico
C) batteriologo
D) astronauta
E) letterato francese

28. La semeiotica o semiologia è la disciplina che si occupa (segnare la definizione CORRETTA):
A) dell'etimologia delle parole
B) del modo in cui i segni vengono prodotti, trasmessi, interpretati, accettati e rifiutati
C) del rapporto tra le espressioni linguistiche e gli oggetti a cui si riferiscono
D) degli elementi di somiglianza tra discorsi scientifici diversi
E) dell'individuazione delle specie di semi

29. Qual'è il significato del termine "dicastero"?
A) è l'ufficio amministrativo della Presidenza del Consiglio che coordina l'attività di tutti i ministeri
B) si chiamano così solo i ministeri senza portafoglio
C) si usa per indicare il governo composto solo da dieci ministeri
D) è il termine usato per indicare solo i ministeri "chiave" di un governo
E) è sinonimo del termine "ministero"

30. Individuate il termine anomalo:
A) mielato
B) mellifluo
C) mieloma
D) mellifero
E) mieloso

31. Se un pubblico ufficiale per eseguire un atto del suo ufficio riceve per sé o per un terzo, in denaro o altra utilità, una retribuzione che non gli è dovuta, o ne accetta la promessa compie un reato di:

A) corruzione
B) abuso d'ufficio
C) falsità ideologica
D) concussione
E) peculato

32. Quale suffisso delle parole sotto elencate è etimologicamente diverso?

A) equinoziale
B) equivalente
C) equino
D) equilatero
E) equo

33. Cinque persone (A, B, C, D, E) decidono di scambiarsi i regali di Natale di modo che ciascuno faccia un regalo a due persone e ne riceva da altre due persone. A fa un regalo a B e C; D fa un regalo a B e a una delle due persone che ricevono il regalo anche da E; C fa un regalo a D e alla stessa persona che riceve il regalo anche da B.
Da chi riceve i regali E?

A) C e A
B) B e C
C) D e A
D) B e D
E) D e C

34. È stata respinta la prova che nega che A è uguale a B, ma non è stata acquisita alcuna prova del contrario. Quindi:

A) A è diverso da B al 100%
B) A potrebbe essere uguale a B
C) A è uguale a B al 99%
D) A è sicuramente diverso da B
E) A è sicuramente uguale a B

35. Se scuola : X = ospedale : Y, che cosa sono rispettivamente X e Y?

A) X = medico Y = infermiere
B) X = bidello Y = studente
C) X = insegnante Y = malato
D) X = insegnante Y = medico
E) X = studente Y = insegnante

36. Inserisci i numeri mancanti:

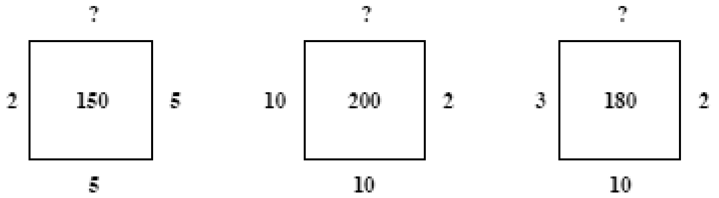

A) 7 – 14 – 14
B) 6 – 2 – 3
C) 5 – 10 – 5
D) 8 – 4 – 2
E) 3 – 1 – 3

37. Quale numero è da eliminare dalla seguente successione:
1, 5, 8, 12, 15, 19, 20, 22, 26, 29, 33

A) 29
B) 20
C) 26
D) 19
E) 21

38. Considerando l'alfabeto italiano (21 lettere), qual è la probabilità di estrarre una vocale?

A) 3/21
B) 6/21
C) 5/21
D) 4/21
E) 1/21

39. Sei amici si apprestano a giocare una partita di pallavolo tre contro tre. In quanti modi posssono distribuirsi per formare le squadre?

A) 24
B) 36
C) 10
D) 27
E) 20

40. Completare la seguente serie:

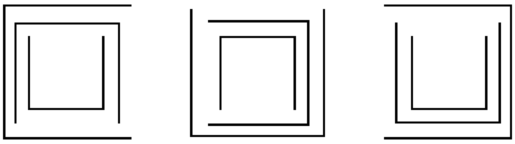

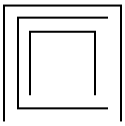

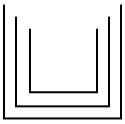

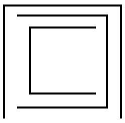

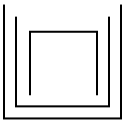

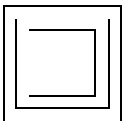

Test di Biologia

41. Nei mammiferi l'assorbimento dei principali nutrienti avviene:

A) nel retto
B) nel tenue
C) nel cieco
D) nello stomaco
E) nel colon

42. La struttura dell'occhio utile a variare la quantità di luce che entra è:

A) la sclerotica
B) la cornea
C) la retina
D) il cristallino
E) l'iride

43. Quante sono le vertebre toraciche?

A) 32
B) 24
C) 12
D) 7
E) 5

44. Gli enzimi possono accelerare una reazione:
- A) fornendo energia
- B) rendendo più stabili le molecole del substrato
- C) abbassando l'energia di attivazione
- D) rendendo spontanea una reazione endoergonica
- E) modificando la variazione di energia libera della reazione

45. I geni mitocondriali nella nostra specie:
- A) si trasmettono con il cromosoma X
- B) si trasmettono con il cromosoma Y
- C) si trasmettono per via materna
- D) non vengono trasmessi
- E) si trasmettono per via paterna

46. Molecole di ossigeno entrano in un globulo rosso mediante il processo di:
- A) pinocitosi
- B) diffusione
- C) trasporto attivo
- D) trasporto facilitato
- E) osmosi

47. Una differenza tra meiosi e mitosi è che:
- A) la meiosi si verifica solo negli organismi aploidi, la mitosi in tutti gli organismi
- B) il DNA si duplica prima della mitosi ma non prima della meiosi
- C) il DNA è maggiormente replicato in meiosi piuttosto che in mitosi
- D) durante la meiosi il DNA non è replicato
- E) i cromosomi omologhi si appaiano nella meiosi ma non nella mitosi

48. La cellula ricava energia attraverso:
- A) sintesi degli zuccheri
- B) rottura dei legami chimici
- C) sintesi dei lipidi
- D) elettrolisi
- E) aumento della temperatura

49. Se uno spermatozoo umano con cromosoma sessuale X feconda una cellula uovo a corredo cromosomico ignoto, il nascituro sarà:
- A) impossibile da prevedere
- B) probabilmente un maschio
- C) femmina
- D) al 50% di probabilità femmina e al 50% maschio
- E) maschio

50. Le cellule procariotiche mancano di:
- A) nucleo
- B) ribosomi
- C) parete
- D) membrana plasmatica
- E) DNA

51. Nella specie umana, per caratteri sessuali primari si intendono:
A) gli ormoni sessuali
B) la distribuzione dei peli
C) il timbro della voce
D) i genitali esterni
E) le gonadi

52. La bile ha la funzione di:
A) idrolizzare l'amido
B) facilitare l'emulsione dei grassi
C) idrolizzare i grassi
D) facilitare l'idrolisi di zuccheri complessi
E) facilitare l'emulsione delle proteine

53. La vaccinazione si basa sul fatto che:
A) contiene istamina
B) stimola la produzione di antigeni
C) contiene anticorpi
D) contiene antigeni
E) contiene sostanze che uccidono i microganismi patogeni

54. Individuare nel seguente insieme di codoni genetici quello errato:
A) CCC
B) AGG
C) UTT
D) GCC
E) UAA

55. Le specie appartenenti alla stessa classe sono incluse:
A) nello stesso phylum
B) nella stessa famiglia
C) nello stesso ordine
D) nella stessa specie
E) nello stesso genere

56. Tra le lamelle ossee del tessuto spugnoso delle ossa piatte è presente:
A) tessuto muscolare
B) midollo rosso
C) tessuto elastico
D) midollo spinale
E) midollo giallo

57. Le ghiandole sebacee e sudoripare sono tipicamente ghiandole:
A) surrenali
B) vestibolari
C) vescicolari
D) endocrine
E) esocrine

58. Quando la temperatura dell'ambiente esterno scende di molto al di sotto dello 0 °C, nella pelle scatta un meccanismo di termoregolazione che consiste in:

A) termodispersione
B) vasocostrizione delle arterie profonde del derma
C) aumento della sudorazione
D) vasodilatazione delle arteriole superficiali della pelle
E) vasocostrizione delle arteriole superficiali della pelle

Test di Chimica

59. Qual'è il corretto bilanciamento per Cl_2 + KOH ⟶ KCl + KClO + H_2O ?

A) Cl_2 + KOH ⟶ 2KCl + 4KClO + H_2O
B) Cl_2 + 2KOH ⟶ KCl + KClO + H_2O
C) $3Cl_2$ + 2KOH ⟶ KCl + 2KClO + $2H_2O$
D) Cl_2 + 3KOH ⟶ 2KCl + KClO + $2H_2O$
E) $2Cl_2$ + KOH ⟶ KCl + 3KClO + H_2O

60. Il peso atomico del cloro è 35,453 e non esattamente 35 perché:

A) ogni atomo di cloro contiene 18 neutroni
B) ogni atomo di cloro ha massa uguale a 35,453
C) ci sono almeno due isotopi naturali del cloro
D) tutti gli atomi di cloro hanno proprietà identiche
E) ogni atomo di cloro contiene 17 protoni

61. Quale di queste sostanze è un monosaccaride?

A) Maltosio
B) Amilosio
C) Saccarosio
D) Galattosio
E) Lattosio

62. Indicare le corrette associazioni:

A) Cl = cloro Fe = ferro H = elio
B) B = bromo C = carbonio Au = oro
C) F = ferro Be = berillio B = boro
D) Cu = rame Hg = mercurio K = potassio
E) S = sodio F = fluoro B = boro

63. I metalli sono:

A) tutti solidi tranne il vanadio
B) tutti solidi tranne l'alluminio
C) tutti liquidi tranne il mercurio
D) tutti solidi tranne il mercurio
E) tutti solidi tranne il sodio

64. Una reazione si definisce endotermica quando:

A) diminuisce di velocità
B) presenta una velocità di reazione elevata
C) avviene con assorbimento di calore
D) si trova all'equilibrio
E) avviene con sviluppo di calore

65. essendo R e R’ generici residui organici, quale dei seguenti composti è un’aldeide?
A) $R–CH_2OH$
B) $R–CH_2–O–CH_3$
C) R–COOH
D) R–COOR’
E) R–CHO

66. Nella tavola periodica degli elementi l’energia di ionizzazione dall’alto in basso lungo un gruppo:
A) diminuisce solo negli ultimi tre gruppi
B) decresce progressivamente
C) cresce nei primi tre gruppi, resta invariata negli altri
D) resta invariata
E) cresce progressivamente

67. Il peso molecolare del glucosio $C_6H_{12}O_6$ è 180 u.m.a. Quante molecole sono presenti in 18 g di glucosio?
A) $3,42 \cdot 10^{10}$
B) $6,02 \cdot 10^{23}$
C) $6,02 \cdot 10^{22}$
D) 3420
E) 342

68. La pila è un dispositivo normalmente utilizzato per:
A) trasformare energia chimica in energia elettrica
B) trasformare energia potenziale in energia chimica
C) utilizzare energia elettrica per effettuare una reazione chimica
D) trasformare energia termica in energia elettrica
E) trasformare energia chimica in calore

69. Che cos’è il numero di massa di un elemento?
A) il numero degli elettroni più quello dei protoni
B) la somma del numero di protoni e di neutroni
C) la quantità in grammi uguale al numero atomico
D) il rapporto tra la sua massa media ed il dalton
E) la sua massa in grammi

Test di Fisica e Matematica

70. Indica la somma dei primi 50 numeri dispari:
A) 2500
B) 6500
C) 4500
D) 3500
E) 500

71. Ad un convegno partecipano 21 persone. Ciascuno dei partecipanti stringe la mano a ciascuno degli altri. Quante sono state complessivamente le strette di mano?
A) Nessuna della altre risposte è corretta
B) 384
C) 420
D) 210
E) 42

72. Un dado viene lanciato 2 volte: qual è la probabilità che nel primo lancio esca un numero pari e nel secondo il numero 6?
A) 1/2
B) 1/12
C) 1/8
D) 2/3
E) 1/4

73. $sen^2\alpha + cos^2\alpha = 1$ vale per:
A) tutti i valori di α
B) alcuni valori di α
C) nessun valore di α
D) α positivi
E) α negativi

74. Quale delle seguenti equazioni rappresenta una curva passante per l'origine?
A) $x = 3$
B) $y = 2$
C) $y = x^2$
D) $y = x^2 – 1$
E) $y = 3x – 3$

75. Un'infermiera deve numerare 238 provette progressivamente (da 1 a 238) con etichette numerate da 0 a 9. Quante etichette dovrà utilizzare per portare a termine il lavoro affidatole?
A) Nessuna della altre risposte è corretta
B) 606
C) 605
D) 238
E) 3

76. In una gara di atletica al "via" l'atleta A parte con velocità 8 m/s mentre l'atleta B attende 2 secondi e poi parte con velocità 10 m/s. Quanti metri dovrà fare B per raggiungere A?
A) 60
B) 32
C) 16
D) 8
E) 80

77. Un quarzo compie 10^7 oscillazioni al secondo. Qual è il periodo di oscillazione?
- A) 10^{-7} s
- B) 10 s
- C) 10^7 Hz
- D) 10^{-7}Hz
- E) 10^7 s

78. Un condensatore da 8 μF richiede:
- A) Nessuna delle altre risposte è corretta
- B) 8 μC per caricarlo a 1 V
- C) 8 C per caricarlo a 1 V
- D) 1 μC per caricarlo a 8 V
- E) 8 C per caricarlo a 8 μV

79. Se il diametro di un conduttore viene dimezzato, la sua resistenza:
- A) rimane costante
- B) diventa la quarta parte
- C) dimezza
- D) raddoppia
- E) diventa quattro volte maggiore

80. In un circuito due resistenze, rispettivamente da 10 e 20 ohm, sono connesse in parallelo ad un generatore ideale da 12 V. La potenza totale sviluppata dal circuito è circa:
- A) 18 W
- B) 36 W
- C) 12 W
- D) 21 W
- E) 6 W

********** FINE DELLE DOMANDE **********
