# Supplementary material for: Comparative accuracy of ChatGPT-4, Microsoft Copilot and Google Gemini in the Italian entrance test for healthcare sciences degrees: a cross-sectional study
Source: BMC Med Educ. 2024 Jun 26;24:694. doi: 10.1186/s12909-024-05630-9 (PMC11210096; doi:10.1186/s12909-024-05630-9)
Supplement: Supplementary file 2 — Supplementary Material 2. [file 12909_2024_5630_MOESM2_ESM.pdf]

Additional File 2:

Categorisation of answer

Examples of categorization of correct answers

L

Un'auto di massa 1,5 t e velocità di 100 km/h in modulo urta un furgone di massa 4 t e velocità di 80 km/h in modulo, procedendo entrambi nella stessa direzione e verso. Se subito dopo l'urto i due mezzi procedono insieme, come un tutt'uno, determinare il modulo della velocità con cui il sistema si muove:

A) circa 85 km/h
B) circa 70 km/h
C) circa 42 km/h
D) circa 31 km/h
E) circa 10 km/h

Per risolvere questo problema, possiamo utilizzare il principio della conservazione della quantità di moto. La quantità di moto totale prima dell'urto è uguale alla quantità di moto totale dopo l'urto.

La quantità di moto p di un oggetto è definita come:

$$p = m \times v$$

dove m è la massa e v è la velocità.

Prima dell'urto:
Quantità di moto dell'auto p_1 = massa dell'auto m_1 x velocità dell'auto v_1
$p_1 = 1,5t \times 100km/h = 150t \times km/h$

Quantità di moto del furgone p_2 = massa del furgone m_2 x velocità del furgone v_2
$p_2 = 4t \times 80km/h = 320t \times km/h$

Quantità di moto totale prima dell'urto P_{tot_prima} = $p_1 + p_2$
$P_{tot\_prima} = 150t \times km/h + 320t \times km/h = 470t \times km/h$

Dopo l'urto, le due macchine si muovono come un tutt'uno con una massa combinata $m_{combinata}$ e una velocità $v_{combinata}$.

$$m_{combinata} = m_1 + m_2 = 1,5t + 4t = 5,5t$$

La quantità di moto totale dopo l'urto P_{tot_dopo} = $m_{combinata} \times v_{combinata}$

Poiché P_{tot_prima} = P_{tot_dopo} (principio della conservazione della quantità di moto),

$$470t \times km/h = 5,5t \times v_{combinata}$$

Dividendo entrambi i lati per 5,5 t, otteniamo:

$$v_{combinata} = \frac{470}{5,5} km/h = 85,45 km/h$$

Questo si arrotonda a:

$$v_{combinata} \approx 85 km/h$$

La risposta corretta è:

A) circa 85 km/h.

Rige

Figure 1a: Example of a correct answer labelled as "Logical reasoning", as the chatbot (ChatGPT-4) clearly demonstrate the logic presented in the response (question n. 51, CINECA test – 2023).

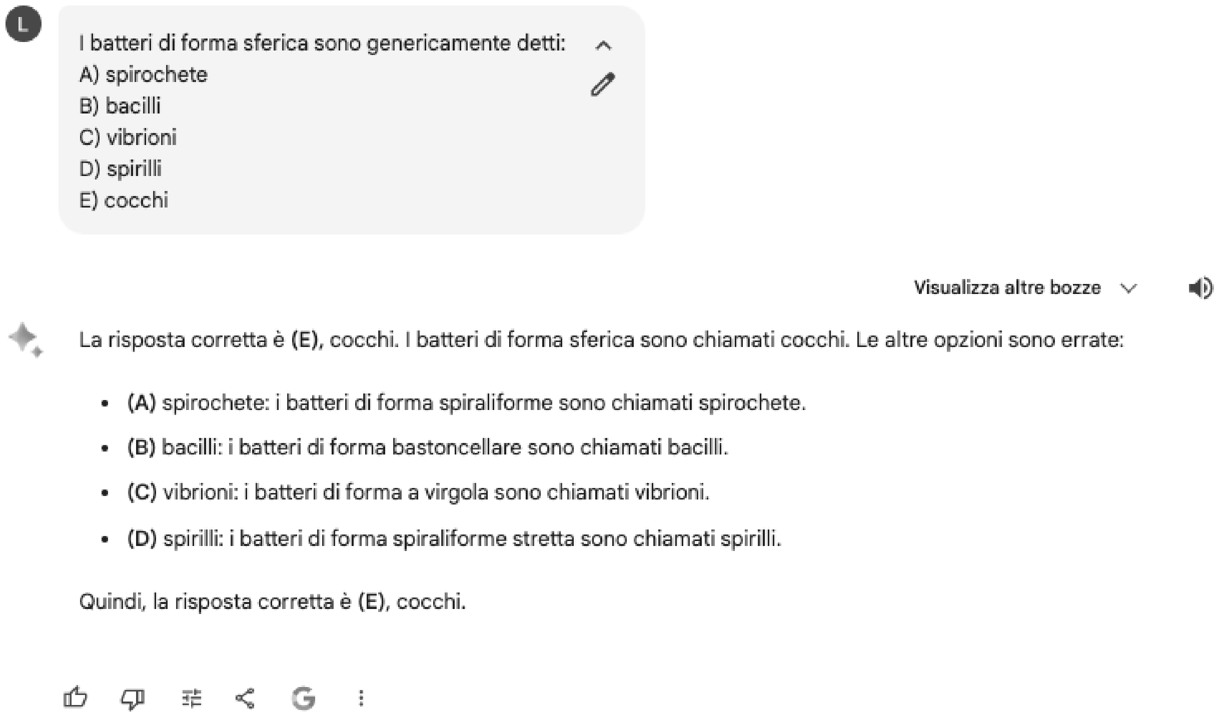

Figure 1b: Example of a correct answer labelled as "Internal information", as the chatbot (Bard) includes information internal to the question (question n. 29, CINECA test – 2019).

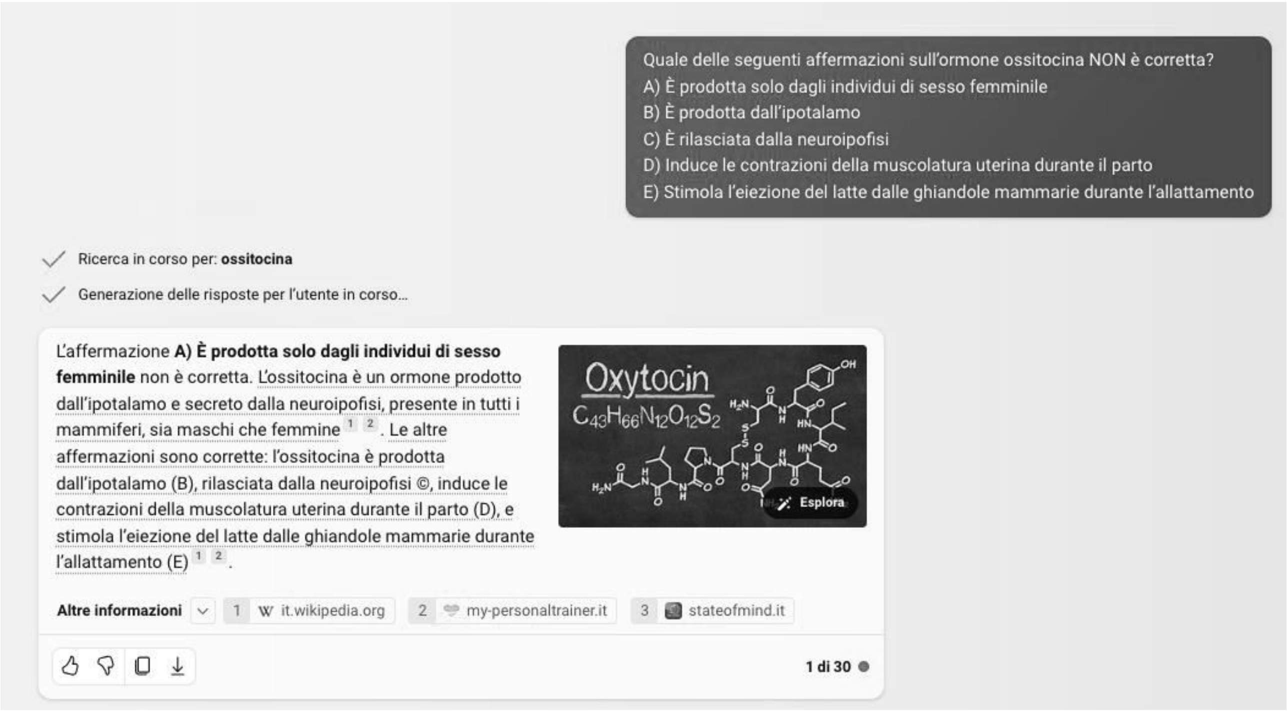

Figure 1c: Example of a correct answer labelled as "External information", as the chatbot (Microsoft Bing) use information external to the question to provide the answer, performing a web search and indicating the origin of the source from which the information is taken (question n. 30, CINECA test – 2023).

Examples of categorization of incorrect answers

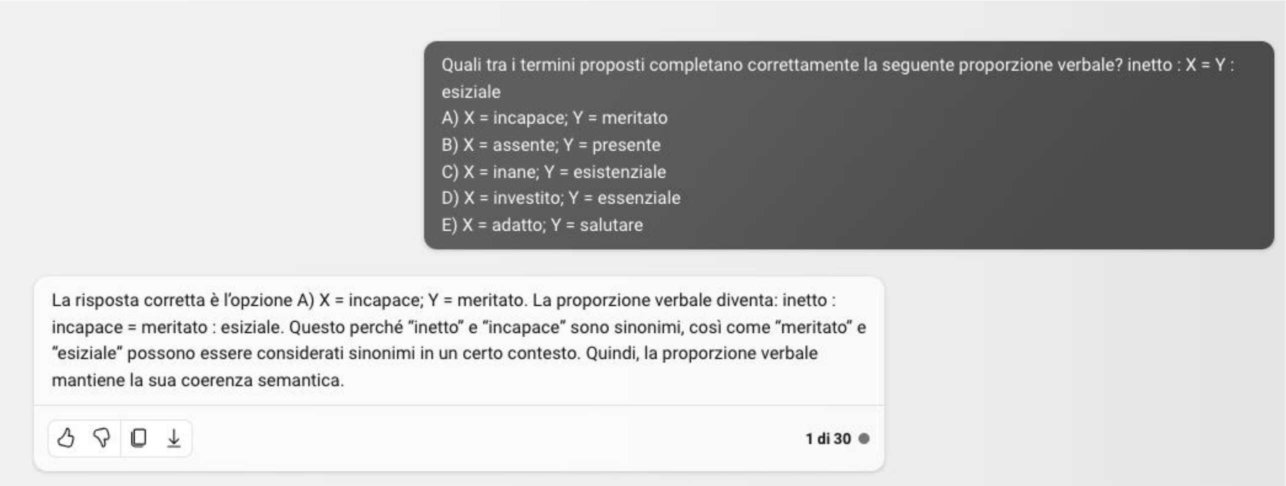

Figure 2a: Example of an incorrect answer labelled as "Logical error", as the chatbot (Microsoft Bing) correctly identifies the relevant information but fail to convert it into an appropriate answer (question n. 10, CINECA test – 2016; correct answer: E).

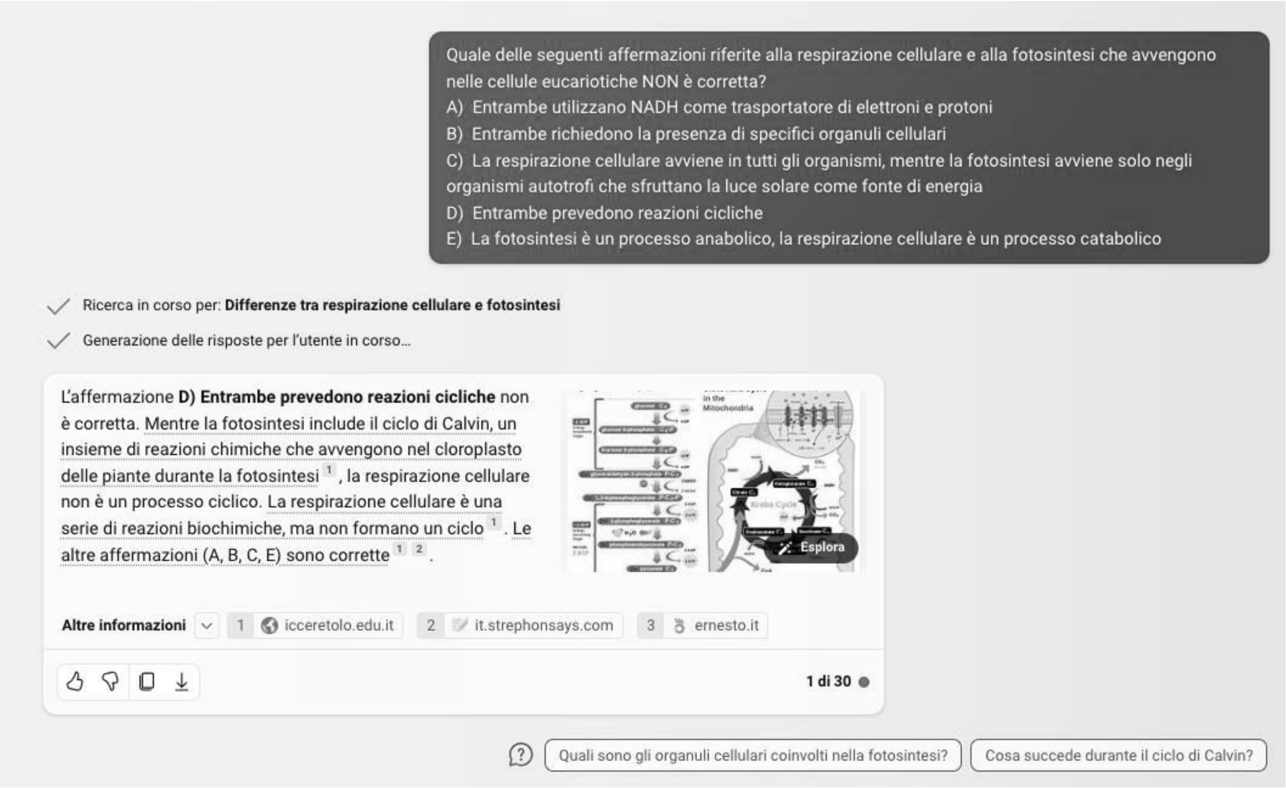

Figure 2b: Example of an incorrect answer labelled as "Information error", as the chatbot (Microsoft Bing) fails to recognize a key piece of information, whether present in the question stem or through external information (question n. 20, CINECA test – 2023; correct answer: A).

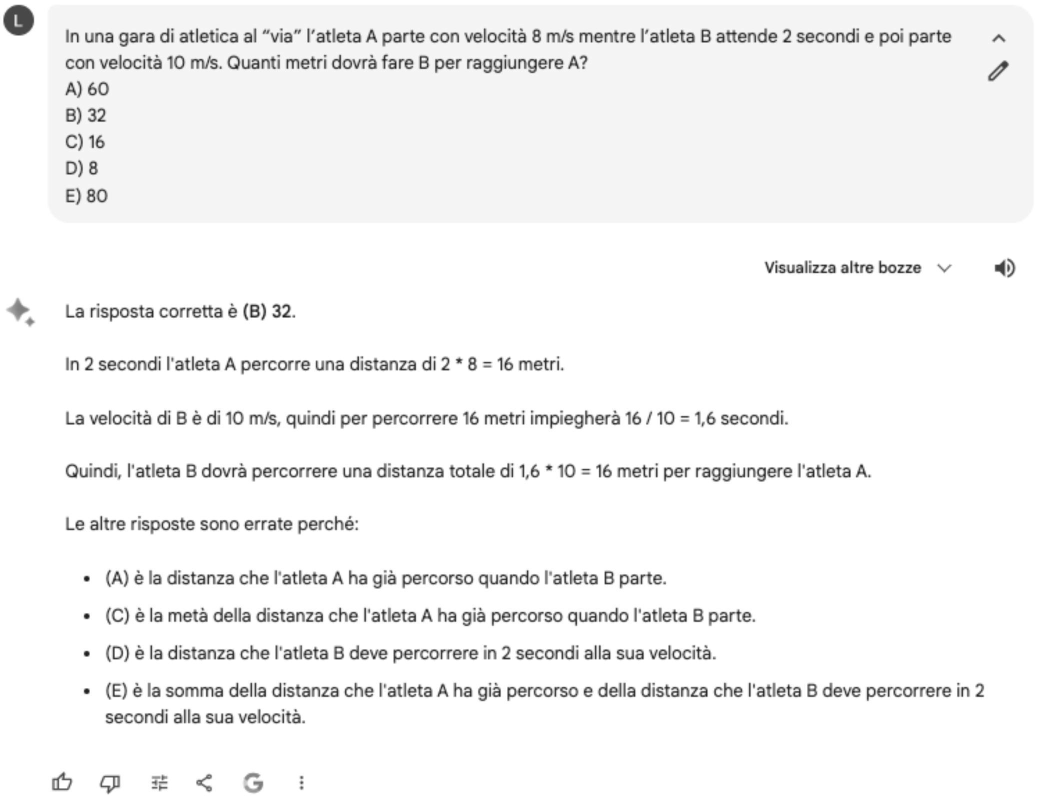

In una gara di atletica al “via” l’atleta A parte con velocità 8 m/s mentre l’atleta B attende 2 secondi e poi parte con velocità 10 m/s. Quanti metri dovrà fare B per raggiungere A?
A) 60
B) 32
C) 16
D) 8
E) 80

Visualizza altre bozze

La risposta corretta è **(B) 32**.

In 2 secondi l'atleta A percorre una distanza di 2 * 8 = 16 metri.

La velocità di B è di 10 m/s, quindi per percorrere 16 metri impiegherà 16 / 10 = 1,6 secondi.

Quindi, l'atleta B dovrà percorrere una distanza totale di 1,6 * 10 = 16 metri per raggiungere l'atleta A.

Le altre risposte sono errate perché:

- (A) è la distanza che l'atleta A ha già percorso quando l'atleta B parte.
- (C) è la metà della distanza che l'atleta A ha già percorso quando l'atleta B parte.
- (D) è la distanza che l'atleta B deve percorrere in 2 secondi alla sua velocità.
- (E) è la somma della distanza che l'atleta A ha già percorso e della distanza che l'atleta B deve percorrere in 2 secondi alla sua velocità.

Figure 2c: Example of an incorrect answer labelled as "Statistical error", as the chatbot (Bard) commits an error that involves an arithmetic mistake (question n. 76, CINECA test – 2011; correct answer: E).
