# Supplementary material for: Comparative accuracy of ChatGPT-4, Microsoft Copilot and Google Gemini in the Italian entrance test for healthcare sciences degrees: a cross-sectional study
Source: BMC Med Educ. 2024 Jun 26;24:694. doi: 10.1186/s12909-024-05630-9 (PMC11210096; doi:10.1186/s12909-024-05630-9)
Supplement: Supplementary file 3 — Supplementary Material 3. [file 12909_2024_5630_MOESM3_ESM.pdf]

Additional File 3:

Example of question (Figure 1) and answer for each AI chatbot (Figure 2a, 2b, 2c).

51. Un'auto di massa 1,5 t e velocità di 100 km/h in modulo urta un furgone di massa 4 t e velocità di 80 km/h in modulo, procedendo entrambi nella stessa direzione e verso. Se subito dopo l'urto i due mezzi procedono insieme, come un tutt'uno, determinare ilmodulo della velocità con cui il sistema si muove:

A) circa 85 km/h
B) circa 70 km/h
C) circa 42 km/h
D) circa 31 km/h
E) circa 10 km/h

Figure 1: Example of one of the 820 questions reported in the Word file. In this case, it concerns question number 51 of the CINECA Test of the year 2023/2024 (Physics and Mathematics Test). The correct answer (A) is highlighted in green.

L

Un'auto di massa 1,5 t e velocità di 100 km/h in modulo urta un furgone di massa 4 t e velocità di 80 km/h in modulo, procedendo entrambi nella stessa direzione e verso. Se subito dopo l'urto i due mezzi procedono insieme, come un tutt'uno, determinare il modulo della velocità con cui il sistema si muove:

A) circa 85 km/h
B) circa 70 km/h
C) circa 42 km/h
D) circa 31 km/h
E) circa 10 km/h

Per risolvere questo problema, possiamo utilizzare il principio della conservazione della quantità di moto. La quantità di moto totale prima dell'urto è uguale alla quantità di moto totale dopo l'urto.

La quantità di moto p di un oggetto è definita come:

$$p = m \times v$$

dove m è la massa e v è la velocità.

Prima dell'urto:
Quantità di moto dell'auto p_1 = massa dell'auto m_1 x velocità dell'auto v_1
$p_1 = 1,5t \times 100km/h = 150t \times km/h$

Quantità di moto del furgone p_2 = massa del furgone m_2 x velocità del furgone v_2
$p_2 = 4t \times 80km/h = 320t \times km/h$

Quantità di moto totale prima dell'urto P_{tot_prima} = $p_1 + p_2$
$P_{tot\_prima} = 150t \times km/h + 320t \times km/h = 470t \times km/h$

Dopo l'urto, le due macchine si muovono come un tutt'uno con una massa combinata $m_{combinata}$ e una velocità $v_{combinata}$.

$$m_{combinata} = m_1 + m_2 = 1,5t + 4t = 5,5t$$

La quantità di moto totale dopo l'urto P_{tot_dopo} = $m_{combinata} \times v_{combinata}$

Poiché P_{tot_prima} = P_{tot_dopo} (principio della conservazione della quantità di moto),

$$470t \times km/h = 5,5t \times v_{combinata}$$

Dividendo entrambi i lati per 5,5 t, otteniamo:

$$v_{combinata} = \frac{470}{5,5} km/h = 85,45km/h$$

Questo si arrotonda a:

$$v_{combinata} \approx 85km/h$$

La risposta corretta è:

A) circa 85 km/h.

Rige

Figure 2a: The question was copied from the Word file and pasted into ChatGPT-4. The chatbot provides the correct answer (A).

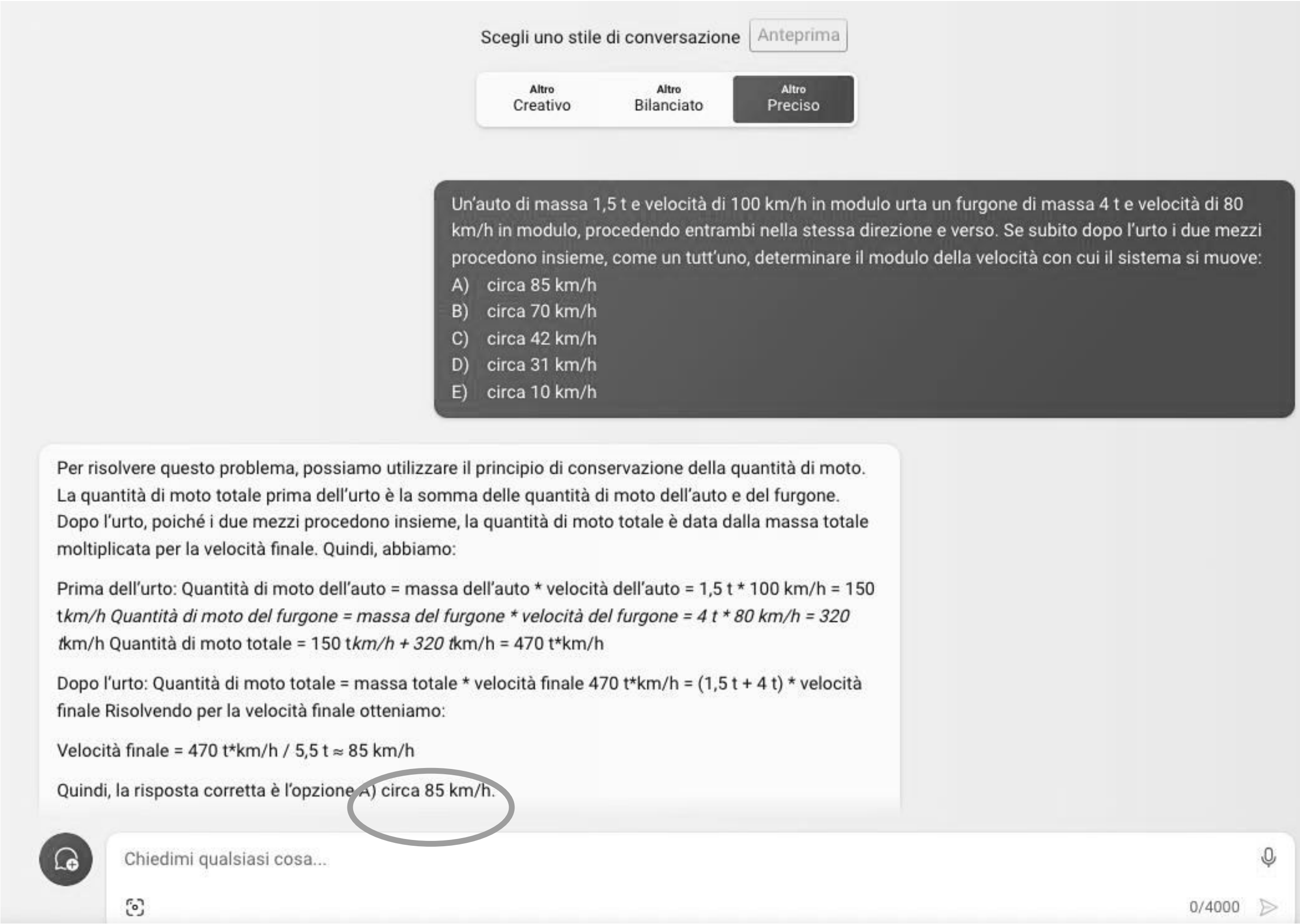

Figure 2b: The question was copied from the Word file and pasted into Bing Chat (conversation style: Precise). The chatbot provides the correct answer (A).

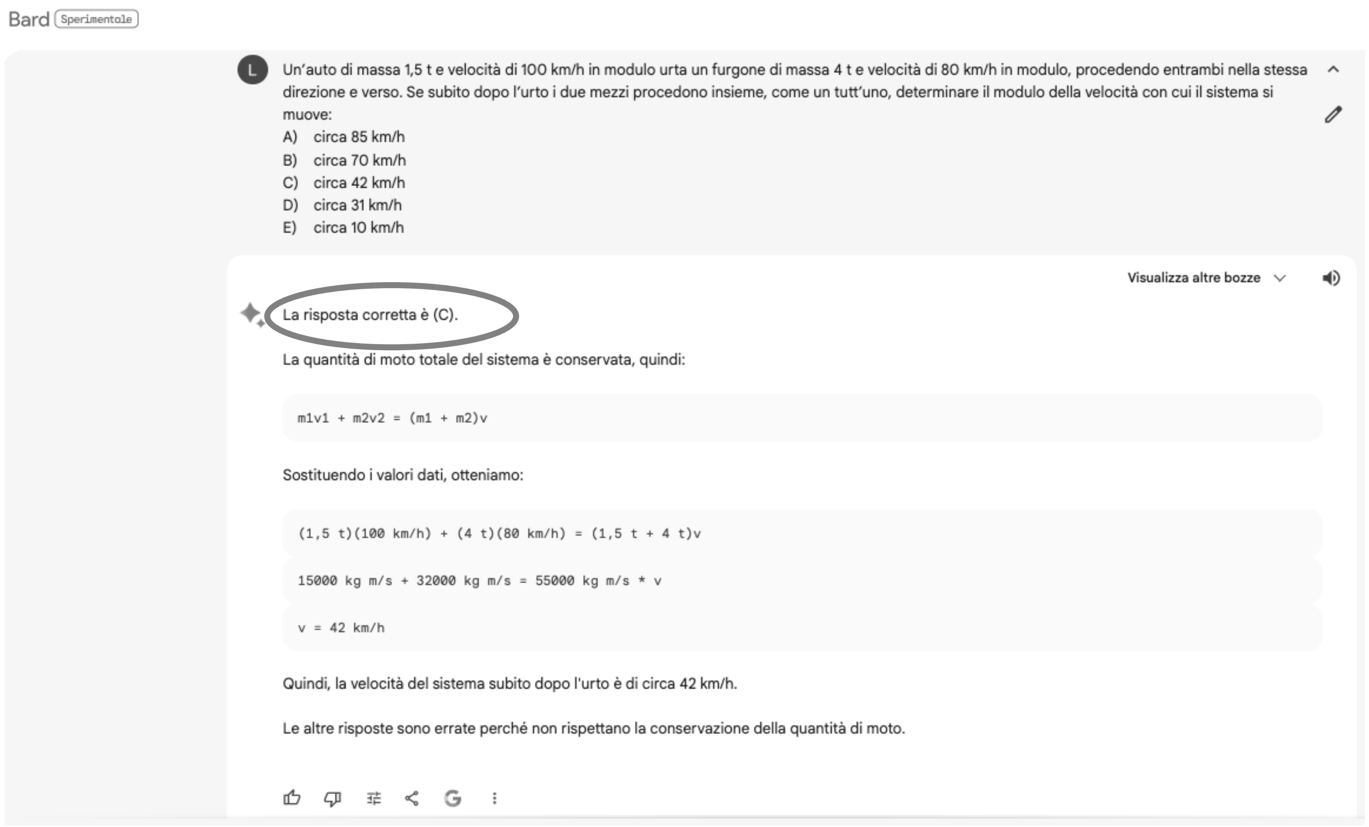

Figure 2c: The question was copied from the Word file and pasted into Google Bard. In this case the chatbot provide an incorrect answer (C).
