# Supplementary material for: Comparative accuracy of ChatGPT-4, Microsoft Copilot and Google Gemini in the Italian entrance test for healthcare sciences degrees: a cross-sectional study
Source: BMC Med Educ. 2024 Jun 26;24:694. doi: 10.1186/s12909-024-05630-9 (PMC11210096; doi:10.1186/s12909-024-05630-9)
Supplement: Supplementary file 4 — Supplementary Material 4. [file 12909_2024_5630_MOESM4_ESM.docx]

## ***Sensitivity analysis***

We performed a sensitivity analysis, excluding answers that were not applicable to each AI chatbot. The data used for this analysis are reported in e-table1. We used the chi-square test to compare chatbots. A statistically significant difference among the three tools was found. We inspected the difference by using Bonferroni, confirming the primary analysis: a statistically significant difference was found between ChatGPT-4 vs Google Bards and Microsoft Bing vs Google Bard. Similar performance was found for ChatGPT-4 and Microsoft Bing. The worst performance was found for Google Bard (e-table 2).

E-table1. Correct answers in the sensitivity analysis

| **Chatbots** | **Not applicable questions** | **Observations run** | **Correct answers** |
| --- | --- | --- | --- |
| ChatGPT-4 (n=820) | 12* | 808 | 763/808 (94,43%) |
| Microsoft Bing (n=820) | 0 | 820 | 737/820 (91,08%) |
| Google Bards (n=820) | 12* | 808 | 574/808 (71,03%) |

*same questions

E-table2. Comparison between AI Chatbots

| **Comparison** | **Chi-square*** | **p-value** |
| --- | --- | --- |
| ChatGPT-4 vs Microsoft Bing | 0,086 | 0,023 |
| ChatGPT-4 vs Google Bards | 0,472 | 0,000 |
| Microsoft Bing vs Google Bard | 0,386 | 0,000 |

*correction with Bonferroni
