# Supplementary material for: Comparative accuracy of ChatGPT-4, Microsoft Copilot and Google Gemini in the Italian entrance test for healthcare sciences degrees: a cross-sectional study
Source: BMC Med Educ. 2024 Jun 26;24:694. doi: 10.1186/s12909-024-05630-9 (PMC11210096; doi:10.1186/s12909-024-05630-9)
Supplement: Supplementary file 5 — Supplementary Material 5. [file 12909_2024_5630_MOESM5_ESM.docx]

**Additional File 5**

Kappa calculates the kappa-statistic measure of interrater agreement for two unique raters. We calculated the interrater agreement for values rater1 and rater2, adding a table of assessments for logical, internal and external answers. The kappa-statistic measure of agreement is scaled to be 0 when the amount of agreement is what would be expected to be observed by chance and 1 when there is perfect agreement. For intermediate values, Landis and Koch (1977) suggest the following interpretations:

below 0.0 Poor

0.00 – 0.20 Slight

0.21 – 0.40 Fair

0.41 – 0.60 Moderate

0.61 – 0.80 Substantial

0.81 – 1.00 Almost perfect

Thus, we obtain almost perfect agreement in all answers between raters: 0.88 in logical, 0.84 in internal and no computation made for external since complete agreement was reported in few rating category.
